# A Facile Semisynthesis and Evaluation of Garcinoic Acid and Its Analogs for the Inhibition of Human DNA Polymerase β

**DOI:** 10.3390/molecules25245847

**Published:** 2020-12-11

**Authors:** Satheesh Gujarathi, Maroof Khan Zafar, Xingui Liu, Robert L. Eoff, Guangrong Zheng

**Affiliations:** 1 Department of Pharmaceutical Sciences, College of Pharmacy, University of Arkansas for Medical Sciences, Little Rock, AR 72205, USA; skgujarathi1323@gmail.com (S.G.); xliu3@cop.ufl.edu (X.L.); 2 Department of Biochemistry and Molecular Biology, College of Medicine, University of Arkansas for Medical Sciences, Little Rock, AR 72205, USA; mzafar2@uams.edu (M.K.Z.); RLEoff@uams.edu (R.L.E.); 3 Department of Medicinal Chemistry, College of Pharmacy, University of Florida, Gainesville, FL 32610, USA

**Keywords:** garcinoic acid, δ-tocotrienol, DNA polymerase β, structure-activity-relationship, semi-synthesis

## Abstract

Garcinoic acid has been identified as an inhibitor of DNA polymerase β (pol β). However, no structure-activity relationship (SAR) studies of garcinoic acid as a pol β inhibitor have been conducted, in part due to the lack of an efficient synthetic method for this natural product and its analogs. We developed an efficient semi-synthetic method for garcinoic acid and its analogs by starting from natural product δ-tocotrienol. Our preliminary SAR studies provided a valuable insight into future discovery of garcinoic acid-based pol β inhibitors.

## 1. Introduction

Base excision repair (BER) is a DNA repair mechanism by which damaged DNA bases and broken DNA single-strands are repaired [1]. BER is a sequential event involving multiple essential enzymes including DNA glycosylase, AP endonuclease, DNA polymerase β (pol β), and DNA ligase [1]. Studies have shown that pol β is involved in repair of the short gaps induced by bleomycin and γ-radiation [2] and its overexpression is responsible for resistance to cisplatin [3,4], whereas down-regulation of pol β by antisense approaches enhances the cytotoxic effects of cisplatin and UV radiation [5]. Moreover, pol β is overexpressed in many cancer cells and 30% of human tumors express pol β variant proteins [6,7]. These findings suggest that pol β is a potential anti-cancer target and underscore the potential of combining pol β inhibitors with chemotherapies as a synergistic therapeutic regimen.

**Figure 1:**
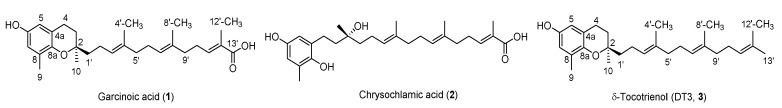
Structures of garcinoic acid (**1**), chrysochlamic acid (**2**), and δ-tocotrienol (DT3, **3**).

During the past two decades, numerous pol β inhibitors have been identified [1,8,9,10], with most of them being natural products harboring moderate potency in inhibiting pol β. Garcinoic acid (**1**, Figure 1), also known as *trans*-13′-carboxy-δ-tocotrienol, is a natural product isolated from *Clusiaceae* family plants, including *Garcinia kola* Heckel seeds and *Garcinia amplexicaulis* Vieill. Ex Pierre bark [11]. Recent studies have highlighted the biological potential of garcinoic acid as an anti-inflammatory agent [12,13], anti-cancer agent [14,15], and pregnane X receptor agonist [16], whereas its pol β inhibition activity was discovered via total synthesis and structure revision of a previous misassigned nature product chrysochlamic acid (**2**, Figure 1) [10]. However, no structure activity relationship studies of garcinoic acid as a pol β inhibitor have been reported, probably due to the lack of synthetic accessibility. Extraction from plant is currently the only way to obtain garcinoic acid, which is not efficient and low yield (0.78%) even with modified extraction procedures [16]. Total synthesis of garcinoic acid has been reported [10]; however, it is challenging to adapt the lengthy (>15 steps) synthetic procedure for the preparation of garcinoic acid analogs. Herein, we present an efficient semi-synthetic method for garcinoic acid and its analogs by using δ-tocotrienol (DT3, **3**) (Figure 1) as a starting material. Our efforts to establish the structure activity relationship (SAR) between garcinoic acid and its pol β inhibition activity is also described here.

## 2. Results and Discussion

**Scheme 1:**
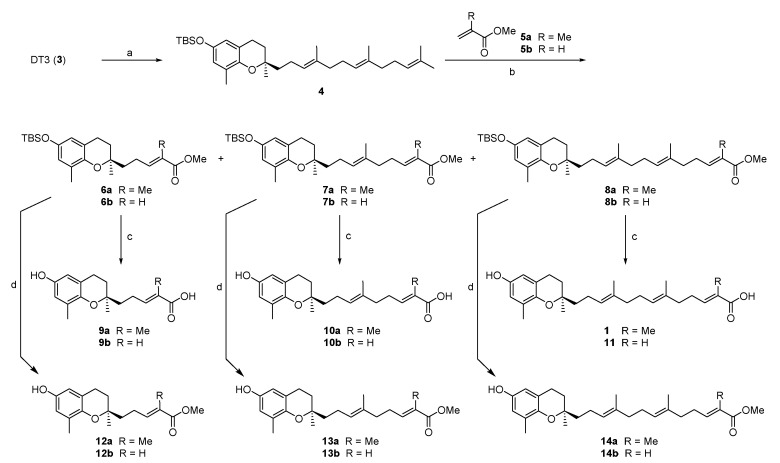
Synthesis of garcinoic acid and its analogs. *Reagents and conditions*: (**a**) imidazole, TBSCl, CH_2_Cl_2_, rt; (**b**) Grubbs catalyst, 2nd generation, toluene, reflux; (**c**) (i) LiOH, THF:MeOH:H_2_O, 45 °C, overnight; (ii) 1N HCl; (**d**) TBAF, THF, 0 °C–rt.

We have been using DT3 (**3**) as a starting material to synthesize its derivatives [17,18,19]. Garcinoic acid can be considered as the long-chain metabolite of DT3 resulting from ω-hydroxylation and ω-oxidation of DT3 and differs from DT3 only by bearing a *trans*-13′-carboxylic acid group [20]. As such, our first attempt to synthesize garcinoic acid and its homologs involves cross metathesis reaction between TBS protected DT3 **4** [17] and methyl methacrylate (**5a**) or methyl acrylate (**5b**) in the presence of Grubbs catalyst, followed by ester group hydrolysis and TBS deprotection (Scheme 1). Although the metathesis reaction led to a mixture of three products, they are readily separable and it was a very efficient approach to obtain garcinoic acid and its homologs with two different tail lengths (Scheme 1). The corresponding carboxylate esters **12**–**14** were obtained by treating **6**–**8** with TBAF to remove the TBS group.

With **4** and **5b** as the reaction partners (Entry 1–8, Table 1), we screened the cross-metathesis reaction conditions, such as changing the catalyst, solvent, and reaction temperature, and found that 5 mol % Grubbs 2nd generation catalyst in toluene at reflux temperature for 3 h afforded the best results. Compounds **6b**, **7b**, and **8b** were obtained in 54%, 14%, and 8% isolated yield, respectively (76% total isolated yield) (Table 1). Similar reaction conditions were applied when **5b** was replaced with **5a** (Entry 9–12, Table 1). Likely due to the increased steric hindrance, the reaction between **4** and **5a** was incomplete after 24 h, which resulted in lower total isolated yield (43%) for **6a**, **7a**, and **8a** (17%, 15%, 11%, respectively). Increase the reaction temperature by changing the reaction solvent from toluene to *o*-xylene did not improve the reaction yield.

Garcinoic acid (**1**) and its homologs, **9a**, **9b**, **10a**, **10b**, and **11**, were tested for their inhibition of human pol β (hpol β) using a well characterized florescence-based polymerase inhibition assay [21] and results are summarized in Table 2. 

**Scheme 2:**
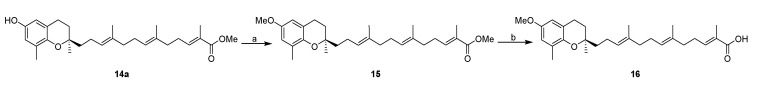
Synthesis of analogs **15** and **16**. *Reagents and conditions*: (**a**) MeI, NaH, DMF, 0 °C–rt, 90%; (–) (i) LiOH, THF:MeOH:H_2_O, 45 °C, overnight; (ii) 1N HCl, 88%.

With an IC_50_ value of 11 µM (Figure S1), the inhibitory activity of garcinoic acid on hpol β is in line with the data reported previously [10]. However, its homologs, **9a**, which contains one isoprene unit, and **10a**, which contains two isoprene units in the side chain, gave IC_50_ values of >120 µM and 52 µM, respectively. These results suggest that the side chain length plays an important role in hpol β inhibition potency of garcinoic acid. Des-methyl analogs **9b**, **10b**, and **11** that contain one methyl group less in the ω-1-position of the side chain demonstrated similar hpol β inhibition activity when compared to their corresponding **9a**, **10a**, and **1**, indicating that this methyl group is not critical for activity. In contrast, the terminal carboxylic acid group is essential for the hpol β inhibition activity as esterification of the terminal carboxylic acid with methyl group (compounds **12**–**14**) abolished the activity. To assess the role of the phenolic OH of garcinoic acid on the hpol β inhibition activity, we synthesized compound **16** by initial conversion of **14a** to **15**, followed by ester hydrolysis (Scheme 2). With the phenolic OH group capped with a methyl group, compound **16** (IC_50_ = 31 µM) exhibited slightly decreased hpol β activity compared with garcinoic acid (IC_50_ = 11 µM), suggesting the OH group of the chromanol head might be tolerable for modification. However, the lack of hpol β inhibition activity by chrysochlamic acid (**2**, Figure 1), a garcinoic acid analog with the dihydropyran ring opened, indicates that the chromane head moiety is important for the hpol β activity of garcinoic acid.

**Scheme 3:**
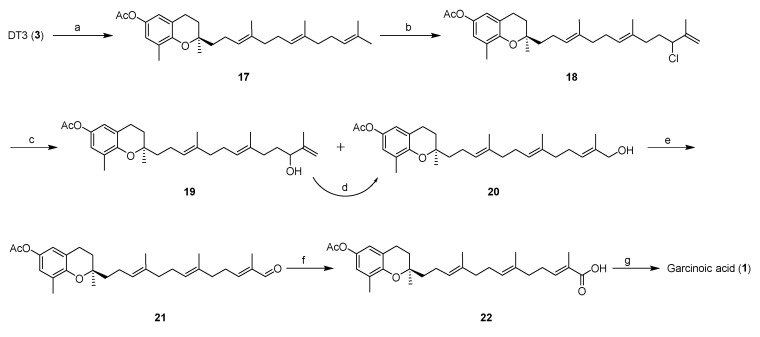
Semi-synthesis of garcinoic acid from δ-tocotrienol. Reagents and conditions: (**a**) Ac_2_O, Et_3_N, CH_2_Cl_2_, 0 °C, 10 h, 90%; (**b**) PhSeCl, NCS, CH_2_Cl_2_, rt, 2 h; (**c**) 2,4,6-collidine, AgBF_4_, acetone, water, 60–70 °C, 4 h, 26% for **19** and 32% for **20**; (**d**) (i) MsCl, pyridine, DMAP, 0 °C–rt, 1 h; (ii) acetone/H_2_O, NaOAc, reflux, 3 h, 34%; (**e**) DMP, CH_2_Cl_2_, 0 °C–rt, 1 h, 92%; (**f**) NaClO_2_, NaH_2_PO_4_, 2-methyl-2-butene, *t*-BuOH/H_2_O, 0 °C–rt, 80%; (**g**) (i) K_2_CO_3_, MeOH; (ii) 1N HCl, 85%.

While the method developed in Scheme 1 provided a rapid path to garcinoic acid and its homologs, the yield of garcinoic acid was low. To the best of our knowledge, only one synthetic method for garcinoic acid has been reported but requires >15 steps [10]. In an effort to provide a facile synthetic route for garcinoic acid with mild conditions and short synthetic sequence, we tried a method to selectively oxidize the terminal methyl group of DT3 (**1**). Our strategy involves a regioselective chlorination of acetyl protected DT3 **17** in the presence of PhSeCl and NCS, followed with an AgBF_4_-mediated hydrolysis of chloride **18** to afford a mixture of alcohols **19** (26%, 2 steps) and **20** (32%, 2 steps) that can be readily separated by column chromatography. The yield of the desired primary alcohol can be further improved by conversion of **19** into **20** via mesylation and subsequent hydrolysis with acetone/H_2_O. Dess–Martin oxidation of alcohol **20** by using Dess–Martin periodinane (DMP) resulted into aldehyde **21**, which was further converted to the corresponding carboxylic acid compound **22** under Pinnick oxidation condition. Removal of the acetyl group on **22** led to the final product garcinoic acid (Scheme 3). In our synthetic route, garcinoic acid was obtained in 6 steps with a total yield of ~18%, which is more efficient than the previously reported method [10].

## 3. Materials and Methods

### 3.1. General Information

All commercially available compounds were used as received. Solvents used for reaction were dried and distilled prior to use from a solvent purification system that purifies solvents by filtration through two columns packed with activated alumina and a 4 Å molecular sieve. If dry and oxygen-free conditions were required, reactions were performed using oven-dried glassware (130 °C) under a positive pressure of argon. Volatile solvents were removed under reduced pressure. Progress of the reactions was monitored by TLC (silica-coated glass plates), visualized under UV light as well as exposure to iodine vapor, and by GC-MS or TLC-MS. Flash column chromatography was performed using silica gel (230–400 mesh) as the stationary phase. NMR spectra were obtained in CDCl_3_ with 0.03% *v*/*v* tetramethylsilane (TMS) on an Agilent 400 MHz spectrometer (Santa Clara, CA, USA). Chemical shifts are reported in parts per million (ppm, δ), using tetramethylsilane as an internal standard. Spectral splitting patterns are designated as s (singlet), *br* s (broad singlet), d (doublet), t (triplet), dd (doublet of doublets), m (multiplet); coupling constants (*J*) are in Hertz (Hz). GC-MS spectra were recorded on an Agilent 6890 GC incorporating an Agilent 7683 autosampler and an Agilent 5973 MSD (Santa Clara, CA, USA). LC-MS spectra were performed on an Advion AVANT LC system with the expression CMS using an Accucore^TM^ Vanquish^TM^ C18 UHPLC column (Thermo, 1.5 µM, 50 × 2.1 mm) (Ithaca, NY, USA) at 40 °C. Gradient elution was used for UHPLC with a mobile phase of acetonitrile and water containing 0.1% formic acid. High resolution mass spectra (HRMS) were recorded on an Agilent 6230 Time-of-Flight (TOF) mass spectrometer (Santa Clara, CA, USA).

### 3.2. Chemistry

#### 3.2.1. Synthesis of tert-butyl({(*R*)-2,8-dimethyl-2-[(3*E*,7*E*)-4,8,12-trimethyltrideca-3,7,11-trien-1-yl]-chroman-6-yl}oxy)dimethylsilane (**4**)

To a solution of δ-tocotrienol (7.9 g, 20.0 mmol) in CH_2_Cl_2_ (120 mL) at 0 °C was added imidazole (3.45 g, 50 mmol) and TBSCl (3.66 g, 24 mmol). The resulting mixture was stirred at room temperature for 5 h. The reaction mixture was partitioned between CH_2_Cl_2_ (100 mL) and water (75 mL). The aqueous layer was extracted with CH_2_Cl_2_ (2 × 50 mL), and the combined organic layers were washed with water (100 mL) and brine (50 mL), dried over anhydrous Na_2_SO_4_, filtered and concentrated under reduced pressure. The crude product was chromatographed on silica (hexanes/ethyl acetate 9:1) to afford compound **4** (9.5 g, 92%) as a colorless oil; ^1^H-NMR (CDCl_3_): δ 6.47 (d, *J* = 2.8 Hz, 1H, phenyl C5-H), 6.37 (d, *J* = 2.8 Hz, 1H, phenyl C7-H), 5.18–5.03 (m, 3H, C3′-H, C7′-H and C11′-H), 2.70 (t, *J* = 7.2 Hz, 2H, C4-H_2_), 2.15–1.96 (m, 13H, C9-H_3_, C2′-H_2_, C5′-H_2_, C6′-H_2_, C9′-H_2_ and C10′-H_2_), 1.81–1.73 (m, 2H, C3-H_2_), 1.69 (s, 3H, C13′-H_3_), 1.54–1.46 (m, 11H, C1′-H_2_, C4′-CH_3_, C8′-CH_3_, and C12′-CH_3_), 1.27 (s, 3H, C10-H_3_), 0.98 (s, 9H, Si-C(CH_3_)_3_), 0.17 (s, 6H, Si(CH_3_)_2_); ^13^C-NMR (CDCl_3_): *δ* 147.6 (phenyl C-6), 146.4 (phenyl C-8a), 135.2 (C-8′), 135.0 (C-4′), 131.3 (C-12′), 126.9 (phenyl C-8), 124.5 (C-3′), 124.4 (C-7′), 124.3 (C-11′), 120.9 (phenyl C-7), 120.2 (phenyl C-4a), 117.3 (phenyl C-5), 75.4 (C-2), 39.8 (3C, C-1′, C-5′ and C-9′), 31.6 (C-3), 26.9 (2C, C-6′ and C-10′), 26.7 (C-2′), 25.8 (Si-*C*(CH_3_)_3_), 24.2 (C-10), 22.6 (C-13′), 22.3 (C-4), 18.2 (Si-C(*C*H*_3_*)_3_), 17.8 (C12′-*C*H_3_), 16.2 (C8′-CH_3_), 16.1 (C4′-CH_3_), 15.9 (C-9), −4.3 (Si(*C*H_3_)_2_); GC-MS (EI) *m*/*z* 510.5 (M^+^, 100), 291.1, 251.1, 193.1, 69.1.

#### 3.2.2. General Procedure for the Synthesis of **6a**, **7a**, **8a**, **6b**, **7b**, and **8b**

Compound **4** (2.0 g, 4 mmol) and **5a** (2.40 g, 24 mmol) or **5b** (2.00 g, 24 mmol) were taken in toluene (30 mL) and heated to 80 °C. At this temperature Grubbs II catalyst was added and the reaction was heated at reflux for 24 h. Solvent was removed under reduced pressure and the residue was subjected to column chromatography using hexanes/ethyl acetate (98:2) to afford **6a**, **7a**, and **8a** or **6b**, **7b**, and **8b** as colorless oils.

*Methyl 5-{(R)-6-[(tert-butyldimethylsilyl)oxy]-2,8-dimethylchroman-2-yl}-2-methylpent-2E-enoate* (**6a**), colorless oil; Yield 17%; ^1^H-NMR (CDCl_3_): δ 6.78 (t, *J* = 1.2 Hz, 1H, C3′-H), 6.46 (d, *J* = 2.8 Hz, 1H, phenyl C5-H), 6.37 (d, *J* = 2.8 Hz, 1H, phenyl C7-H), 3.71 (s, 3H, OCH_3_), 2.78–2.58 (m, 2H, C4-H_2_), 2.37–2.25 (m, 2H, C2′-H_2_), 2.10 (s, 3H, C9-H_3_), 1.82 (s, 3H, C4′-CH_3_), 1.90–1.57 (m, 4H, C3-H_2_ and C1′-H_2_), 1.26 (s, 3H, C10-H_3_), 0.97 (s, 9H, (Si-C(CH_3_)_3_), 0.16 (s, 6H, Si(CH_3_)_2_); ^13^C-NMR (CDCl_3_): δ 168.7 (C=O), 147.8 (phenyl C-6), 146.1 (phenyl C-8a), 142.6 (C-3′), 127.6 (C-4′), 126.9 (phenyl C-8), 120.7 (phenyl C-7), 120.3 (phenyl C-4a), 117.3 (phenyl C-5), 74.9 (C-2), 51.7 (OCH_3_), 38.4 (C-1′), 31.6 (C-3), 25.8 (Si-*C*(CH_3_)_3_), 23.9 (C-10), 23.0 (C-2′), 22.4 (C-4), 18.2 (Si-C(*C*H_3_)_3_), 16.1 (C-9), 12.3 (C4′-*C*H_3_), -4.33 (Si(CH_3_)_2_; GC-MS (EI) *m*/*z* 418.3 (M^+^, 100), 387.3, 361.2, 291.1, 251.2, 225.1, 193.1, 73.1.

*Methyl 9-{(R)-6-[(tert-butyldimethylsilyl)oxy]-2,8-dimethylchroman-2-yl}-2E,6E-dimethylnona-2E,6E-dienoate* (**7a**), colorless oil; Yield 15%; ^1^H-NMR (CDCl_3_): δ 6.73 (t, *J* = 1.6 Hz, 1H, C7′-H), 6.43 (d, *J* = 2.0 Hz, 1H, phenyl C5-H), 6.36 (d, *J* = 2.0 Hz, 1H, phenyl C7-H), 5.16 (t, *J* = 6.4 Hz, 1H, C3′-H), 3.72 (s, 3H, OCH_3_), 2.66 (t, *J* = 5.2 Hz, 2H, C4-H_2_), 2.65–2.04 (m, 9H, C9-H_3_, C2′-H_2_, C5′-H_2_ and C6′-H_2_), 1.82 (s, 3H, C8′-CH_3_), 1.81–1.45 (m, 7H, C1′-H_2_, C3-H_2_ and C4′-CH_3_), 1.25 (s, 3H, C10-H_3_), 0.96 (s, 9H, Si-C(CH_3_)_3_), 0.15 (s, 6H, Si(CH_3_)_2_); ^13^C-NMR (CDCl_3_): δ 168.8 (C=O), 147.7 (phenyl C-6), 146.4 (phenyl C-8a), 142.3 (C-7′), 134.1 (C-4′), 127.6 (C-8′), 126.9 (phenyl C-8), 125.4 (C-3′), 120.9 (phenyl C-7), 120.2 (phenyl C-4a), 117.3 (phenyl C-5), 75.3 (C-2), 51.8 (OCH_3_), 39.7 (C-1′), 38.3 (C-5′), 31.6 (C-3), 27.4 (C-6′), 25.8 (Si-*C*(CH_3_)_3_), 24.1 (C-10), 22.6 (C-2′), 22.3 (C-4), 18.2 (Si-C(*C*H_3_)_3_), 16.2 (C-9), 15.9 (C4′-*C*H_3_), 12.5 (C8′-*C*H_3_), -4.30 (Si(*C*H_3_)_2_); GC-MS (EI) *m*/*z* 486.4 (M^+^, 100), 455.3, 429.3, 291.1, 251.1 225.1, 193.1, 73.1.

*Methyl 13-{(R)-6-[(tert-butyldimethylsilyl)oxy]-2,8-dimethylchroman-2-yl}-2,6,10-trimethyltrideca-2E,6E,10E-trienoate* (**8a**), colorless oil; Yield 11%; ^1^H-NMR (CDCl_3_): δ 6.74 (t, *J* = 1.6 Hz, 1H, C11′-H), 6.45 (d, *J* = 2.8 Hz, 1H, phenyl C5-H), 6.36 (d, *J* = 2.8 Hz, 1H, phenyl C7-H), 5.11 (t, *J* = 7.2 Hz, 2H, C3′-H and C7′-H), 3.72 (s, 3H, OCH_3_), 2.66 (t, *J* = 5.6 Hz, 2H, C4-H_2_), 2.35–2.22 (m, 2H, C10′-H_2_), 2.15–1.95 (m, 11H, C9-H_3_, C2′-H_2_, C5′-H_2_, C6′-H_2_ and C9′-H_2_), 1.83 (s, 3H, C12′-CH_3_), 1.80–1.45 (m, 10H, C1′-H_2_, C3-H_2_, C4′-CH_3_ and C8′-CH_3_), 1.25 (s, 3H, C10-H_3_), 0.96 (s, 9H, Si-C*(*CH_3_)_3_), 0.15 (s, 6H, Si(CH_3_)_2_); ^13^C-NMR (CDCl_3_): δ 168.8 (C=O), 147.6 (phenyl C-6), 146.4 (phenyl C-8a), 142.4 (C-11′), 135.1 (C-8′), 134.0 (C-4′), 127.5 (C-12′), 126.9 (phenyl C-8), 125.2 (C-3′), 124.5 (C-7′), 120.9 (phenyl C-7), 120.2 (phenyl C-4a), 117.3 (phenyl C-5), 75.3 (C-2), 51.8 (OCH_3_), 39.8 (C-5′), 39.7 (C-1′), 38.3 (C-9′), 31.6 (C-3), 27.5 (C-10′), 26.7 (C-6′), 25.8 (Si-*C*(CH_3_)_3_), 24.2 (C-10), 22.6 (C-2′), 22.3 (C-4), 18.2 (Si-C(*C*H_3_)_3_), 16.2 (C-9), 16.1 (C4′-*C*H_3_), 15.9 (C8′-CH_3_), 12.5 (C12′-*C*H_3_), −4.30 (Si(*C*H_3_)_2_); GC-MS (EI) *m*/*z* 554.5 (M^+^), 523.4, 497.4, 291.1, 251.1 225.1, 193.1, 73.1.

*Methyl 5-{(R)-6-[(tert-butyldimethylsilyl)oxy]-2,8-dimethylchroman-2-yl}pent-2E-enoate* (**6b**), colorless oil; Yield 54%; ^1^H-NMR (CDCl_3_): δ 7.04–6.96 (m, 1H, C3′-H), 6.45 (d, *J* = 2.4 Hz, 1H, phenyl C5-H), 6.36 (d, *J* = 2.4 Hz, 1H, phenyl C7-H), 5.83 (d, *J* = 16.0 Hz, 1H, C4′-H), 3.71 (s, 3H, OCH_3_), 2.66 (t, *J* = 5.6 Hz, 2H, C4-H_2_), 2.25–2.18 (m, 2H, C2′-H_2_), 2.09 (s, 3H, C9-H_3_), 1.82–1.57 (m, 4H, C3-H_2_ and C1′-H_2_), 1.25 (s, 3H, C10-H_3_), 0.95 (s, 9H, Si-C(CH_3_)_3_), 0.15 (s, 6H, Si(CH*_3_*)_2_); ^13^C-NMR (CDCl_3_): δ 167.2 (C=O), 149.8 (C-3′), 147.9 (phenyl C-6), 146.1 (phenyl C-8a), 127.0 (phenyl C-8), 120.9 (C-4′), 120.7 (phenyl C-7), 120.3 (phenyl C-4a), 117.3 (phenyl C-5), 74.8 (C-2), 51.5 (OCH_3_), 38.2 (C-1′), 31.7 (C-3), 25.8 (Si-*C*(CH_3_)_3_), 23.9 (C-10), 22.6 (C-2′), 22.5 (C-4), 18.2 (Si-C(*C*H_3_)_3_), 16.2 (C-9), −4.3 (Si-(CH_3_)_2_); GC-MS (EI) *m*/*z* 404.3 (M^+^, 100), 373.3, 347.2, 291.1, 251.2, 225.1, 193.1, 73.1. 

*Methyl 9-{(R)-6-[(tert-butyldimethylsilyl)oxy]-2,8-dimethylchroman-2-yl}-6-methylnona-2E,6E-dienoate* (**7b**), colorless oil; Yield 14%; ^1^H-NMR (CDCl_3_): δ 6.99–6.85 (m, 1H, C7′-H), 6.45 (d, *J* = 2.8, 1H, phenyl C5-H), 6.36 (d, *J* = 2.8, 1H, phenyl C7-H), 5.88 (d, *J* = 15.4 Hz, 1H, C8′-H), 5.16 (t, *J* = 1.2 Hz, 1H, C3′-H), 3.71 (s, 3H, OCH_3_), 2.75–2.62 (m, 2H, C4-H_2_), 2.35–2.18 (m, 2H, C6′- H_2_), 2.16–2.03 (m, 7H, C9-H_3_, C2′-H_2_, and C5′-H_2_), 1.82–1.45 (m, 7H, C1′-H_2_, C3-H_2_ and C4′-CH_3_), 1.25 (s, 3H, C10-H_3_), 0.96 (s, 9H, Si-C(CH_3_)_3_), 0.16 (s, 6H, Si(CH_3_)_2_); ^13^C-NMR (CDCl_3_): δ 167.2 (C=O), 149.4 (C-7′), 147.6 (phenyl C-6), 146.4 (phenyl C-8a), 133.6 (C-4′), 126.9 (phenyl C-8), 125.6 (C-3′), 121.0 (C-8′), 120.9 (phenyl C-7), 120.2 (phenyl C-4a), 117.3 (phenyl C-5), 75.2 (C-2), 51.5 (OCH_3_), 39.7 (C-1′), 38.0 (C-5′), 31.6 (C-3), 30.8 (C-6′), 25.8 (Si-*C*(CH_3_)_3_), 24.1 (C-10), 22.5 (C-2′), 22.2 (C-4), 18.2 (Si-C(*C*H_3_)_3_), 16.2 (C-9), 15.8 (C4′-*C*H_3_), −4.3 (Si-(CH_3_)_2_); GC-MS (EI) *m*/*z* 472.4 (M^+^, 100), 441.3, 415.3, 291.1, 251.2, 225.1, 193.1, 73.1. 

*Methyl 13-{(R)-6-[(tert-butyldimethylsilyl)oxy]-2,8-dimethylchroman-2-yl}-6,10-dimethyltrideca-2E,6E,10E-trienoate* (**8b**), colorless oil; Yield 5%; ^1^H-NMR (CDCl_3_): δ 6.99–6.85 (m, 1H, C11′-H), 6.45 (d, *J* = 2.4 Hz, 1H, phenyl C5-H), 6.36 (d, *J* = 2.4 Hz, 1H, phenyl C7-H), 5.81 (d, *J* = 15.6 Hz, 1H, C12′-H), 5.12 (t, *J* = 6.4 Hz, 2H, C3′-H and C7′-H), 3.71 (s, 3H, OCH_3_), 2.67 (t, *J* = 6.8 Hz, 2H, C4-H_2_), 2.18–1.45 (m, 23H, C9-H_3_, C3-H_2_, C1′-H_2_, C2′-H_2_, C5′-H_2_, C6′-H_2_, C9′-H_2_, C10′-H_2_, C4′-CH_3_ and C8′-CH_3_), 1.25 (s, 3H, C10-H_3_), 0.96 (s, 9H, Si-C(CH_3_)_3_), 0.16 (s, 6H, Si(CH_3_)_2_); ^13^C-NMR (CDCl_3_): δ 167.2 (C=O), 149.4 (C-11′), 147.6 (phenyl C-6), 146.4 (phenyl C-8a), 135.0 (C-8′), 133.5 (C-4′), 126.9 (phenyl C-8), 125.4 (C-3′), 124.6 (C-7′), 121.0 (C-12′), 120.9 (phenyl C-7), 120.2 (phenyl C-4a), 117.3 (phenyl C-5), 75.3 (C-2), 51.5 (OCH_3_), 39.8 (C-5′), 39.6 (C-1′), 38.0 (C-9′), 31.5 (C-3), 26.6 (C-6′), 26.0 (C-10′), 25.8 (Si-*C*(CH_3_)_3_), 24.2 (C-10), 22.6 (C-2′), 22.2 (C-4), 18.2 (Si-C(*C*H_3_)_3_), 16.2 (C-9), 16.0 (C4′-*C*H_3_), 15.9 (C8′-CH_3_), −4.3 (Si(CH_3_)_2_); GC-MS (EI) *m*/*z* 540.5 (M^+^, 100), 509.4, 483.4, 291.1, 251.2, 225.1, 193.1, 135.1, 107.1, 73.1.

#### 3.2.3. General Procedure for the Synthesis of **9a**, **10a**, **1**, **9b**, **10b**, and **11**

To a stirred solution of compound **6a**, **7a**, **8a**, **6b**, **7b**, or **8b** (0.1 mmol) in THF-MeOH-H_2_O (3:1:1, 4 mL) was added (0.3 mmol) of LiOH. The reaction mixture was heated at 40 °C under N_2_ for 14 h. After cool to room temperature, the reaction mixture was diluted with ethyl acetate (5 mL) and treated with of 1 N HCl (5 mL). The aqueous layer was extracted with ethyl acetate (2 × 10 mL). The combined organic layers were washed with brine (10 mL), dried over anhydrous Na_2_SO_4_, and concentrated under reduced pressure. The residue was purified by silica gel column using 1:1 hexanes-ethyl acetate to afford compound **9a**, **10a**, **1**, **9b**, **10b**, and **11**, respectively, as colorless oils.

*5-[(R)-6-Hydroxy-2,8-dimethylchroman-2-yl]-2-methylpent-2E-enoic acid* (**9a**), colorless oil; Yield 88%; ^1^H-NMR (CDCl_3_): δ 6.92 (t, *J* = 1.2 Hz, 1H, C3′-H), 6.48 (d, *J* = 2.8 Hz, 1H, phenyl C5-H), 6.39 (d, *J* = 2.8 Hz, 1H, Phenyl C7-H), 2.78–2.58 (m, 2H, C4-H_2_), 2.40–2.26 (m, 2H, C2′-H_2_), 2.12 (s, 3H, C9-H_3_), 1.86–1.61 (m, 7H, C1′-H_2_, C3-H_2_ and C4′-CH_3_), 1.26 (s, 3H, C10-H_3_); ^13^C-NMR (CDCl_3_): δ 173.2 (C=O), 148.0 (phenyl C-6), 145.8 (C-3′), 145.2 (phenyl C-8a), 127.5 (C-4′), 127.1 (phenyl C-8), 121.1 (phenyl C-4a), 115.9 (phenyl C-7), 112.7 (phenyl C-5), 75.0 (C-2), 38.3 (C-1′), 31.6 (C-3), 23.9 (C-10), 23.3 (C-4), 22.5 (C-2′), 16.1 (C-9), 12.0 (C4′-*C*H_3_); HRMS (ESI) *m*/*z* = 313.1410 calcd. for C_17_H_22_O_4_Na [M + Na]^+^; found: 313.1405.

*9-[(R)-6-Hydroxy-2,8-dimethylchroman-2-yl]-2,6-dimethylnona-2E,6E-dienoic acid* (**10a**), colorless oil; Yield 84%; ^1^H-NMR (CDCl_3_): δ 6.86 (t, *J* = 6.8 Hz, 1H, C7′-H), 6.47 (d, *J* = 2.4 Hz, 1H, phenyl C5-H), 6.38 (d, *J* = 2.4 Hz, 1H, phenyl C7-H), 5.16 (t, *J* = 6.4 Hz, 1H, C3′-H), 2.68 (t, *J* = 6.4 Hz, 2H, C4-H_2_), 2.34–2.21 (m, 2H, C6′-H_2_), 2.17–2.01 (m, 7H, C9-H_3_, C2′-H_2_ and C5′-CH_3_), 1.82–1.48 (m, 10H, C1′-H_2_, C3-H_2_, C4′-CH_3_ and C8′-CH_3_), 1.27 (s, 3H, C10-H_3_); ^13^C-NMR (CDCl_3_): δ173.1 (C=O), 147.8 (phenyl C-6), 146.0 (C-7′), 145.0 (phenyl C-8a), 133.9 (C-4′), 127.4 (C-8′), 127.0 (phenyl C-8), 125.5 (C-3′), 121.3 (phenyl C-4a), 115.7 (phenyl C-7), 112.7 (phenyl C-5), 75.3 (C-2), 39.5 (C-5′), 38.1 (C-1′), 31.5 (C-3), 27.5 (C-6′), 24.1 (C-10), 22.5 (C-4), 22.2 (C-2′), 16.2 (C-9), 15.9 (C4′-*C*H_3_), 12.1 (C8′-*C*H_3_); HRMS (ESI) *m*/*z* = 381.2036 calcd. for C_22_H_30_O_4_Na [M + Na]^+^; found: 381.2036.

*13-[(R)-6-Hydroxy-2,8-dimethylchroman-2-yl]-2,6,10-trimethyltrideca-2E,6E,10E-trienoic acid* (**1**), colorless oil; Yield 87%; ^1^H-NMR (CDCl_3_): δ 6.86 (t, *J* = 6.0 Hz, 1H, C11′-H), 6.47 (d, *J* = 2.4 Hz, 1H, phenyl C5-H), 6.38 (d, *J* = 2.4 Hz, 1H, phenyl C7-H), 5.12 (t, *J* = 7.2 Hz, 2H, C3′-H and C7′-H), 2.69 (t, *J* = 6.8 Hz, 2H, C4-H_2_), 2.35–2.24 (m, 2H, C10′-H_2_), 2.16–1.92 (m, 11H, C2′-H_2_, C5′-H_2_, C6′-H_2_, C9′-H_2_ and C9-H_3_), 1.85–1.45 (m, 13H, C1′-H_2_, C3-H_2_, C4′-CH_3_, C8′-CH_3_ and C12′- CH_3_), 1.26 (s, 3H, C10-H_3_); ^13^C-NMR (CDCl_3_): δ 172.0 (C=O), 147.8 (phenyl C-6), 146.1 (C-11′), 145.0 (phenyl C-8a), 135.0 (C-8′), 133.8 (C-4′), 127.5 (C-12′), 126.8 (phenyl C-8), 125.3 (C-3′), 124.6 (C-7′), 121.3 (phenyl C-4a), 115.7 (phenyl C-7), 112.7 (phenyl C-5), 75.4 (C-2), 39.8 (C-5′), 39.6 (C-1′), 38.1 (C-9′), 31.5 (C-3), 27.6 (C-6′), 26.6 (C-10′), 24.2 (C-10), 22.6 (C-4), 22.3 (C-2′), 16.2 (C-9), 16.1 (C8′-*C*H_3_), 15.9 (C4′-*C*H_3_), 12.2 (C12′-*C*H_3_); MS (ESI) *m*/*z* 425.2 [M − H]^−^; HRMS (ESI) *m*/*z* = 449.2662 calcd. for C_27_H_38_O_4_Na [M + Na]^+^; found: 449.2662. The NMR data are in line with those reported [10].

*5-[(R)-6-Hydroxy-2,8-dimethylchroman-2-yl]pent-2E-enoic acid* (**9b**), colorless oil; Yield 83%; ^1^H-NMR (CDCl_3_): δ 7.11–7.02 (m, 1H, C3′-H), 6.47 (s, 1H, phenyl C5-H), 6.38 (s, 1H, phenyl C7-H), 5.83 (d, *J* = 16 Hz, 1H, C4′-H), 2.78–2.58 (m, 2H, C4-H_2_), 2.44–2.39 (m, 2H, C2′-H_2_), 2.10 (s, 3H, C9-H_3_), 1.85–1.59 (m, 4H, C1′-H_2_ and C3-H_2_), 1.25 (s, 3H, C10-H_3_); ^13^C-NMR (CDCl_3_): δ 171.6 (C=O), 152.5 (C-3′), 148.0 (phenyl C-6), 145.7 (phenyl C-8a), 127.5 (phenyl C-8), 121.1 (phenyl C-4a), 120.5 (C-4′), 115.9 (phenyl C-7), 112.7 (phenyl C-5), 74.8 (C-2), 38.0 (C-1′), 31.6 (C-3), 26.7 (C-10), 23.9 (C-4), 22.4 (C-2′), 16.2 (C-9); HRMS (ESI) *m*/*z* = 299.1254 calcd. for C_16_H_20_O_4_Na [M + Na]^+^; found: 299.1244.

*9-[(R)-6-Hydroxy-2,8-dimethylchroman-2-yl]-6-methylnona-2E,6E-dienoic acid* (**10b**), colorless oil; Yield 85% yield; ^1^H-NMR (CDCl_3_): δ 7.07–6.95 (m, 1H, C7′-H), 6.47 (d, *J* = 2.4 Hz, 1H, phenyl C5-H), 6.38 (d, *J* = 2.4 Hz, 1H, phenyl C7-H), 5.80 (d, *J* = 15.6 Hz, 1H, C8′-H), 5.16 (t, *J* = 6.8 Hz, 1H, C3′-H), 2.68 (t, *J* = 7.6 Hz, 2H, C4-H_2_), 2.37–2.25 (m, 2H, C6′-H_2_), 2.11–2.04 (m, 7H, C9-H_3_, C2′-H_2_ and C5′-H_2_), 1.82–1.45 (m, 7H, C1′-H_2_, C3-H_2_ and C4′-CH_3_), 1.25 (s, 3H, C10-H_3_); ^13^C-NMR (CDCl_3_): δ 171.6 (C=O), 152.0 (C-7′), 147.8 (phenyl C-6), 146.0 (phenyl C-8a), 133.5 (C-4′), 127.4 (phenyl C-8), 125.7 (C-8′), 121.3 (phenyl C-4a), 120.7 (C-3′), 115.7 (phenyl C-7), 112.7 (phenyl C-5), 75.3 (C-2), 39.5 (C-5′), 37.8 (C-1′), 31.5 (C-3), 30.9 (C-6′), 24.1 (C-10), 22.5 (C-4), 22.2 (C-2′), 16.2 (C-9), 15.9 (C4′-*C*H_3_); HRMS (ESI) *m*/*z* = 367.1880 calcd. for C_21_H_28_O_4_Na [M + Na]^+^; found: 367.1865.

*13-[(R)-6-Hydroxy-2,8-dimethylchroman-2-yl]-6,10-dimethyltrideca-2E,6E,10E-trienoic acid* (**11**), colorless oil; Yield 83%; ^1^H-NMR (CDCl_3_): δ 7.07–6.96 (m, 1H, C11′-H), 6.46 (d, *J* = 2.4, 1H, phenyl C5-H), 6.37 (d, *J* = 2.4 Hz, 1H, phenyl C7-H), 5.80 (d, *J* = 15.6 Hz, 1H, C12′-H), 5.11 (t, *J* = 6.8 Hz, 2H, C3′-H and C7′-H), 2.68 (t, *J* = 6.8 Hz, 2H, C4-H_2_), 2.35–2.18 (m, 2H, C10′-H_2_), 2.25–1.92 (m, 11H, C9-H_3_, C2′-H_2_, C5′-H_2_, C6′-H_2_ and C9′-H_2_), 1.85–1.51 (m, 10H, C1′-H_2_, C3-H_2_, C4′-CH_3_ and C8′-CH_3_), 1.25 (s, 3H, C10-H_3_); ^13^C-NMR (CDCl_3_): δ 171.1 (C=O), 152.2 (C-11′), 147.8 (phenyl C-6), 146.0 (phenyl C-8a), 134.9 (C-8′), 133.4 (C-4′), 127.5 (phenyl C-8), 125.5 (C-3′), 124.6 (C-7′), 121.3 (phenyl C-4a), 120.5 (C-12′), 115.8 (phenyl C-7), 112.7 (phenyl C-5), 75.4 (C-2), 39.6 (C-5′), 39.5 (C-9′), 37.8 (C-1′), 31.5 (C-3), 31.0 (C-6′), 26.5 (C-10′), 24.2 (C-10), 22.6 (C-4), 22.3 (C-2′), 16.2 (C8′-*C*H_3_), 16.0 (C4′-*C*H_3_), 15.9 (C4′-*C*H_3_); HRMS (ESI) *m*/*z* = 435.2506 calcd. for C_26_H_36_O_4_Na [M + Na]^+^; found: 435.2511.

#### 3.2.4. General Procedure for the Synthesis of Compounds **12a**, **13a**, **14a**, **12b**, **13b**, and **14b**

To a stirred solution of compound **6a**, **7a**, **8a**, **6b**, **7b**, or **8b** (0.1 mmol) in anhydrous THF (3 mL) at 0 °C under argon atmosphere was added TBAF (0.2 mL, 1M in THF, 0.2 mmol) dropwise, and allowed to stir at room temperature until TLC revealed full consumption of the starting material (2 h). Reaction mixture was quenched by adding water (2 mL) and extracted with ethyl acetate (2 × 5 mL). Combined organic layers were dried over anhydrous Na_2_SO_4_, and solvents were removed under reduced pressure. The residue was purified by silica gel column using hexanes-ethyl acetate (6:4) to afford compound **12a**, **13a**, **14a**, **12b**, **13b**, and **14b**, respectively, as colorless oils.

*Methyl 5-[(R)-6-hydroxy-2,8-dimethylchroman-2-yl]-2-methylpent-2E-enoate* (**12a**), colorless oil; Yield 91%; ^1^H-NMR (CDCl_3_): δ 6.79 (dt, *J* = 1.6, 7.6 Hz, 1H, C3′-H), 6.48 (d, *J* = 3.2 Hz, 1H, phenyl C5-H), 6.39 (d, *J* = 3.2 Hz, 1H, phenyl C7-H), 4.70 (*br* s, 1H, OH), 3.72 (s, 3H, OCH_3_), 2.78–2.58 (m, 2H, C4-H_2_), 2.38–2.26 (m, 2H, C2′-H_2_), 2.11 (s, 3H, C9-H_3_), 1.83 (s, 3H, C4′-CH_3_), 1.82–1.52 (m, 4H, C3-H_2_ and C1′-H_2_), 1.26 (s, 3H, C10-H_3_); ^13^C-NMR (CDCl_3_): δ 168.9 (C=O), 148.1 (phenyl C-6), 145.7 (phenyl C-8a), 142.8 (C-3′), 127.6 (C-4′), 127.4 (phenyl C-8), 121.1 (phenyl C-7), 115.9 (phenyl C-4a), 112.7 (phenyl C-5), 75.0 (C-2), 51.8 (OCH_3_), 38.4 (C-1′), 31.6 (C-3), 23.9 (C-10), 23.1 (C-4), 22.5 (C-2′), 16.1 (C-9), 12.3 (C4′-*C*H_3_); HRMS (ESI) *m*/*z* = 327.1567 calcd. for C_18_H_24_O_4_Na [M + Na]^+^; found: 327.1577.

*Methyl 9-[(R)-6-hydroxy-2,8-dimethylchroman-2-yl]-2,6-dimethylnona-2E,6E-dienoate* (**13a**). colorless oil; Yield 90%; ^1^H-NMR (CDCl_3_): δ 6.72 (t, *J* = 1.2 Hz, 1H, C7′-H), 6.48 (d, *J* = 2.8 Hz, 1H, phenyl C5-H), 6.38 (d, *J* = 2.8 Hz, 1H, phenyl C7-H), 5.16 (t, *J* = 1.2 Hz, 1H, C3′-H), 4.27 (*br* s, 1H, OH), 3.73 (s, 3H, OCH_3_), 2.74–2.65 (m, 2H, C4-H_2_), 2.31–2.20 (m, 2H, C6′-H_2_), 2.17–2.02 (m, 7H, C9-H_3_, C2′-H_2_ and C5′-H_2_), 1.84–1.51 (m, 10H, C3-H_2_, C1′-H_2_, C4′-CH_3_ and C8′-CH_3_), 1.26 (s, 3H, C10-H_3_); ^13^C-NMR (CDCl_3_): δ 168.8 (C=O), 147.9 (phenyl C-6), 146.1 (phenyl C-8a), 142.4 (C-7′), 134.1 (C-4′), 127.6 (C-8′), 127.5 (phenyl C-8), 125.3 (C-3′), 121.3 (phenyl C-7), 115.7 (phenyl C-4a), 112.7 (phenyl C-5), 75.4 (C-2), 51.8 (OCH_3_), 39.7 (C-1′), 38.3 (C-5′), 31.5 (C-3), 27.4 (C-6′), 24.1 (C-10), 22.6 (C-2′), 22.3 (C-4), 16.2 (C-9), 15.9 (C4′-*C*H_3_), 12.5 (C8′-*C*H_3_); HRMS (ESI) *m*/*z* = 395.2193 calcd. for C_23_H_32_O_4_Na [M + Na]^+^; found: 395.2191.

*Methyl 13-[(R)-6-hydroxy-2,8-dimethylchroman-2-yl]-2,6,10-trimethyltrideca-2E,6E,10E-trienoate* (**14a**), colorless oil; Yield 91%; ^1^H-NMR (CDCl_3_): δ 6.74 (t, *J* = 6.8 Hz, 1H, C11′-H), 6.48 (d, *J* = 2.8 Hz, 1H, phenyl C5-H), 6.38 (d, *J* = 2.8 Hz, 1H, phenyl C7-H), 5.16–5.08 (m, 2H, C3′-H and C7′-H), 4.49 (*br* s, 1H, OH), 3.73 (s, 3H, OCH_3_), 2.68 (t, *J* = 6.8 Hz, 2H, C4-H_2_), 2.28–2.21 (m, 2H, C10′-H_2_), 2.15–1.92 (m, 11H, C9-H_3_, C2′-H_2_, C5′-H_2_, C6′-H_2_ and C9′-H_2_), 1.82 (s, 3H, C12′-CH_3_), 1.81–1.50 (m, 10H, C3-H_2_, C1′-H_2_, C4′-CH_3_ and C8′-CH_3_), 1.26 (s, 3H, C10-H_3_); ^13^C-NMR (CDCl_3_): δ 168.9 (C=O), 147.9 (phenyl C-6), 146.0 (phenyl C-8a), 142.6 (C-11′), 135.0 (C-8′), 134.0 (C-4′), 127.5 (C-12′), 127.4 (phenyl C-8), 125.1 (C-3′), 124.6 (C-7′), 121.3 (phenyl C-7), 115.8 (phenyl C-4a), 112.7 (phenyl C-5), 75.4 (C-2), 51.8 (OCH_3_), 39.6 (2C, C-1′ and C-5′), 38.3 (C-9′), 31.5 (C-3), 27.5 (C-10′), 26.6 (C-6′), 24.2 (C-10), 22.6 (C-2′), 22.3 (C-4), 16.2 (C-9), 16.1 (C4′-*C*H_3_), 15.9 (C8′-CH_3_), 12.5 (C12′-*C*H_3_); HRMS (ESI) *m*/*z* = 463.2819 calcd. for C_28_H_40_O_4_Na [M + Na]^+^; found: 463.2825.

*Methyl 5-[(R)-6-hydroxy-2,8-dimethylchroman-2-yl]pent-2E-enoate* (**12b**), colorless oil; Yield 91%; ^1^H-NMR (CDCl_3_): δ 7.14–6.91 (m, 1H, C3′-H), 6.48 (d, *J* = 2.8 Hz, 1H, phenyl C5-H), 6.38 (d, *J* = 2.8 Hz, 1H, phenyl C7-H), 5.82 (d, *J* = 16.0 Hz, 1H, C4′-H), 4.51 (*br* s, 1H, OH), 3.72 (s, 3H, OCH_3_), 2.75–2.56 (m, 2H, C4-H_2_), 2.46–2.38 (m, 2H, C2′-H_2_), 2.11 (s, 3H, C9-H_3_), 1.86–1.56 (m, 4H, C1′-H_2_ and C3′-H_2_), 1.25 (s, 3H, C10-H_3_); ^13^C-NMR (CDCl_3_): δ 167.3 (C=O), 149.8 (C-3′), 148.1 (phenyl C-6), 145.7 (phenyl C-8a), 127.5 (phenyl C-8), 121.1 (C-4′), 120.9 (phenyl C-7), 115.9 (phenyl C-4a), 112.7 (phenyl C-5), 74.9 (C-2), 51.6 (OCH_3_), 38.1 (C-1′), 31.6 (C-3), 26.6 (C-2′), 23.9 (C-10), 22.4 (C-4), 16.1 (C-9); HRMS (ESI) *m*/*z* = 313.1410 calcd. for C_17_H_22_O_4_Na [M + Na]^+^; found: 313.1416.

*Methyl 9-[(R)-6-hydroxy-2,8-dimethylchroman-2-yl]-6-methylnona-2E,6E-dienoate* (**13b**), colorless oil; Yield 90%; ^1^H-NMR (CDCl_3_): δ 6.99–6.85 (m, 1H, C7′-H), 6.48 (d, *J* = 2.8 Hz, 1H, phenyl C5-H), 6.38 (d, *J* = 2.8 Hz, 1H, phenyl C7-H), 5.82 (d, *J* = 15.6, 1H, H-8′), 5.15 (t, *J* = 7.2 Hz, 1H, H-3′), 4.94 (brs, 1H, OH), 3.72 (s, 3H, OCH_3_), 2.71–2.62 (m, 2H, C4-H_2_), 2.36–2.23 (m, 2H, H-6′), 2.17–2.03 (m, 7H, H-2′, 5′ and 9), 1.82–1.49 (m, 7H, H-1′, 3 and 4′-CH_3_), 1.24 (s, 3H, H-10); ^13^C-NMR (CDCl_3_): δ 167.5 (C=O), 149.6 (C-7′), 148.1 (phenyl C-6), 145.8 (phenyl C-8a), 133.6 (C-4′), 127.3 (phenyl C-8), 125.5 (C-3′), 121.2 (C-8′), 120.9 (phenyl C-7), 115.8 (phenyl C-4a), 112.7 (phenyl C-5), 75.3 (C-2), 51.6 (OCH_3_), 39.6 (C-1′), 37.9 (C-5′), 31.5 (C-3), 30.8 (C-6′), 24.1 (C-10), 22.5 (C-2′), 22.2 (C-4), 16.1 (C-9), 15.9 (C4′-*C*H_3_); HRMS (ESI) *m*/*z* = 381.2036 calcd. for C_22_H_30_O_4_Na [M + Na]^+^; found: 381.2033.

*Methyl 13-[(R)-6-hydroxy-2,8-dimethylchroman-2-yl]-6,10-dimethyltrideca-2E,6E,10E-trienoate* (**14b**), colorless oil; Yield 90%; ^1^H-NMR (CDCl_3_): δ 6.99–6.95 (m, 1H, C11′-H), 6.48 (d, *J* = 2.8 Hz, 1H, phenyl C5-H), 6.38 (d, *J* = 2.8 Hz, 1H, phenyl C7-H), 5.81(d, *J* = 15.6 Hz, 1H, C12′-H), 5.11 (d, *J* = 1.2 Hz, 2H, C3′-H and C7′-H), 4.73 (*br* s, 1H, OH), 3.72 (s, 3H, OCH_3_), 2.66 (t, *J* = 7.2 Hz, 2H, C4-H_2_), 2.31–2.23 (m, 2H, C10′-H_2_), 2.08–1.92 (m, 11H, C9-H_3_, C2′-H_2_, C5′-H_2_, C6′-H_2_ and C9′-H_2_), 1.92–1.51 (m, 10H, C1′-H_2_, C3-H_2_, C4′-CH_3_ and C8′-CH_3_), 1.26 (s, 3H, C10-H_3_); ^13^C-NMR (CDCl_3_): δ 167.4 (C=O), 149.7 (C-11′), 148.0 (phenyl C-6), 146.0 (phenyl C-8a), 134.9 (C-8′), 133.5 (C-4′), 127.4 (phenyl C-8), 125.3 (C-3′), 124.6 (C-7′), 121.3 (C-12′), 120.8 (phenyl C-7), 115.8 (phenyl C-4a), 112.7 (phenyl C-5), 75.4 (C-2), 51.6 (OCH_3_), 39.6 (C-5′), 39.5 (C-1′), 37.9 (C-9′), 31.5 (C-3), 30.9 (C-10′), 26.5 (C-6′), 24.3 (C-10), 22.6 (C-2′), 22.2 (C-4), 16.2 (C-9), 16.0 (C4′-*C*H_3_), 15.9 (C8′-CH_3_); HRMS (ESI) *m*/*z* = 449.2662 calcd. for C_27_H_38_O_4_Na [M + Na]^+^; found: 449.2675.

#### 3.2.5. Synthesis of Methyl 13-[(*R*)-6-methoxy-2,8-dimethylchroman-2-yl]-2,6,10-trimethyltrideca-2*E*,6*E*,10*E*-trienoate (**15**)

To a stirred solution of NaH (4.6 mg, 0.2 mmol, 60%) in anhydrous THF (2 mL) at 0 °C under argon atmosphere was added **14a** (44 mg, 0.1 mmol) and stirred at room temperature for 15 min. The reaction mixture was cooled to 0 °C, methyl iodide (0.13 mL, 0.2 mml) was added dropwise, and stirred at room temperature for 8 h. The reaction mixture was quenched by adding cold water (2 mL) and extracted with ethyl acetate (2 × 5 mL). The combined organic layers were dried over anhydrous Na_2_SO_4_, and solvents were removed under reduced pressure. The residue was purified by silica gel column using hexanes-ethyl acetate as eluents to afford compound **15** (41 mg, yield 90%) as colorless oil; ^1^H-NMR (CDCl_3_): δ 6.73 (t, *J* = 1.6 Hz, 1H, C11′-H), 6.56 (d, *J* = 2.8 Hz, 1H, phenyl C5-H), 6.43 (d, *J* = 2.8 Hz, 1H, phenyl C7-H), 5.13 (t, *J* = 6.4 Hz, 2H, C3′-H and C7′-H), 3.72 (s, 6H, OCH_3_ and COOCH_3_), 2.76–2.70 (m, 2H, C4-H_2_), 2.26–2.21 (m, 2H, C10′-H_2_), 2.17–2.18 (m, 11H, C9-H_3_, C2′-H_2_, C5′-H_2_, C6′-H_2_ and C9′-H_2_), 1.85–1.47 (m, 13H, C3-H_2_, C1′-H_2_, C4′-CH_3_, C8′-CH_3_ and C12′-CH_3_), 1.25 (s, 3H, C10-H_3_); ^13^C-NMR (CDCl_3_): δ 168.8 (C=O), 152.2 (phenyl C-6), 146.2 (phenyl C-8a), 142.4 (C-11′), 135.1 (C-8′), 134.0 (C-4′), 127.5 (C-12′), 127.3 (phenyl C-8), 125.2 (C-3′), 124.5 (C-7′), 121.0 (phenyl C-7), 114.9 (phenyl C-4a), 111.1 (phenyl C-5), 75.4 (C-2), 55.7 (OCH_3_), 51.8 (COOCH*_3_*), 39.8 (C-5′), 39.7 (C-1′), 38.3 (C-9′), 31.5 (C-3), 27.5 (C-10′), 26.7 (C-6′), 22.8 (C-4), 22.3 (C-2′), 16.3 (C-9), 16.1 (C4′-*C*H_3_), 16.0 (C8′-CH_3_), 12.5 (C12′-*C*H_3_); HRMS (ESI) *m*/*z* = 477.2975 calcd. for C_29_H_42_O_4_Na [M + Na]^+^; found: 477.2995.

#### 3.2.6. Synthesis of 13-[(*R*)-6-methoxy-2,8-dimethylchroman-2-yl]-2,6,10-trimethyltrideca-2*E*,6*E*,10*E*-trienoic Acid (**16**)

To a stirred solution of compound **15** (27 mg, 0.06 mmol) in THF-MeOH-H2O (3:1:1, 4 mL) was added LiOH (7 mg, 0.3 mmol). The reaction mixture was heated at 40 °C under N_2_ for 12 h, then cooled to room temperature, diluted with ethyl acetate (10 mL), and acidified with 1N HCl (5 mL). The aqueous layer was extracted with ethyl acetate (2 × 15 mL). The combined organic layer was washed with brine (15 mL) and dried over anhydrous Na_2_SO_4_. Solvents were removed under reduced pressure and the residue was purified by silica gel column using hexanes-ethyl acetate (6:4) as eluents to afford compound **16** (23 mg, yield 88%) as colorless oil; ^1^H-NMR (CDCl_3_): δ 6.87 (t, *J* = 1.2 Hz, 1H, C11′-H), 6.56 (d, *J* = 2.8 Hz, 1H, phenyl C5-H), 6.43 (d, *J* = 2.8 Hz, 1H, phenyl C7-H), 5.13 (t, *J* = 6.4 Hz, 2H, C3′-H and C7′-H), 3.72 (s, 3H, OCH_3_), 2.72 (t, *J* = 6.0 Hz, 2H, C4-H_2_), 2.35–2.21 (m, 2H, C10′-H_2_), 2.19–2.10 (m, 9H, C5′-H_2_, C6′-H_2_, C9′-H_2_ and C9-H_3_), 2.01–1.93 (m, 2H, C2′-H_2_), 1.83–1.45 (m, 13H, C1′-H_2_, C3-H_2_, C4′-CH_3_, C8′-CH_3_ and C12′-CH_3_), 1.26 (s, 3H, C10-H_3_); ^13^C-NMR (CDCl_3_): δ 172.8 (COOH), 152.2 (C-11′), 146.2 (phenyl C-6), 145.0 (phenyl C-8a), 135.0 (C-8′), 133.8 (C-4′), 127.3 (C-12′), 126.9 (phenyl C-8), 125.3 (C-3′), 124.5 (C-7′), 121.0 (phenyl C-4a), 114.9 (phenyl C-7), 111.1 (phenyl C-5), 75.4 (C-2), 55.7 (OCH_3_), 39.8 (C-5′), 39.6 (C-1′), 38.1 (C-9′), 31.5 (C-3), 27.6 (C-6′), 26.6 (C-10′), 24.1 (C-10), 22.8 (C-4), 22.3 (C-2′), 16.3 (C-9), 16.1 (C8′-*C*H_3_), 16.0 (C4′-*C*H_3_), 12.1 (C12′-*C*H_3_); MS (ESI) *m*/*z* 439.2 [M − H]^−^.

#### 3.2.7. Synthesis of (*R*)-2,8-dimethyl-2-[(3*E*,7*E*)-4,8,12-trimethyltrideca-3,7,11-trien-1-yl]chroman-6-yl Acetate (**17**)

To a solution of δ-tocotrienol (**3**) (2 g, 5 mmol) in CH_2_Cl_2_ (25 mL) at 0 °C was added triethylamine (1.35 mL, 10 mmol) and DMAP (100 mg). The resulting mixture was stirred at room temperature for 15 min, and cooled to 0 °C. At the same temperature Ac_2_O (0.71 mL, 7.5 mmol) was added and continue stirring at room temperature for 5 h. The reaction mixture was partitioned between CH_2_Cl_2_ (25 mL) and water (50 mL). The aqueous layer was extracted with CH_2_Cl_2_ (2 × 25 mL), and the combined organic layers were washed with water (50 mL) and brine (50 mL), dried over anhydrous Na_2_SO_4_, filtered, and concentrated under reduced pressure. The crude product was chromatographed on silica (hexanes-ethyl acetate 9:1) to afford compound **17** (1.97 g, 90%) as colorless oil: ^1^H-NMR (CDCl_3_): *δ* 6.67 (d, *J* = 2.4 Hz, 1H, phenyl C5-H), 6.61 (d, *J* = 2.4 Hz, 1H, phenyl C7-H), 5.17–5.05 (m, 3H, C3′-H, C7′-H and C11′-H), 2.74–2.68 (m, 2H, C4-H_2_), 2.24 (s, 3H, COCH_3_), 2.16–1.92 (m, 13H, C9-H_3_, C2′-H_2_, C5′-H_2_, C6′-H_2_, C9′-H_2_ and C10′-H_2_), 1.83–1.54 (m, 16H, C3-H_2_, C1′-H_2_, C4′-CH_3_, C8′-CH_3_, C12′-CH_3_ and C13′-H_3_), 1.27 (s, 3H, C10-H_3_); ^13^C-NMR (CDCl_3_): δ 170.3 (*C*OCH_3_), 149.8 (phenyl C-6), 142.6 (phenyl C-8a), 135.3 (C-8′), 135.0 (C-4′), 131.3 (C-12′), 127.4 (phenyl C-8), 124.5 (C-3′), 124.3 (C-7′), 124.2 (C-11′), 121.2 (phenyl C-7), 120.9 (phenyl C-4a), 119.1 (phenyl C-5), 75.9 (C-2), 40.0 (C-1′), 39.8 (2C, C-5′ and C-9′), 31.0 (C-3), 26.8 (C-10′), 26.7 (C-8′), 25.8 (C-13′), 24.2 (C-10), 22.5 (C-2′), 22.2 (C-4), 21.2 (CO*C*H_3_), 17.7 (C12′-*C*H_3_), 16.2 (C-9), 16.1 (C8′-*C*H_3_), 16.0 (C4′-*C*H_3_); GC-MS (EI) *m*/*z* 438.3 (M^+^, 100), 423.2, 219.1, 177.1, 69.1. 

#### 3.2.8. Synthesis of Compounds **19** and **20**

To a stirred solution of compound **17** (613 mg, 1.4 mmol) in anhydrous CH_2_Cl_2_ (12 mL) was added PhSeCl (27 mg, 0.14 mmol) under an Argon atmosphere. To this solution NCS (205 mg, 1.54 mmol) was added and resulting mixture was stirred for 4.5 h (monitored by TLC). Solvent was removed under reduced pressure, and to the residue was added Et_2_O (15 mL). The ether layer was decanted from the solid, and the organic layer was washed with H_2_O (2 × 10 mL), brine (10 mL), dried over anhydrous Na_2_SO_4_, filtered, and concentrated under reduced pressure. The resulting crude was dissolved in acetone-H_2_O (1:1, 20 mL), followed by adding 2,4,6-collidine (0.65 mL, 4.9 mmol) and AgBF_4_ (681 mg, 3.5 mmol). The resulting mixture was heated at 70 °C for 6 h (monitored by TLC), then cooled to room temperature and filtered through a pad of celite. Acetone was removed from the filtrate under reduced pressure and the residue was extracted with ethyl acetate (3 × 15 mL). The combined organic layers were washed with 2 M HCl (3 × 10 mL) and brine (15 mL), dried over anhydrous Na_2_SO_4_, filtered, and concentrated under reduced pressure. The residue was purified by column chromatography (hexane-ethyl acetate, 7:3) on silica gel to afford **19** and **20**.

*(2R)-2-[(3E,7E)-11-Hydroxy-4,8,12-trimethyltrideca-3,7,12-trien-1-yl]-2,8-dimethylchroman-6-yl acetate* (**19**), 165 mg, colorless oil; Yield 26%; ^1^H-NMR (CDCl_3_): δ 6.66 (d, *J* = 2.4 Hz, 1H, phenyl C5-H), 6.61 (d, *J* = 2.4 Hz, 1H, phenyl C7-H), 5.15–5.05 (m, 2H, C3′-H and C7′-H), 4.92 (s, 1H, C13′-H), 4.82 (s, 1H, C13′-H), 4.04 (t, *J* = 6.4 Hz, 1H, C11′-H), 2.73 (t, *J* = 3.2 Hz, 2H, C4-H_2_), 2.24 (s, 3H, COCH*_3_*), 2.17–1.91 (m, 11H, C9-H_3_, C2′-H_2_, C5′-H_2_, C6′-H_2_ and C9′-H_2_), 1.82–1.45 (m, 15H, C3-H_2_, C1′-H_2_, C10′-H_2_, C4′-CH_3_, C8′-CH_3_ and 12′-CH_3_), 1.27 (s, 3H, C10-H_3_); ^13^C-NMR (CDCl_3_): δ 170.3 (*C*OCH_3_), 149.8 (phenyl C-6), 147.5 (C-12′), 142.5 (phenyl C-8a), 135.1 (C-4′), 134.8 (C-8′), 127.4 (phenyl C-8), 124.7 (C-7′), 124.3 (C-3′), 121.2 (phenyl C-7), 120.9 (phenyl C-4a), 119.1 (phenyl C-5), 111.0 (C-13′), 75.9 (C-2), 75.6 (C-11′), 39.9 (C-5′), 39.6 (C-1′), 35.7 (C-9′), 33.2 (C-10′), 31.0 (C-3), 26.5 (C-6′), 24.2 (C-10), 22.5 (C-4), 22.2 (C-2′), 21.1 (CO*C*H_3_), 17.7 (C12′-*C*H_3_), 16.2 (C-9), 16.0 (C8′-*C*H_3_), 15.9 (C4′-*C*H_3_); GC-MS (EI) *m*/*z* 454.3 (M^+^), 439.2, 412.3, 219.1, 177.1, 137.1 (100), 93.1, 55.1. 

*(R)-2-[(3E,7E,11E)-13-Hydroxy-4,8,12-trimethyltrideca-3,7,11-trien-1-yl]-2,8-dimethylchroman-6-yl acetate* (**20**), 204 mg, colorless oil; Yield 32%; ^1^H-NMR (CDCl_3_): δ 6.66 (d, *J* = 2.4 Hz, 1H, phenyl C5-H), 6.61 (d, *J* = 2.4 Hz, 1H, phenyl C7-H), 5.42–5.37 (m, 1H, C11′-H), 5.17–5.05 (m, 2H, C3′-H and C7′-H), 3.97 (s, 2H, C13′-H_2_), 2.72–2.70 (m, 2H, C4-H_2_), 2.24 (s, 3H, CO*C*H*_3_*), 2.16–1.95 (m, 13H, C9-H_3_, C2-H_2_, C5′-H_2_, C6′-H_2_, C9′-H_2_ and C10′-H_2_), 1.74–1.54 (m, 13H, C1′-H_2_, C3-H_2_, C4′-CH_3_, C8′-CH_3_ and C12′-CH_3_), 1.27 (s, 3H, C10-H_3_); ^13^C-NMR (CDCl_3_): δ 170.3 (*C*OCH_3_), 149.8 (phenyl C-6), 142.6 (phenyl C-8a), 135.2 (C-12′), 134.7 (C-8′), 133.7 (C-4′), 127.4 (phenyl C-8), 126.2 (C-11′), 124.5 (C-7′), 124.3 (C-3′), 121.2 (phenyl C-7), 120.9 (phenyl C-4a), 119.1 (phenyl C-5), 75.9 (C-2), 69.1 (C-13′), 40.0 (C-5′), 39.7 (C-1′), 39.4 (C-9′), 31.0 (C-3), 26.6 (C-6′), 26.3 (C-10′), 24.2 (C-10), 22.5 (C-4), 22.2 (C-2′), 21.1 (CO*C*H_3_), 16.2 (C-9), 16.1 (C8′-*C*H_3_), 16.0 (C4′-*C*H_3_), 13.7 (C12′-*C*H_3_); MS (ESI) *m*/*z* 453.2 [M − H]^−^.

#### 3.2.9. Conversion of **19** to **20**

To a solution of **19** (228 mg, 0.5 mmol) in pyridine (5 mL) at 0 °C was added DMAP (10 mg). After 10 min, MsCl (0.08 mL, 3.0 mmol) was added. The reaction mixture was stirred for 1 h and then quenched with sat. NaHCO_3_ solution (15 mL) and extracted with ethyl acetate (2 × 20 mL). The combined organic layers were washed with 2 M HCl (15 mL), sat. NaHCO_3_ solution (15 mL), and brine (15 mL), dried over anhydrous Na_2_SO_4_, and concentrated under reduced pressure. The residue was dissolved in acetone (5 mL) and H_2_O (2 mL), NaOAc (410 mg in 1 mL water) was added, and the mixture was heated at reflux for 2 h. Acetone was removed under reduced pressure and the resulting crude was extracted with ethyl acetate (2 × 10 mL), dried over anhydrous Na_2_SO_4_, and concentrated under reduced pressure. The residue was purified by column chromatography (hexane-ethylacetate, 7:3) on silica gel to afford mixture of **20** (78 mg, yield 34%) and **19** (73 mg, yield 32%).

#### 3.2.10. Synthesis of (*R*)-2,8-dimethyl-2-[(3*E*,7*E*,11*E*)-4,8,12-trimethyl-13-oxotrideca-3,7,11-trien-1-yl]chroman-6-yl Acetate (**21**)

To a stirred solution of **20** (45 mg, 0.1 mmol) in CH_2_Cl_2_ (3 mL) at 0 °C was added Dess-Martin reagent (51 mg, 0.12 mmol). The reaction was continued at room temperature for another 2 h, and then quenched with saturated aqueous Na_2_S_2_O_3_ solution (3 mL) and saturated aqueous NaHCO_3_ solution (3 mL). Stirred for another 20 min and extracted with CH_2_Cl_2_ (2 × 50 mL). The combined organic layers were dried over anhydrous Na_2_SO_4_, filtered, and concentrated under reduced pressure. The resulting crude residue was purified by column chromatography (hexane-ethyl acetate 8:2) on silica gel to afford **21** (41 mg, yield 92%) as colorless oil; ^1^H-NMR (CDCl_3_): δ 9.37 (s, 1H, aldehyde), 6.66 (d, *J* = 2.4 Hz, 1H, phenyl C5-H), 6.60 (d, *J* = 2.4 Hz, 1H, phenyl C7-H), 6.52–6.41 (m, 1H, C11′-H), 5.16–5.12 (m, 2H, C3′-H and C7′-H), 2.79–2.69 (m, 2H, C4-H_2_), 2.49–2.36 (m, 2H, C10′-H_2_), 2.24 (s, 3H, COCH*_3_*), 2.12–1.91 (m, 11H, C9-H_3_, C2′-H_2_, C5′-H_2_, C6′-H_2_ and C9′-H_2_), 1.86–1.52 (m, 13H, C1′-H_2_, C3-H_2_, C4′-CH_3_, C8′-CH_3_ and C12′-CH_3_), 1.27 (s, 3H, C10-H_3_); ^13^C-NMR (CDCl_3_): δ 195.3 (aldehyde), 170.3 (*C*OCH_3_), 154.5 (C-11′), 149.7 (phenyl C-6), 142.5 (phenyl C-8a), 139.3 (C-12′), 135.0 (C-8′), 133.4 (C-4′), 127.3 (phenyl C-8), 125.6 (C-3′), 124.4 (C-7′), 121.2 (phenyl C-7), 120.9 (phenyl C-4a), 119.1 (phenyl C-5), 75.9 (C-2), 39.9 (C-5′), 39.6 (C-1′), 38.0 (C-9′), 31.0 (C-3), 27.5 (C-6′), 26.6 (C-10′), 24.2 (C-10), 22.4 (C-4), 22.2 (C-2′), 21.1 (CO*C*H_3_), 16.2 (C-9), 16.0 (C8′-*C*H_3_), 15.9 (C4′-*C*H_3_), 9.29 (C12′-*C*H_3_); MS (ESI) *m*/*z* 453.2 [M + H]^+^.

#### 3.2.11. Synthesis of 13-[(*R*)-6-acetoxy-2,8-dimethylchroman-2-yl]-2,6,10-trimethyltrideca-2*E*,6*E*,10*E*-trienoic Acid (**22**)

To a solution of aldehyde **21** (23 mg, 0.05 mmol) in *t*-BuOH (3.0 mL) was added NaH_2_PO_4_ (29 mg, 0.48 mmol) and 2-methyl-2-butene (0.5 mL) successively. The reaction mixture was cooled to 0 °C. A freshly prepared solution of sodium chlorite (47 mg, 0.52 mmol in 0.5 mL H_2_O) was added to the mixture. The resulting mixture was stirred for 2 h at room temperature (monitored by TLC). Water (1.0 mL) was added, and the aqueous layer was extracted with ethyl acetate (3 × 5 mL). The combined organic layers were washed with brine and dried over anhydrous Na_2_SO4. The solvent was removed under reduced pressure, the residue was purified by silica gel column with hexanes-ethyl acetate (1:1) to give **22** (19 mg, yield 80%) as colorless oil. ^1^H-NMR (CDCl_3_): δ 6.87 (t, *J* = 1.2 Hz, 1H, C11′-H), 6.66 (d, *J* = 2.4 Hz, 1H, phenyl C5-H), 6.61 d, *J* = 2.4 Hz, 1H, phenyl C5-H), 5.13 (t, *J* = 6.0 Hz, 2H, C3′-H and C7′-H), 2.72 (t, *J* = 3.6 Hz, 2H, C4-H_2_), 2.36–2.24 (m, 5H, C10′-H_2_ and COCH_3_), 2.09–1.91 (m, 11H, C9-H_3_, C2′-H_2_, C5′-H_2_, C6′-H_2_, and C9′-H_2_), 1.86–1.56 (m, 13H, C1′-H_2_, C3-H_2_, C4′-CH_3_, C8′-CH_3_ and C12′-CH_3_), 1.27 (s, 3H, C10-H_3_); ^13^C-NMR (CDCl_3_): δ 172.9 (C13-C=O), 170.4 (*C*OCH_3_), 149.8 (phenyl C-6), 145.0 (C-11′), 142.6 (phenyl C-8a), 135.1 (C-8′), 133.8 (C-4′), 127.4 (C-12′), 127.0 (phenyl C-8), 125.3 (C-3′), 124.4 (C-7′), 121.2 (2C, phenyl C-4a and C-7), 119.2 (phenyl C-5), 76.0 (C-2), 40.0 (C-5′), 39.6 (C-1′), 38.1 (C-9′), 31.1 (C-3), 27.6 (C-6′), 26.6 (C-10′), 24.2 (C-10), 22.5 (C-4), 22.2 (C-2′), 21.2 (CO*C*H_3_), 16.2 (C-9), 16.1 (C8′-*C*H_3_), 16.0 (C4′-*C*H_3_), 12.1 (C12′-*C*H_3_). MS (ESI) *m*/*z* 467.2 [M − H]^−^.

#### 3.2.12. Synthesis of 13-[(*R*)-6-Hydroxy-2,8-dimethylchroman-2-yl]-2,6,10-trimethyltrideca-2*E*,6*E*,10*E*-trienoic acid (**1**, garcinoic acid)

To a stirred solution of **22** (23 mg, 0.05 mmol) in MeOH (2 mL) was added K_2_CO_3_ (21 mg, 0.15 mmol). Reaction was continued at room temperature under N_2_ until TLC showed complete conversion. The mixture was filtered and the solids was rinsed with ethyl acetate (5 mL) and acidified by adding 1 N HCl (3 mL). The aqueous layer was extracted with ethyl acetate (2 × 10 mL). The combined organic layers were washed with brine (10 mL), dried over anhydrous Na_2_SO_4_, and concentrated under reduced pressure. The residue was purified by silica gel column using hexanes-ethyl acetate (1:1) to afford compound **1** (14 mg, yield 85%) as a colorless oil.

### 3.3. Biochemistry

A fluorescence-based assay, as described previously [21], was used to determine the IC_50_ values for garcinoic acid and its homologs for human DNA polymerase β. DNA substrates were prepared as we reported previously [22]. Briefly, a reporter strand conjugated with 5-carboxytetramethylrhodamine (TAMRA) and an unlabeled primer were annealed to a Black Hole Quencher (BHQ)-labelled template strand in a solution containing 10 mM Tris (pH 8.0) and 50 mM NaCl. The oligonucleotides were annealed in a 1:1.5:1.5 ratio for reporter, primer and template strand, respectively. The oligonucleotides were mixed and incubated for 3 min at 95 °C before cooling to room temperature. 

All the compounds were dissolved in DMSO and the IC_50_ values were determined using a range from 1 μM to 120 μM. The polymerase reaction was performed with 1 nM hpol β, 50 nM DNA, 100 μM dTTP and 2 mM MgCl_2_ in 50 mM Tris (pH 8.0) buffer containing 40 mM NaCl, 2 mM DTT and 0.01% (*w*/*v*) Tween 20. The reaction mix containing enzyme and dTTP were incubated for 5 min with the compound or DMSO in a 96-well plate before adding DNA to initiate the reaction. Polymerase reactions were performed at room temperature (25 °C). The plate was read continuously for one hour with a Synergy H4 plate reader (BioTek, *λ*_ex_ = 525 nm, *λ*_em_ = 598 nm) (Winooski, VT, USA) after initiation of the reaction. The percent inhibition was calculated by dividing the rate of product formation at each compound concentration by the rate of product formation in DMSO control. The data was fit to a four-parameter logistic model with variable slope in Prism (San Diego, CA, USA) to calculate the IC_50_ values. The reactions were performed in triplicate and the mean IC_50_ value is reported with 95% confidence intervals.

## 4. Conclusions

In summary, using natural product DT3 as a starting material, we have developed a highly efficient method for the synthesis of garcinoic acid and its analogs and established a preliminary SAR for their inhibition of hpol β. Our SAR study revealed that the side chain length and the terminal carboxylic acid group of garcinoic acid are critical for inhibiting hpol β, whereas the phenolic OH group on the chromane ring can tolerate modification. In addition, we described a novel, facile semi-synthesis of garcinoic acid from DT3, which is more efficient than the previously report procedure. Our SAR study together with the new synthetic methods offered opportunities to further design and synthesize garcinoic acid analogs as hpol β inhibitors.

## Figures and Tables

**Table 1:** Screening of reaction conditions for cross metathesis between **4** and **5**.

Entry	Catalyst *^a^*	Substrate	Solvent	Temp	Time	Yield % (6/7/8) *^b^*
1	Grubbs 1st generation	**5b**	CH_2_Cl_2_	rt	24 h	0
2	Grubbs 1st generation	**5b**	CH_2_Cl_2_	reflux	24 h	0
3	Grubbs 1st generation	**5b**	1,2-DCE	reflux	24 h	0
4	Grubbs 1st generation	**5b**	toluene	reflux	24 h	0
5	Grubbs 2nd generation	**5b**	CH_2_Cl_2_	reflux	24 h	Trace
6	Grubbs 2nd generation	**5b**	toluene	reflux	3 h	54/14/8
7	Grubbs 2nd generation	**5b**	toluene	reflux	24 h	62/15/trace
8	Grubbs 2nd generation	**5b**	neat	80 °C	24 h	Trace
9	Grubbs 2nd generation	**5a**	CH_2_Cl_2_	rt	24 h	0
10	Grubbs 2nd generation	**5a**	CH_2_Cl_2_	reflux	24 h	0
11	Grubbs 2nd generation	**5a**	1,2-DCE	reflux	24 h	Trace
12	Grubbs 2nd generation	**5a**	toluene	reflux	24 h	17/15/11

*^a^*5 mol % of the catalyst was used; *^b^*Isolated yield of products **6**, **7**, and **8**.

**Table 2:** IC_50_ values of garcinoic acid (**1**) and its analogs in inhibiting hpol β.

Compd.	Structure	IC_50_ (µM)	Compd.	Structure	IC_50_ (µM)
**1**	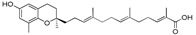	11	**12b**	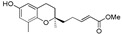	>120
**9a**	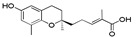	>120	**13a**	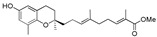	>120
**9b**	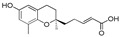	>120	**13b**	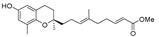	>120
**10a**	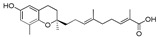	52	**14a**	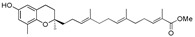	>120
**10b**	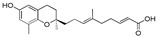	49	**14b**	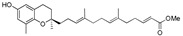	>120
**11**	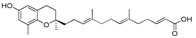	23	**15**	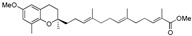	>120
**12a**	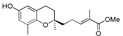	>120	**16**	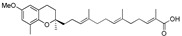	31

## Supplementary Materials

The following are available online. Figure S1: IC_50_ determination of garcinoic acid (**1**), **10a**, and **9a** against hpol β; copies of ^1^H-NMR and ^13^C-NMR spectra of compounds **4**, **6a**–**8a**, **6b**–**8b**, **9a**, **10a**, **1**, **9b**, **10b**, **11**, **12a**–**14a**, **12b**–**14b**, **15**–**17**, and **19**–**22**.
