# Supplementary material for: A Facile Semisynthesis and Evaluation of Garcinoic Acid and Its Analogs for the Inhibition of Human DNA Polymerase β
Source: Molecules. 2020 Dec 11;25(24):5847. doi: 10.3390/molecules25245847 (PMC7763917; doi:10.3390/molecules25245847)

molecules

Supporting Information

Facile Semisynthesis and Evaluation of Garcinoic Acid and Its Analogs for the Inhibition of Human DNA Polymerase β

Satheesh Gujarathi [1], Maroof Khan Zafar [2], Xingui Liu [1], Robert L. Eoff [2] and Guangrong Zheng [1,3,*]

[1] Department of Pharmaceutical Sciences, College of Pharmacy, University of Arkansas for Medical Sciences, Little Rock, AR 72205, USA
[2] Department of Biochemistry and Molecular Biology, College of Medicine, University of Arkansas for Medical Sciences, Little Rock, AR 72205, USA
[3] Department of Medicinal Chemistry, College of Pharmacy, University of Florida, Gainesville, FL 32610, USA

Table of Contents

Figure S1……………………………………………………………………………………S-2

Copies of ^{1}H & ^{13}C NMR Spectra………………………………………………………… S-3

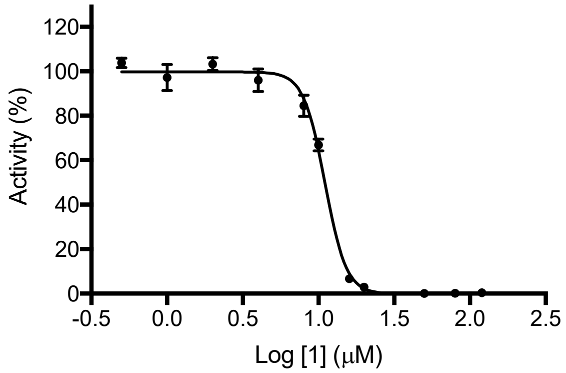

IC_{50} = 11 μM

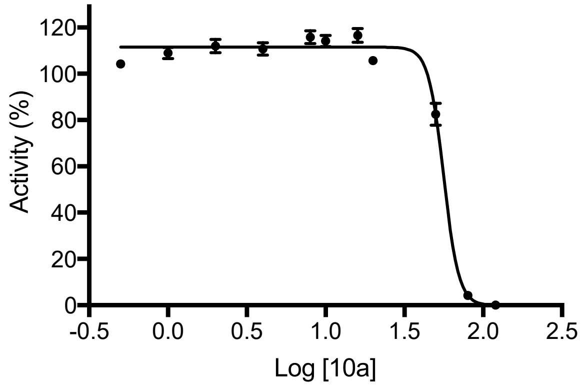

IC_{50} = 52 μM

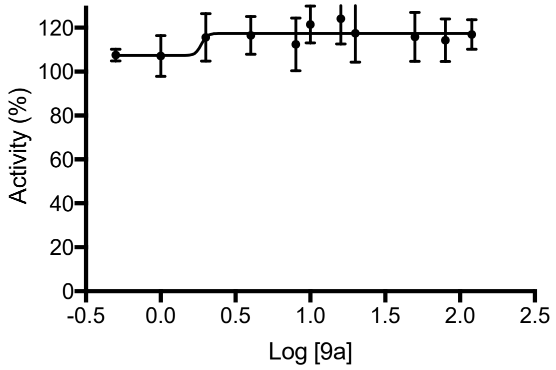

IC_{50} = >120 μM

Figure S1. IC_{50} determination of garcinoic acid (**1**), **10a**, and **9a** against hpol *β*.

^{1}H and ^{13}C NMR spectra of 4

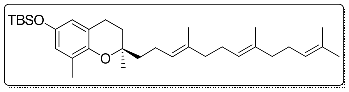

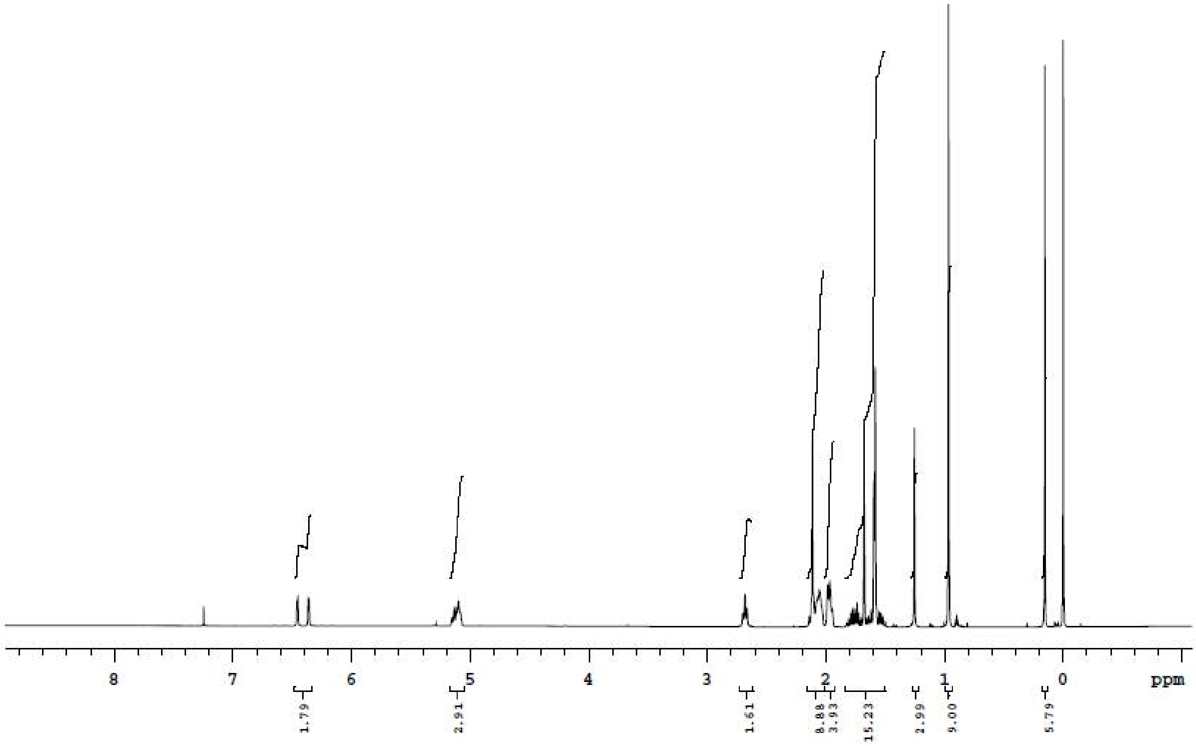

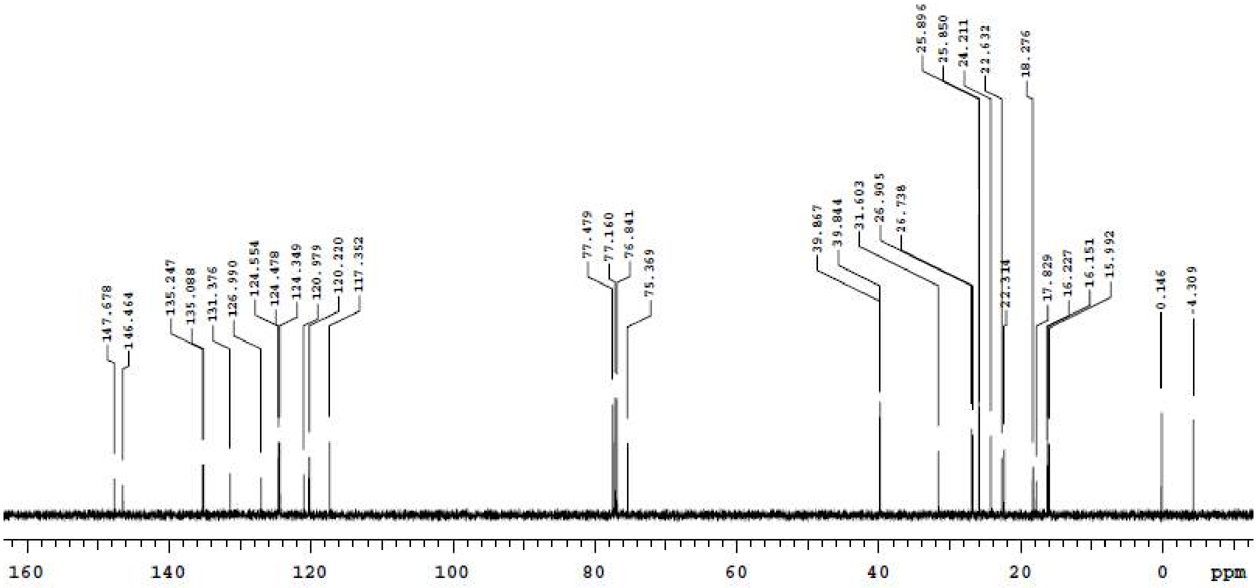

^{1}H and ^{13}C NMR spectra of 6a

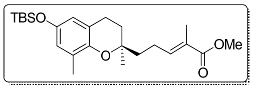

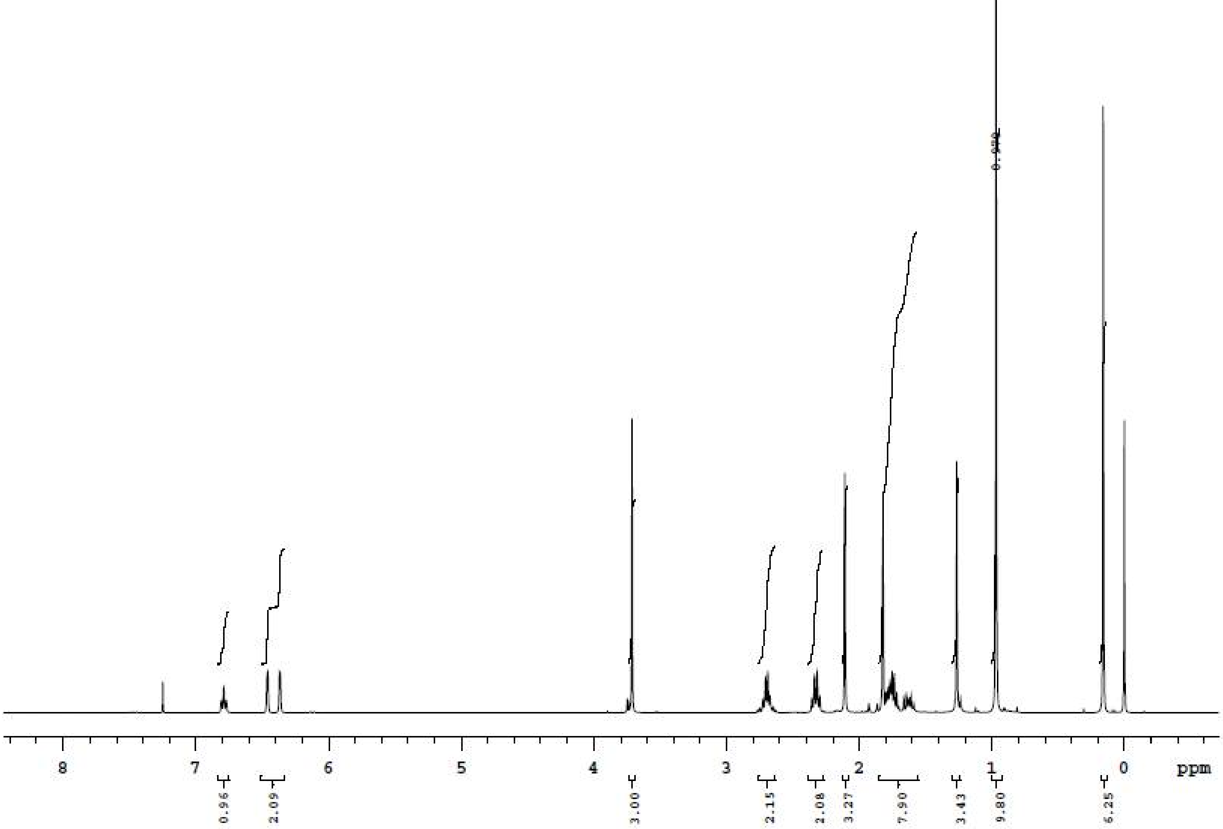

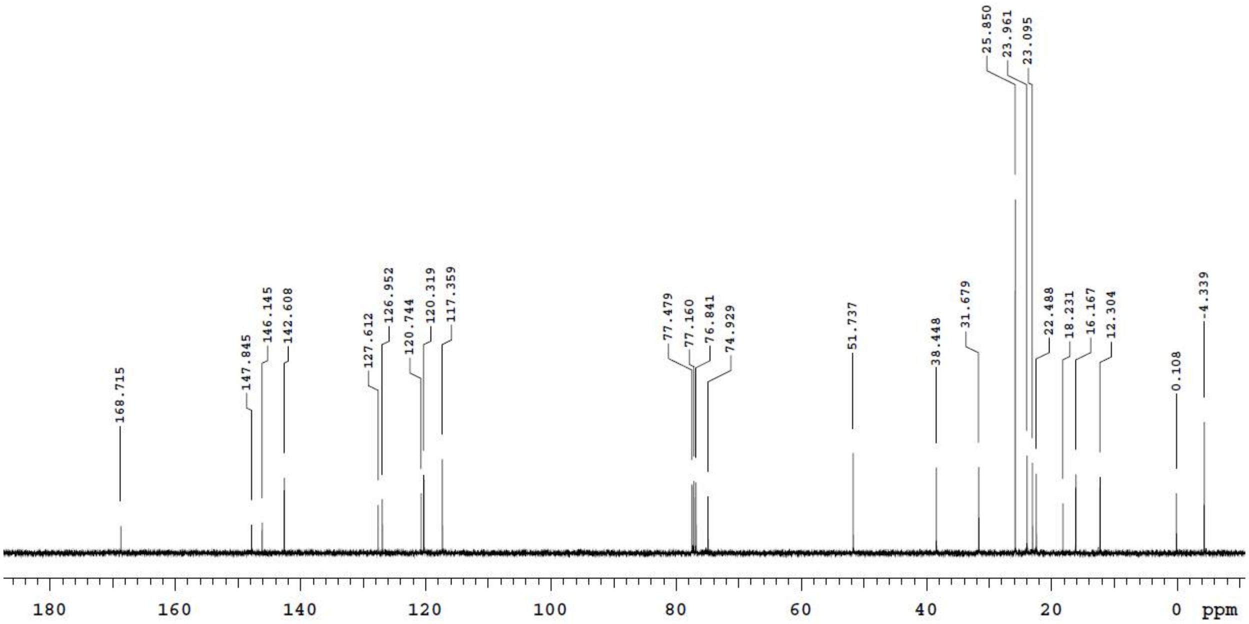

^{1}H and ^{13}C NMR spectra of 7a

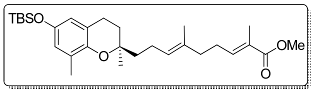

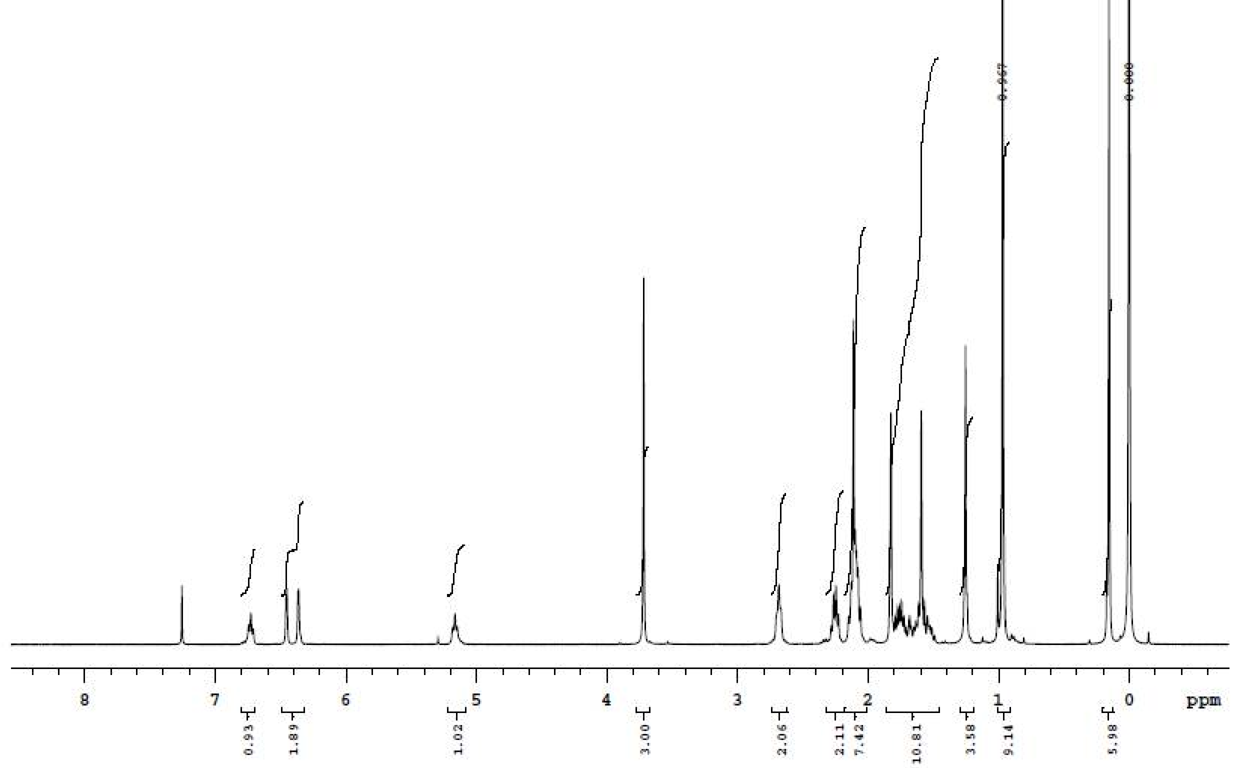

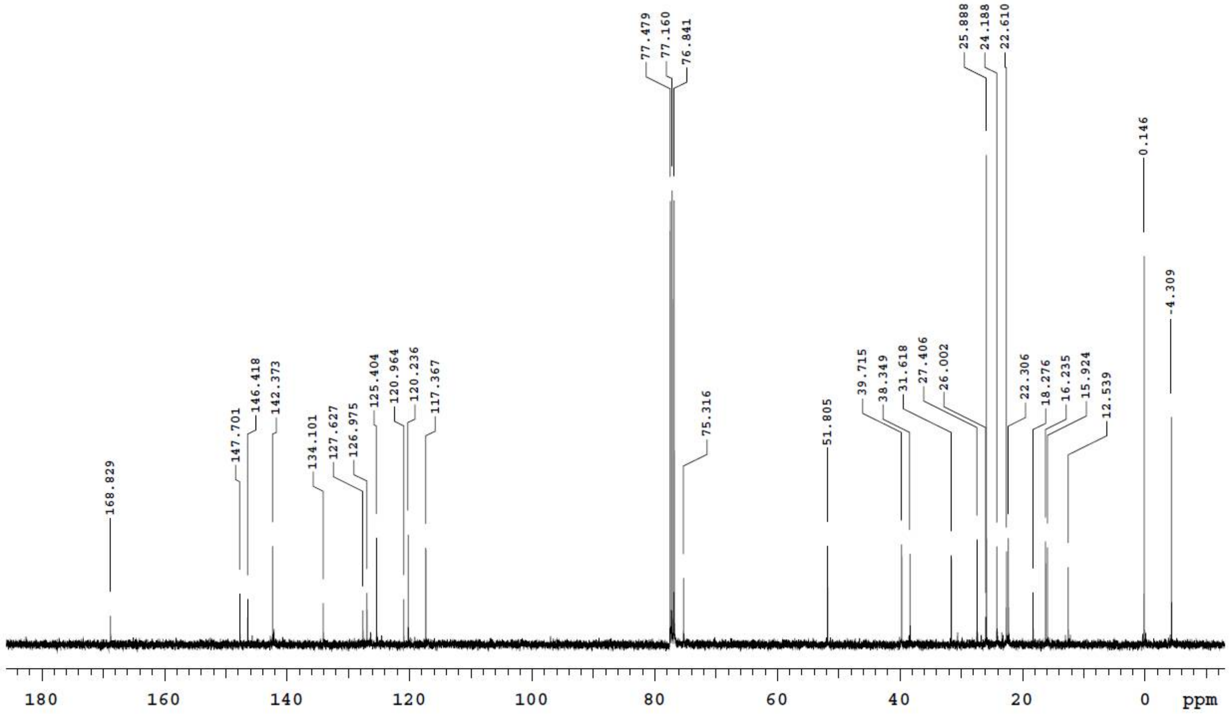

^{1}H and ^{13}C NMR spectra of 8a

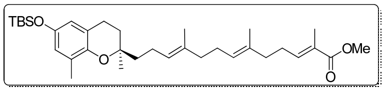

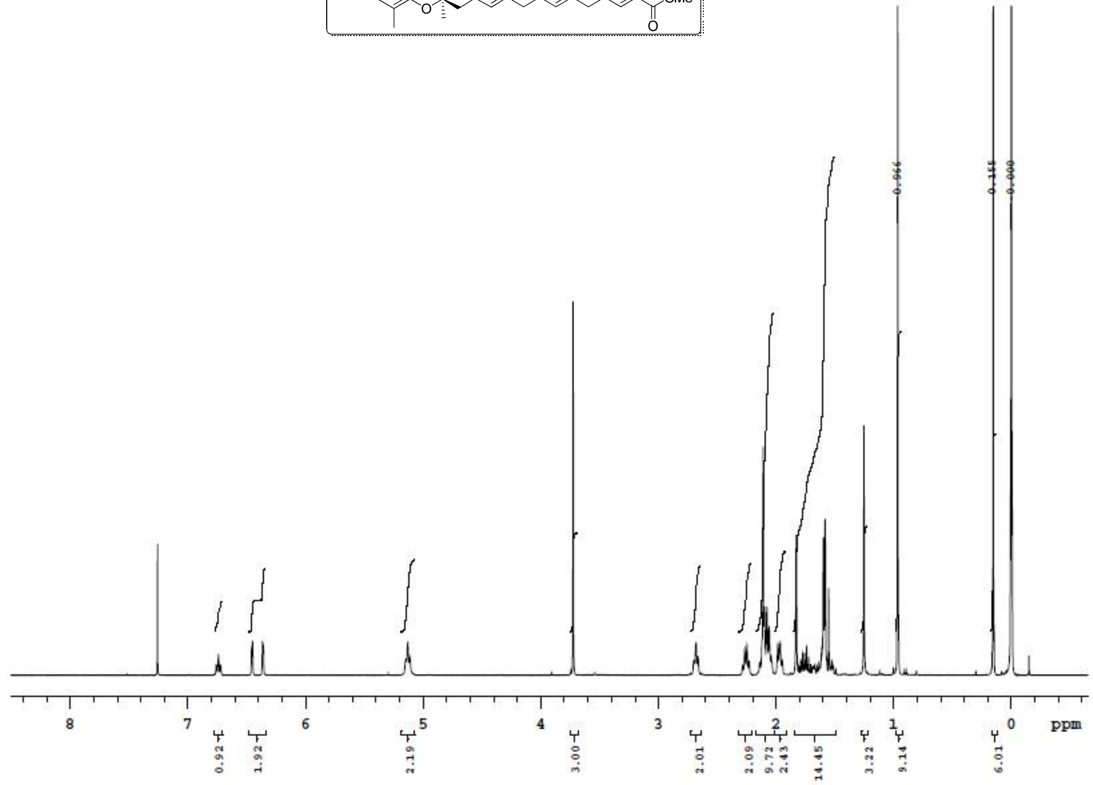

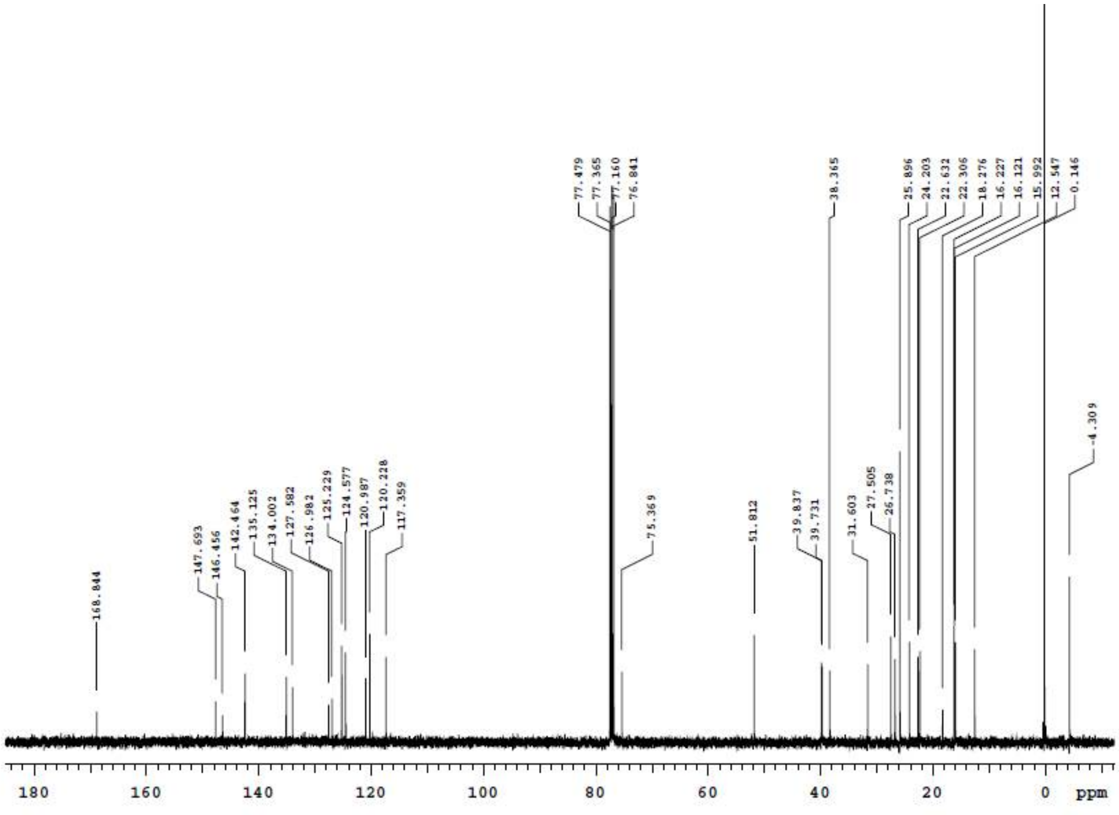

^{1}H and ^{13}C NMR spectra of 6b

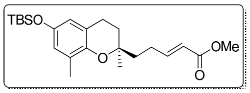

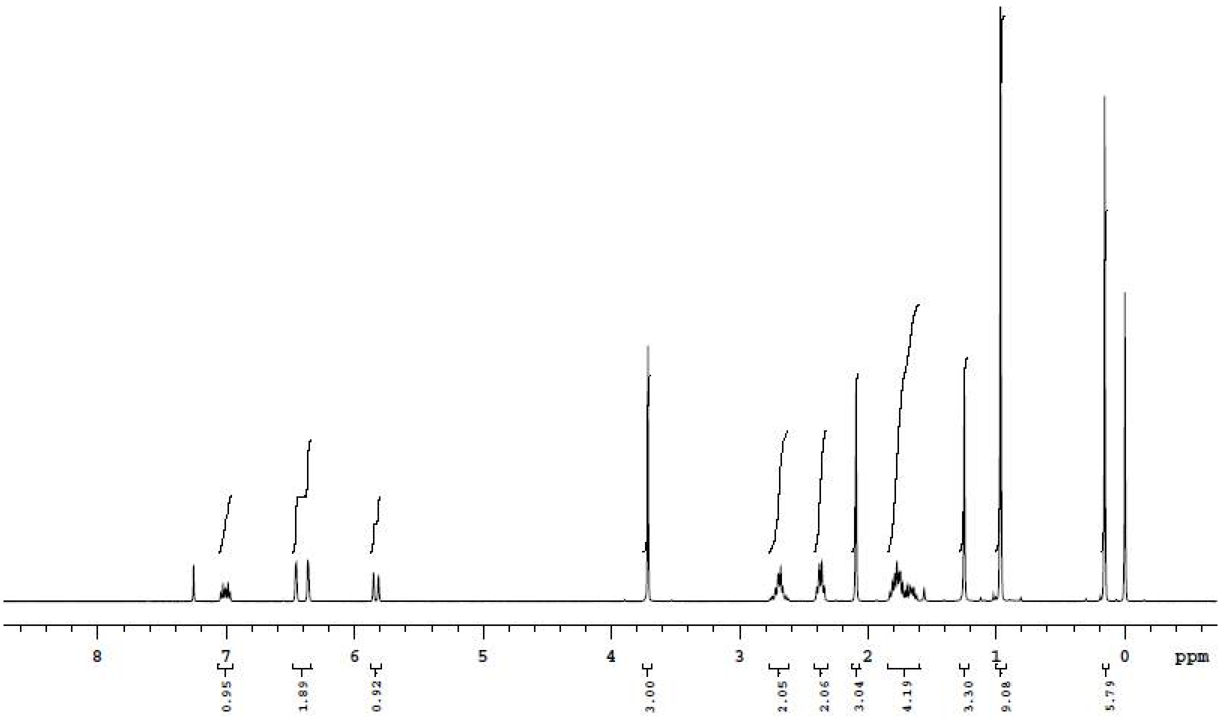

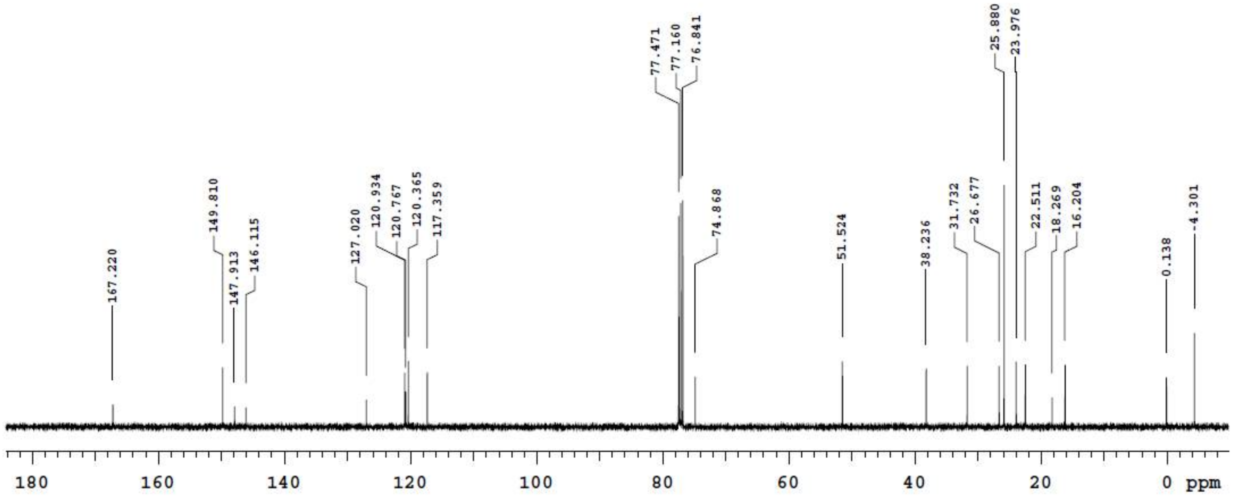

^{1}H and ^{13}C NMR spectra of 7b

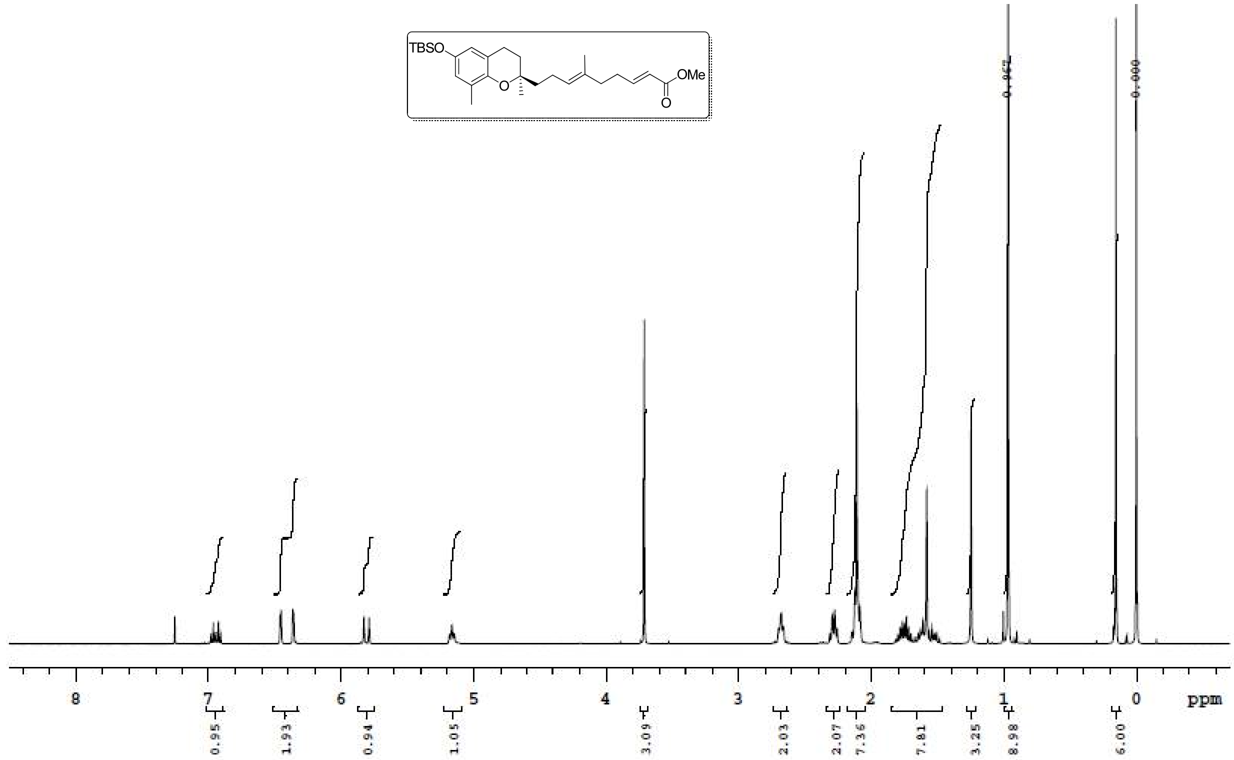

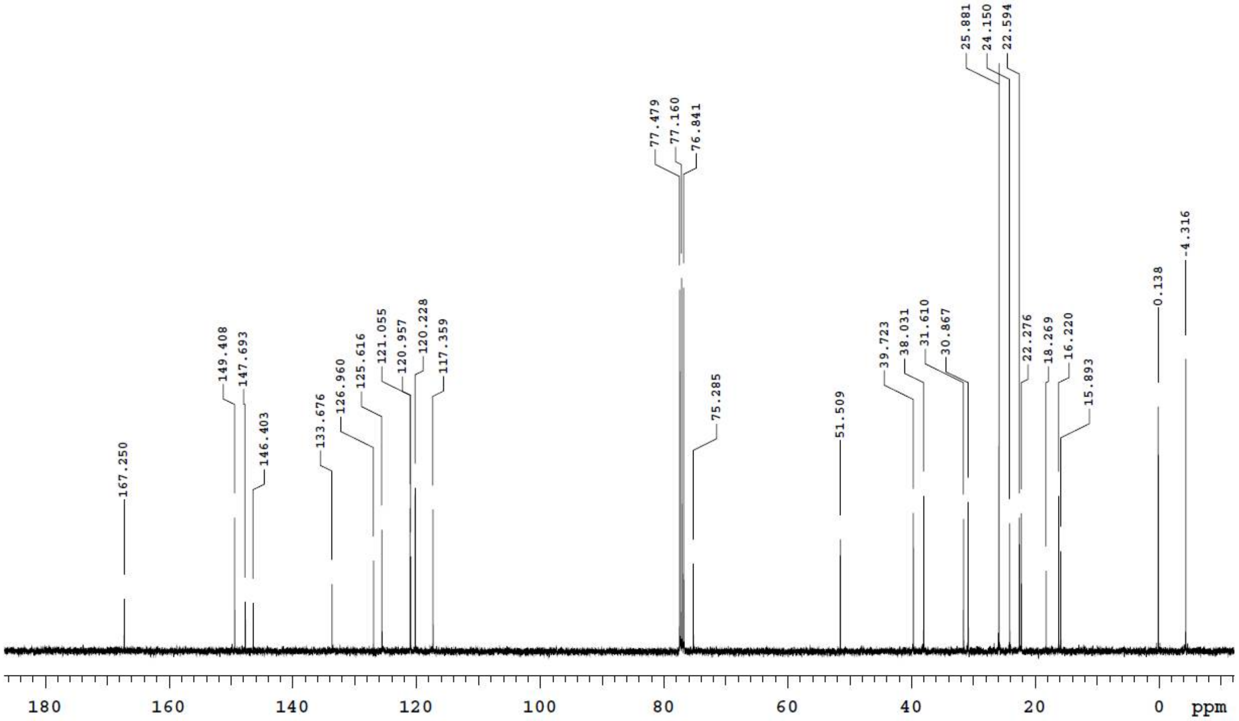

^{1}H and ^{13}C NMR spectra of 8b

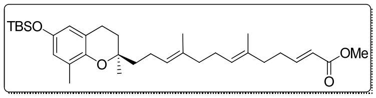

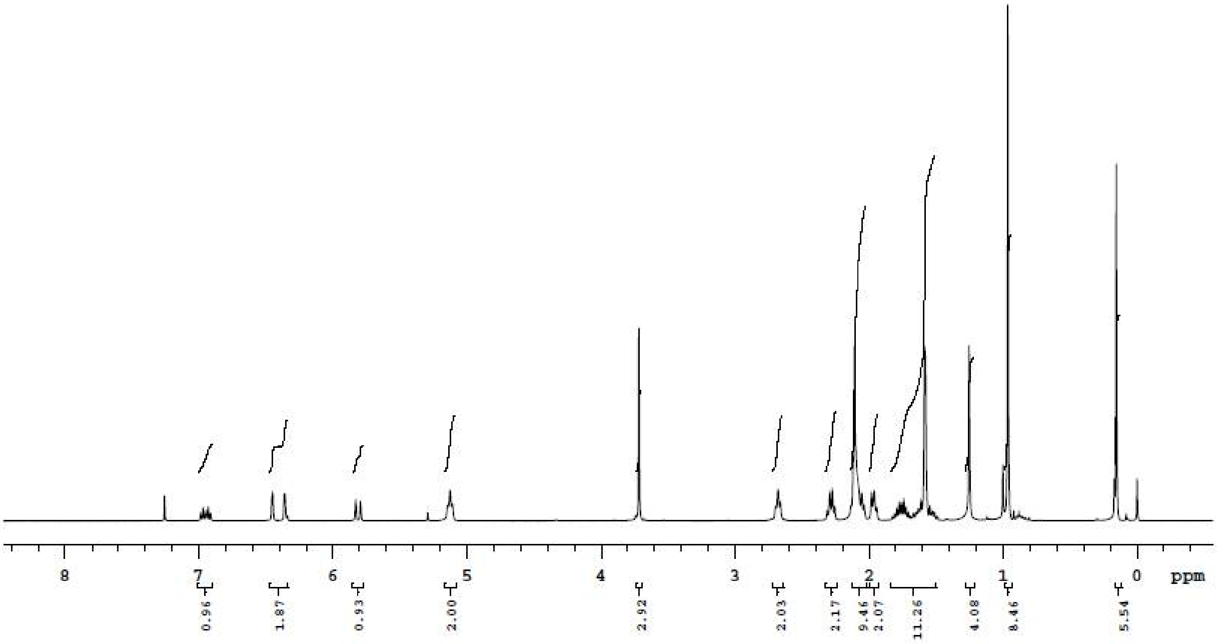

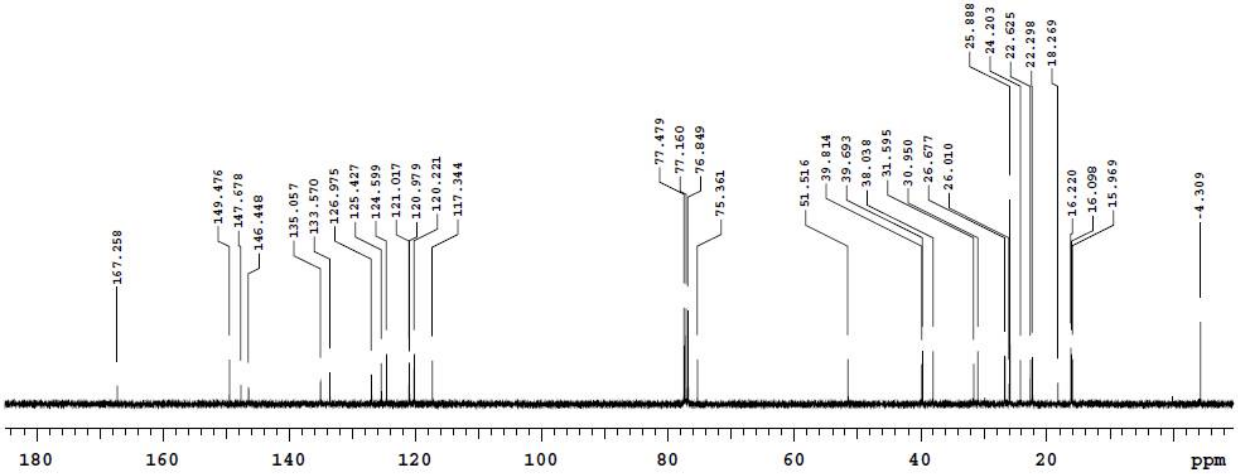

^{1}H and ^{13}C NMR spectra of 9a

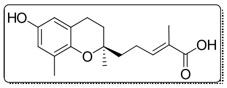

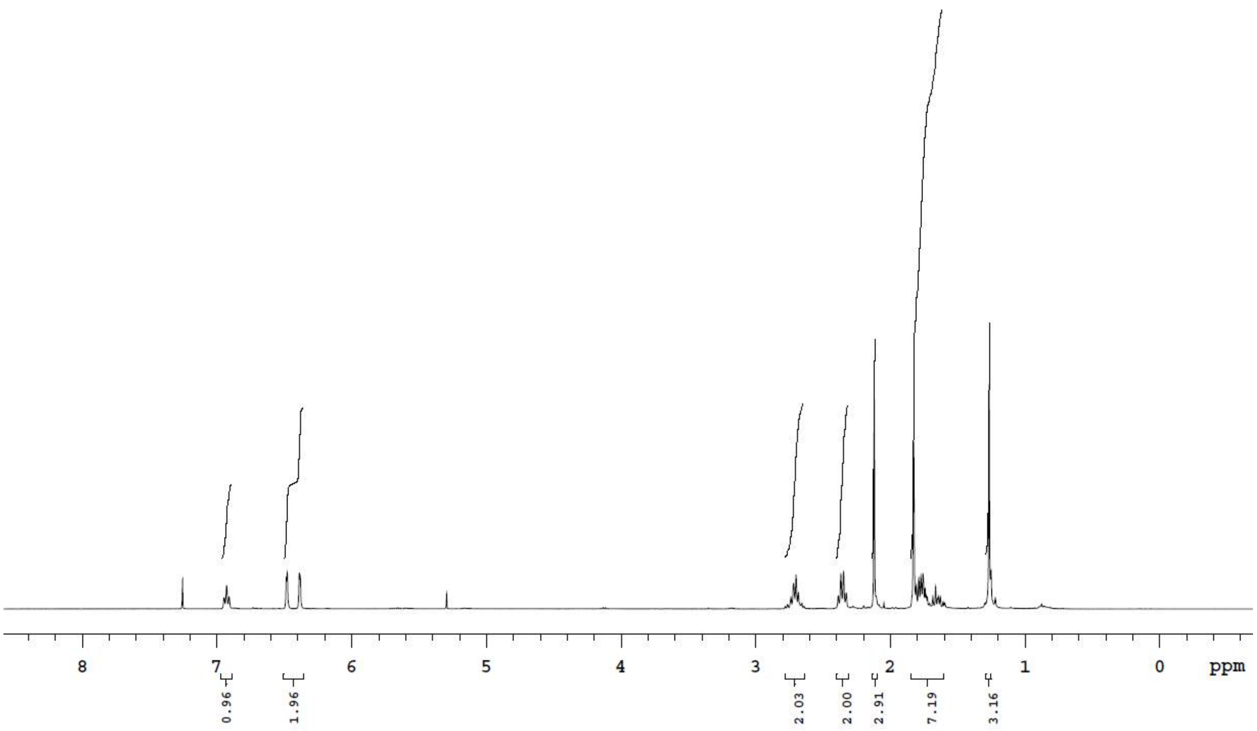

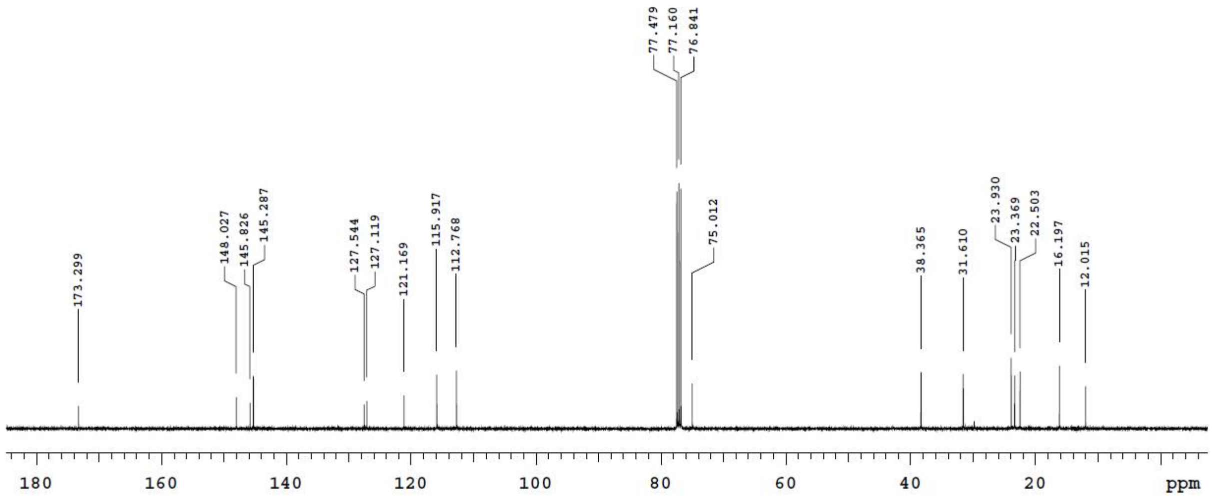

¹H and ¹³C NMR spectra of 10a

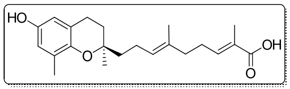

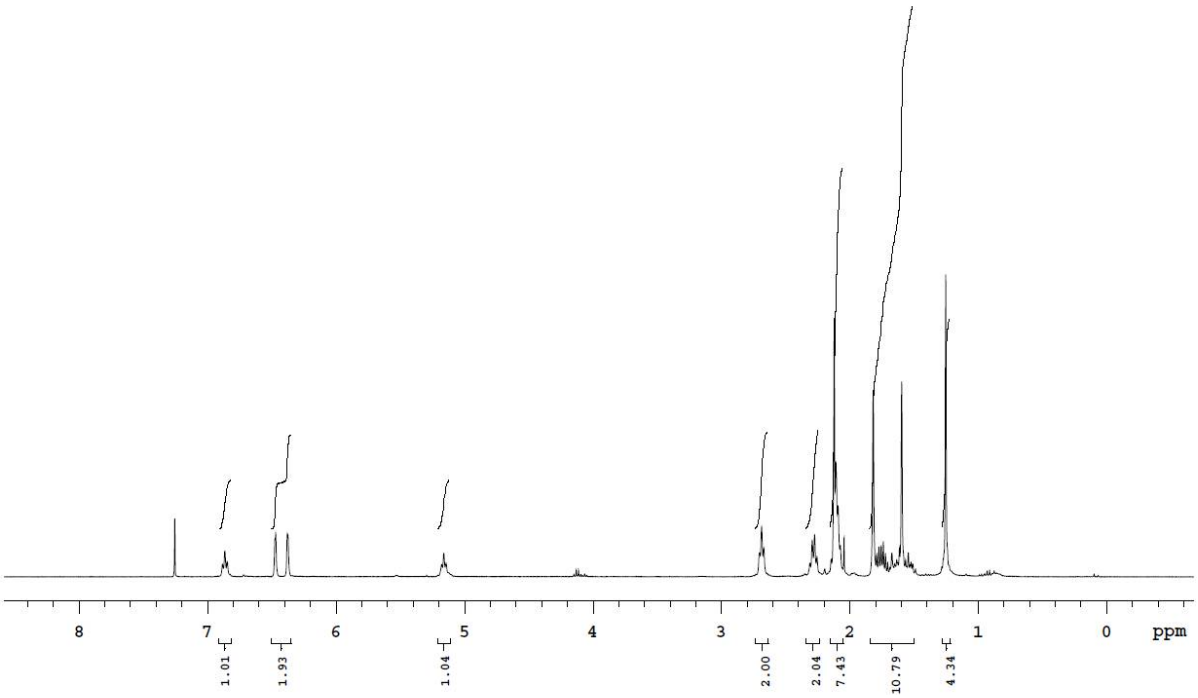

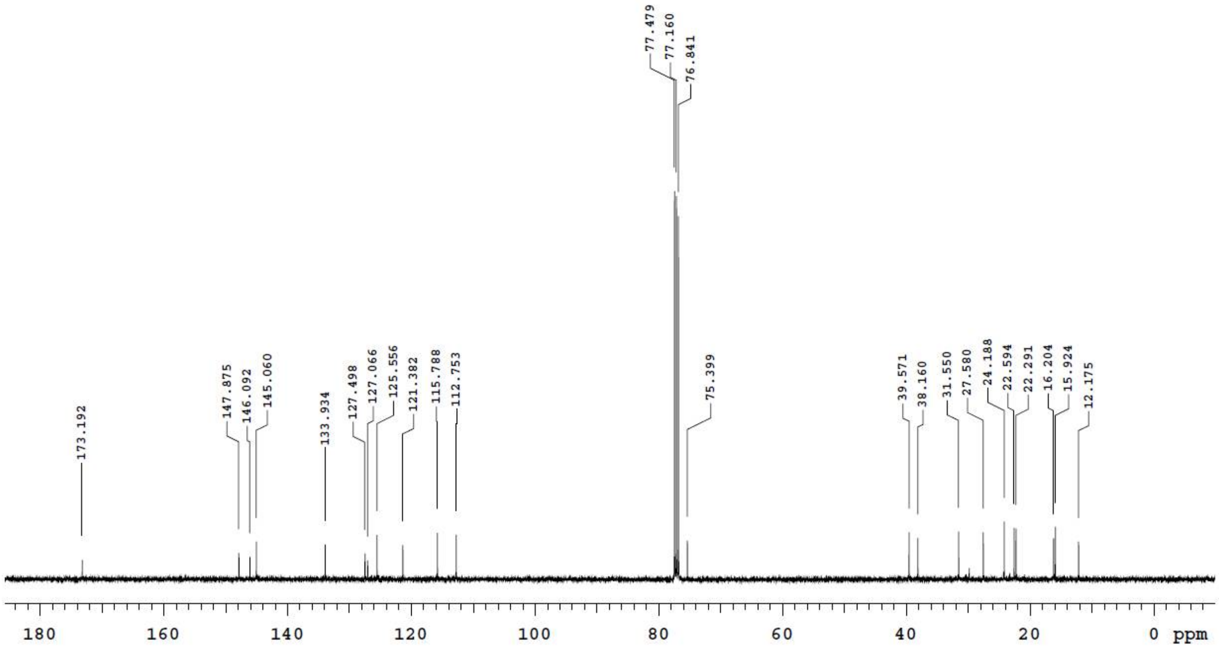

^{1}H and ^{13}C NMR spectra of 1

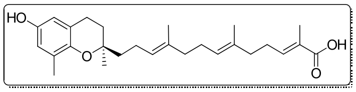

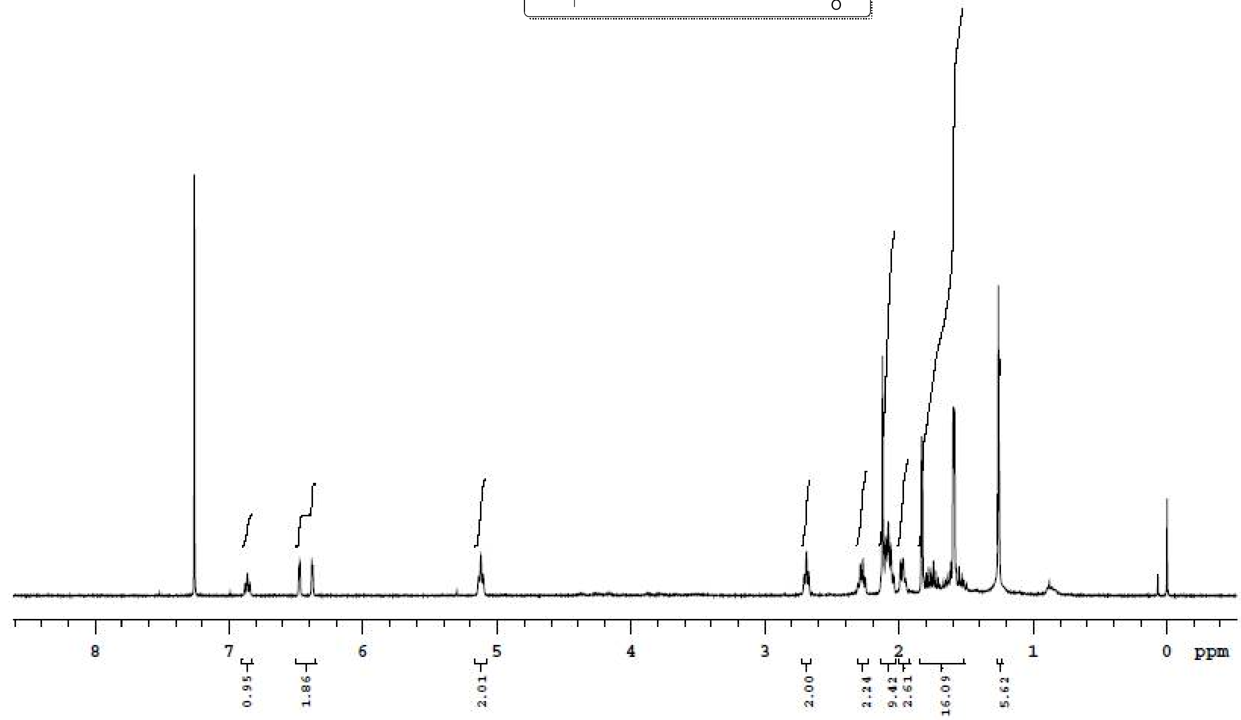

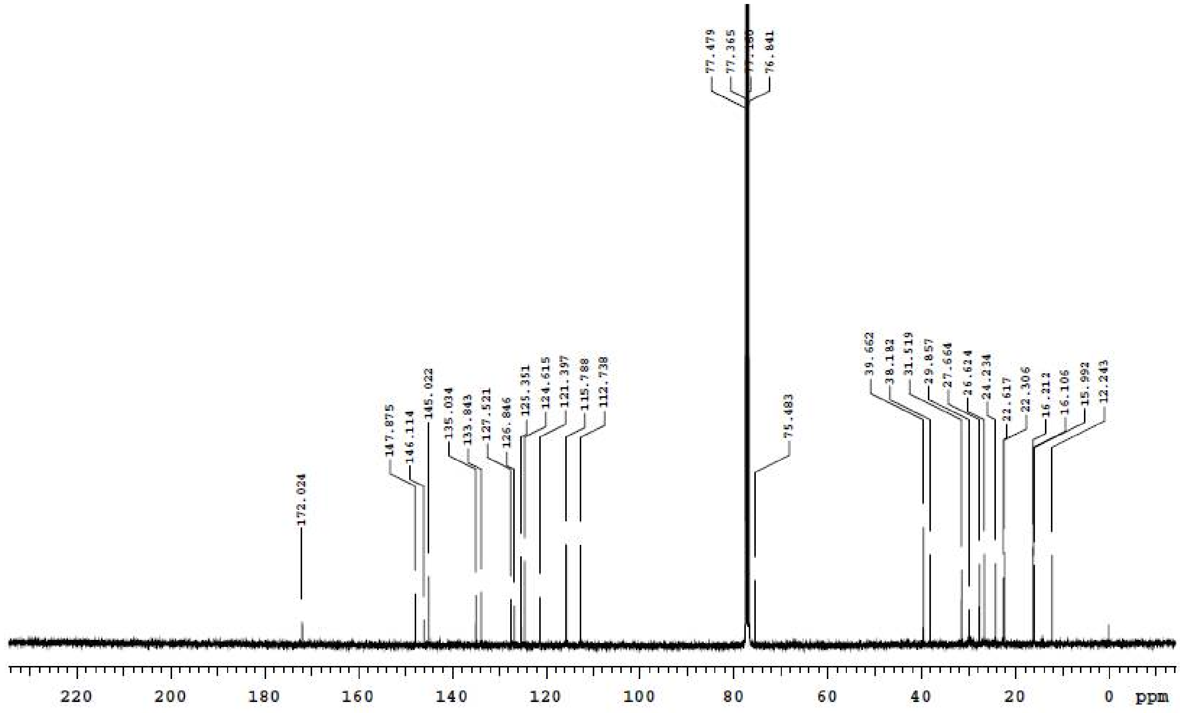

^{1}H and ^{13}C NMR spectra of 9b

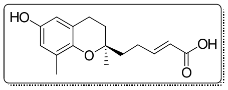

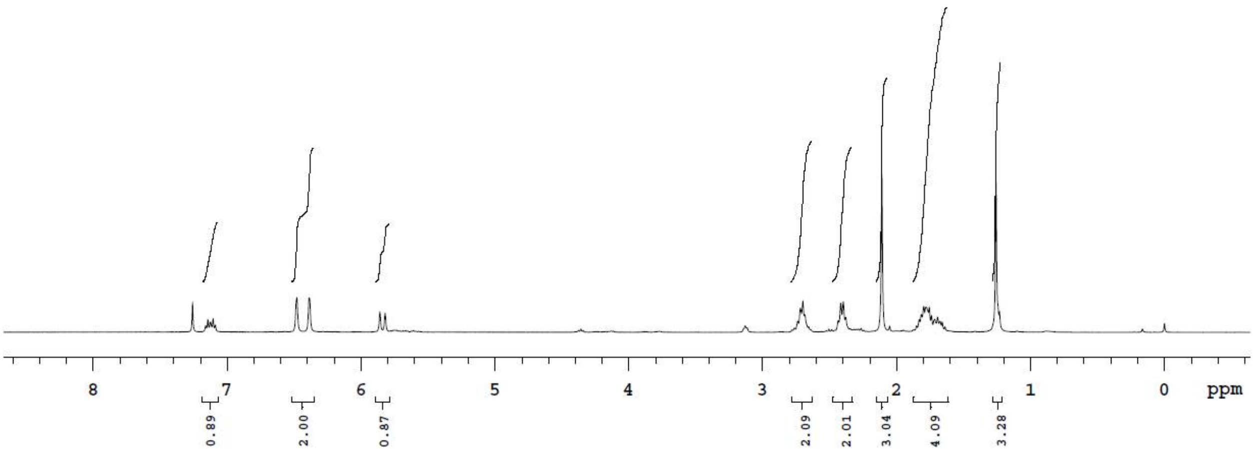

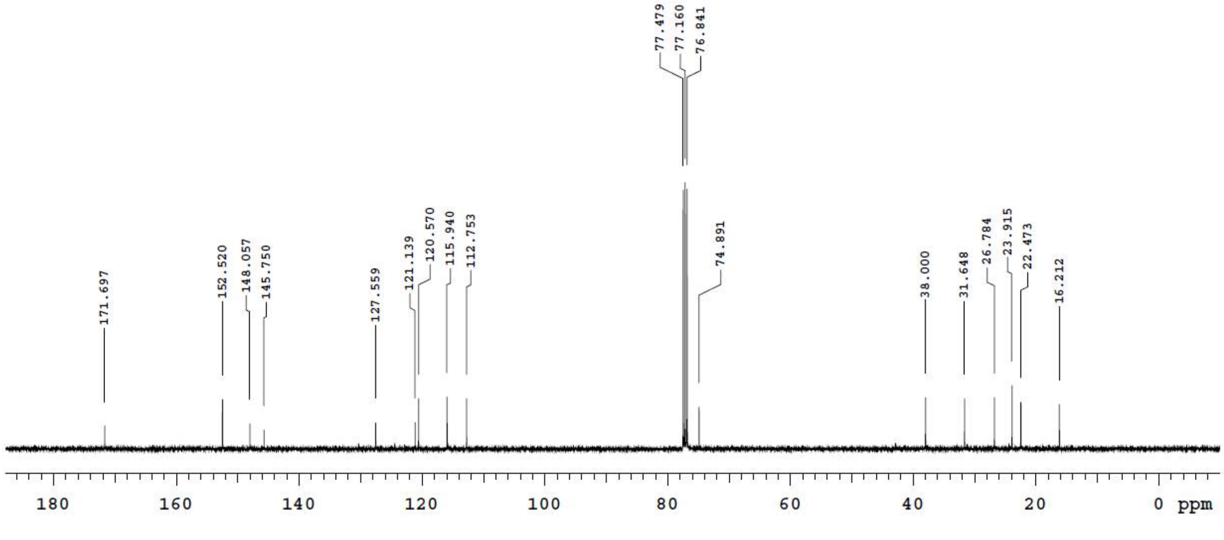

^{1}H and ^{13}C NMR spectra of 10b

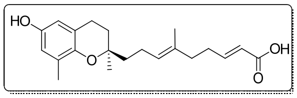

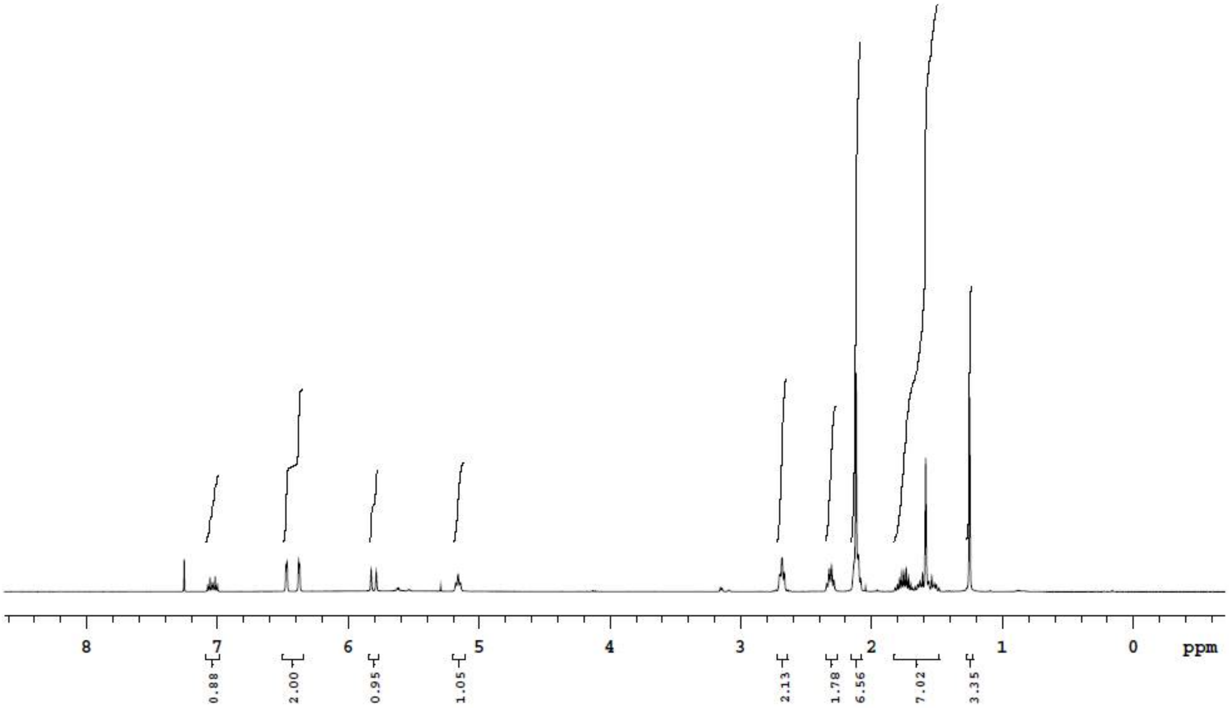

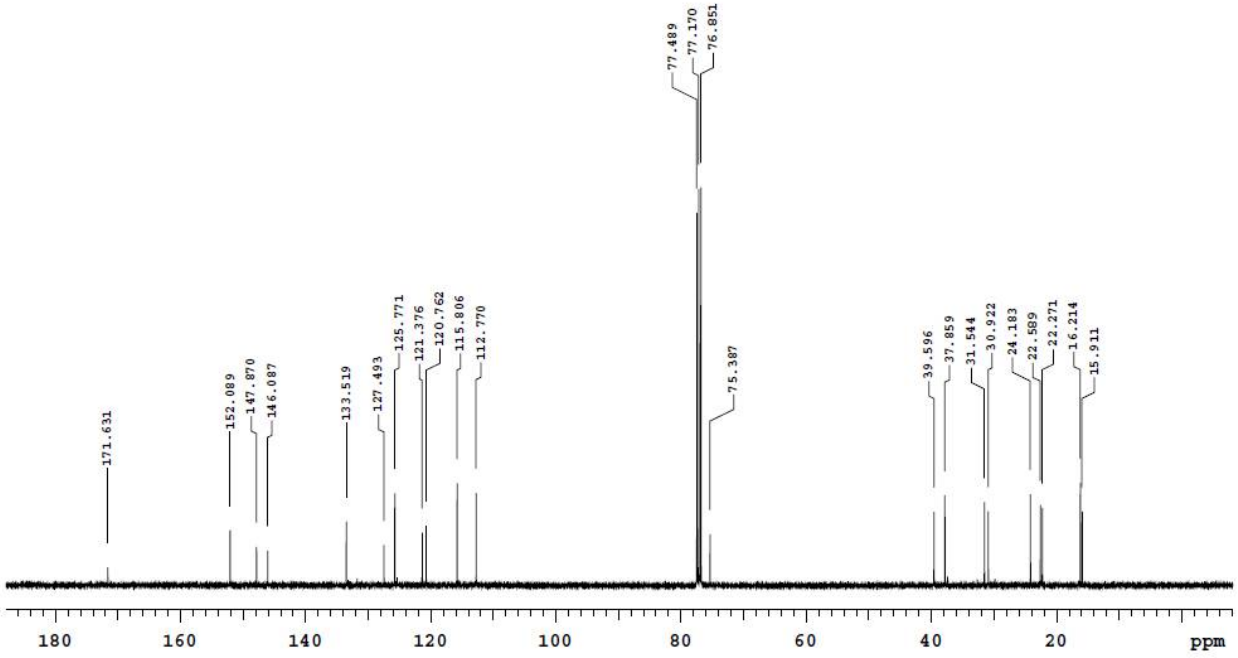

^{1}H and ^{13}C NMR spectra of 11

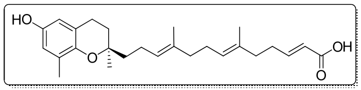

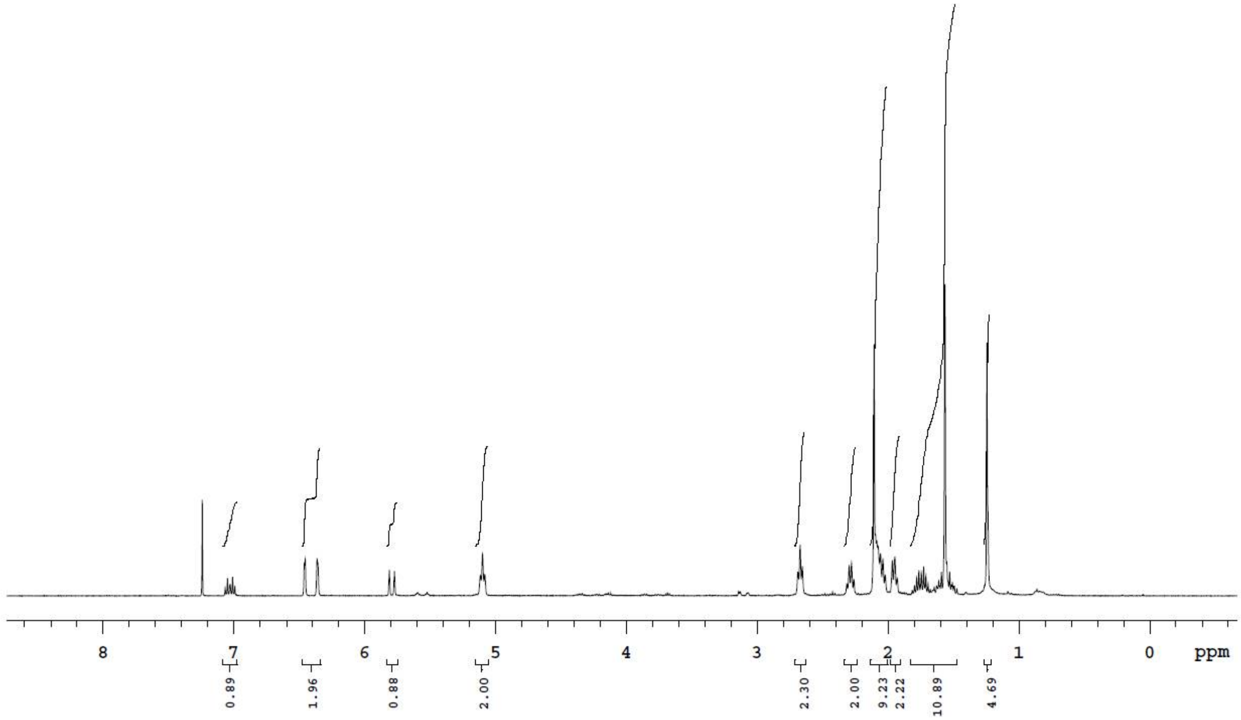

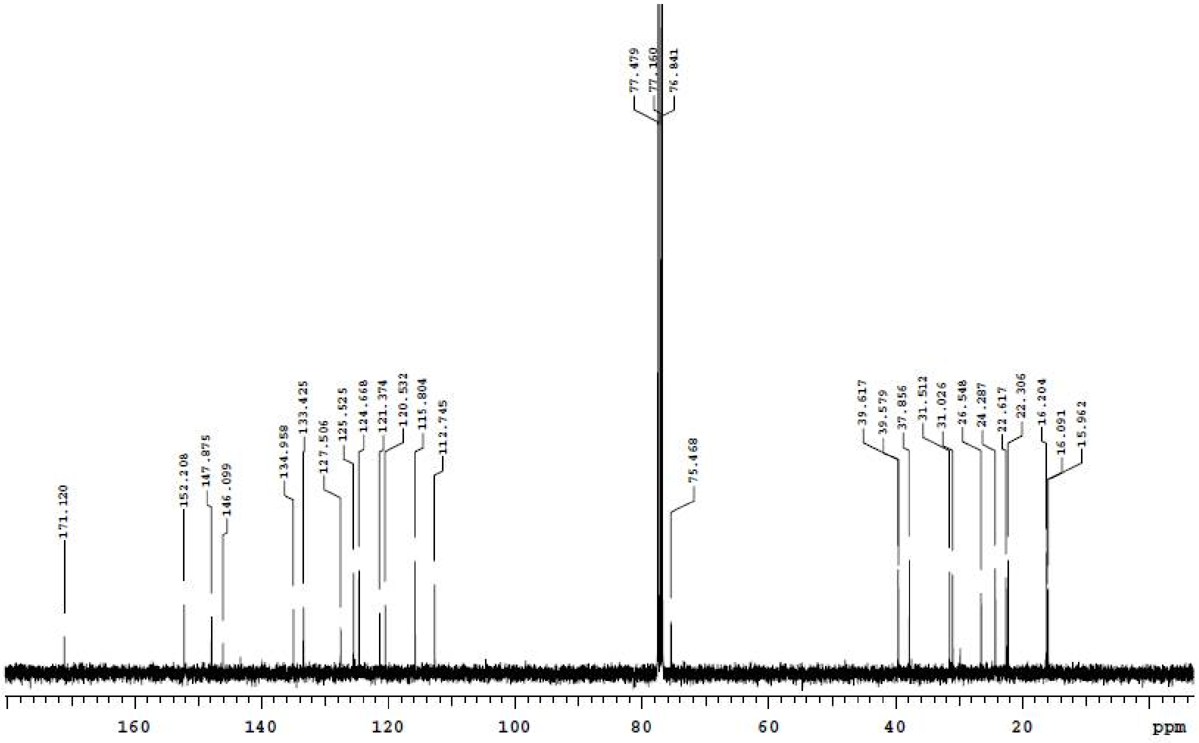

^{1}H and ^{13}C NMR spectra of 12a

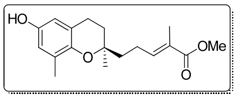

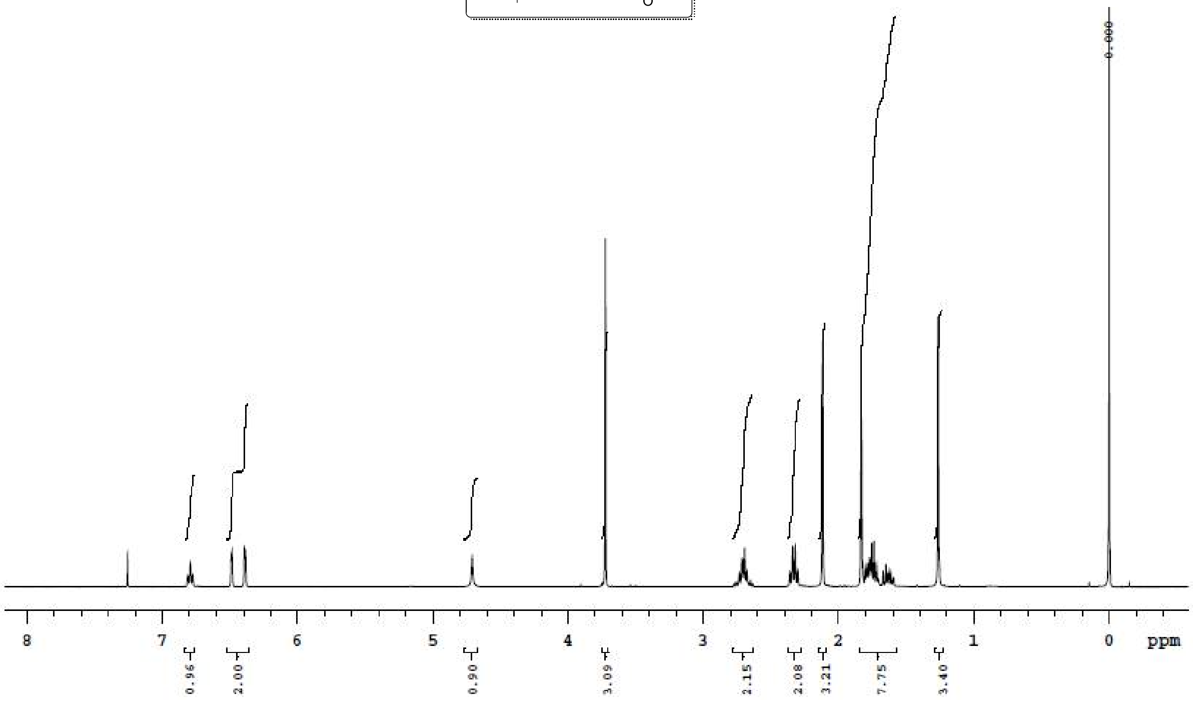

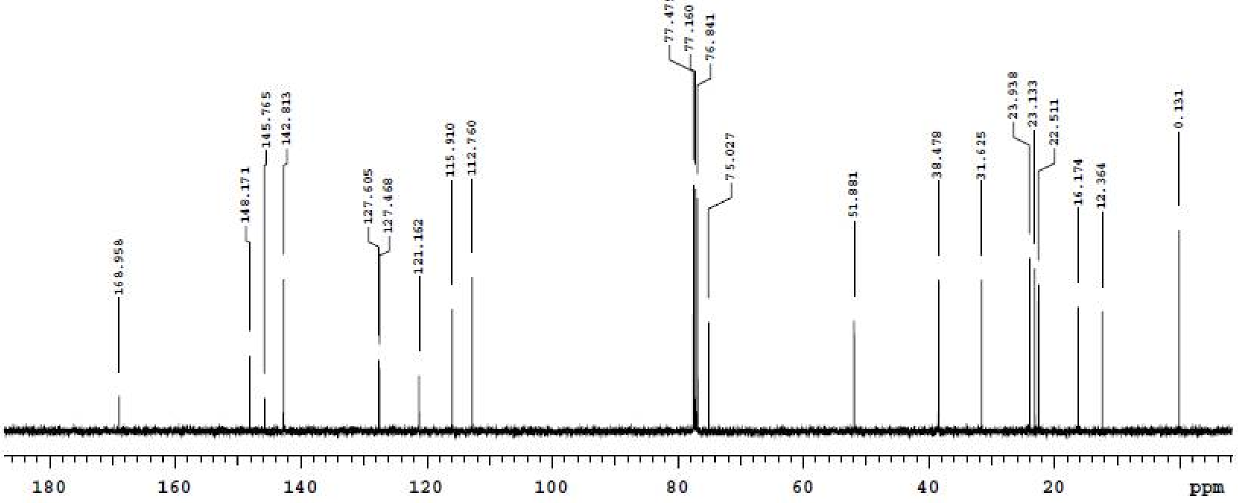

¹H and ¹³C NMR spectra of 13a

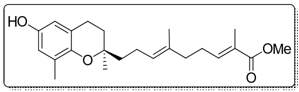

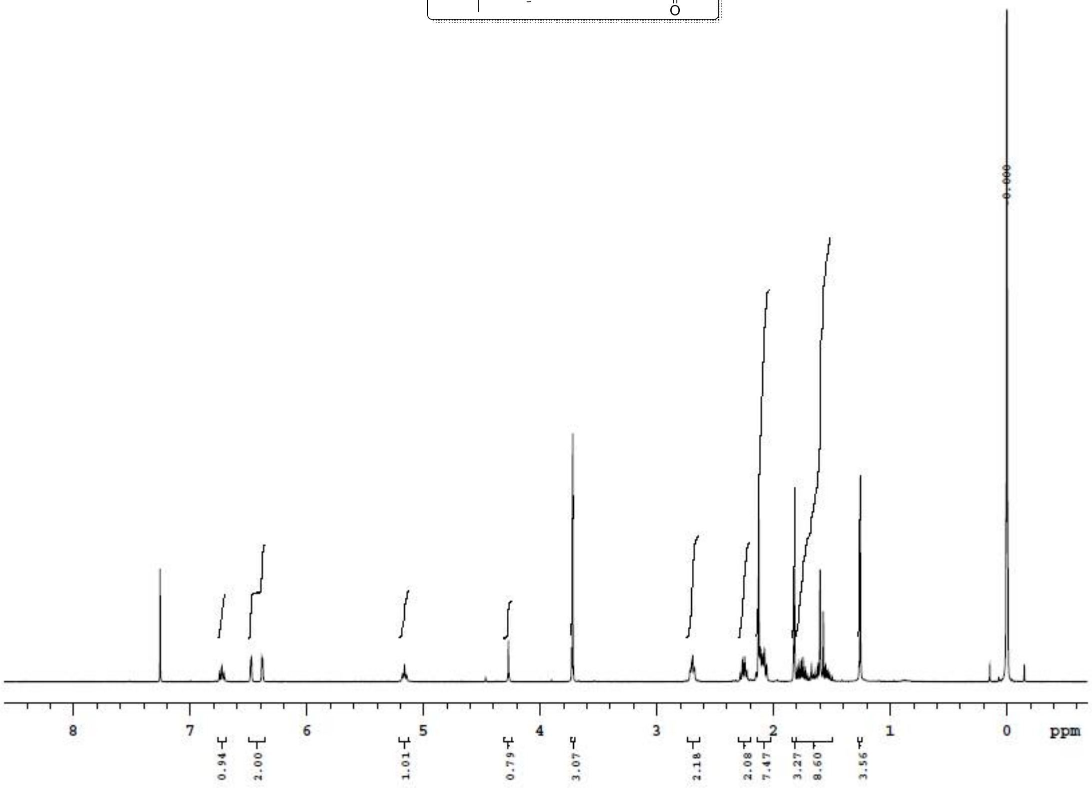

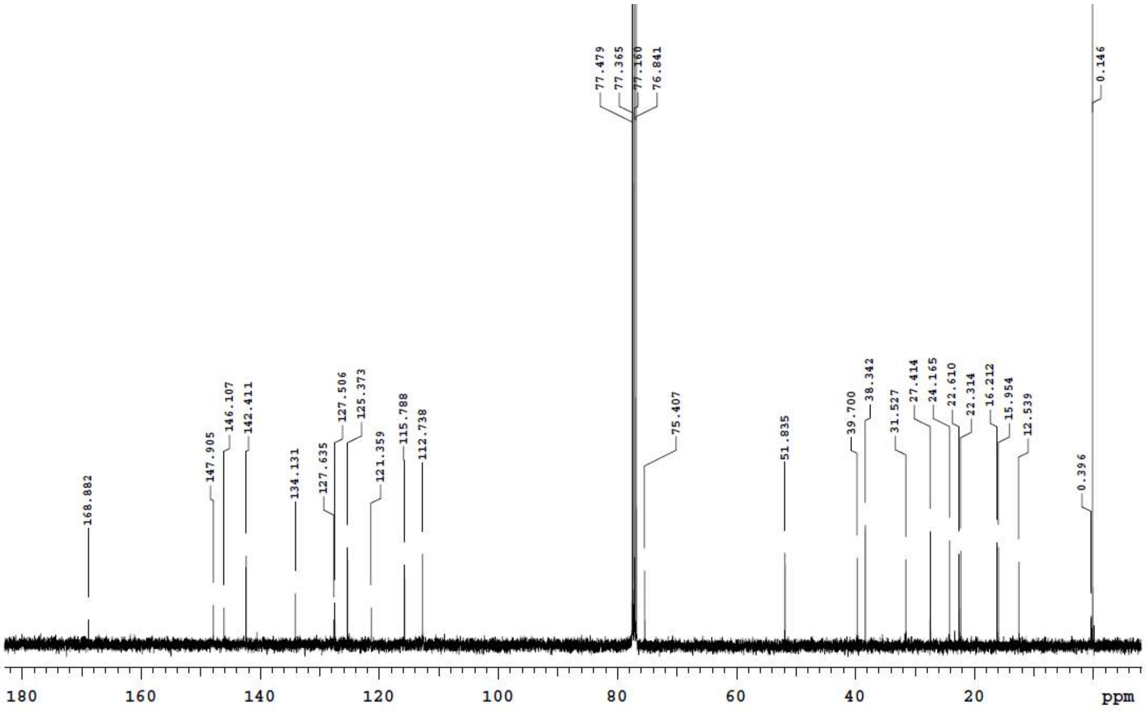

^{1}H and ^{13}C NMR spectra of 14a

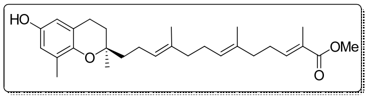

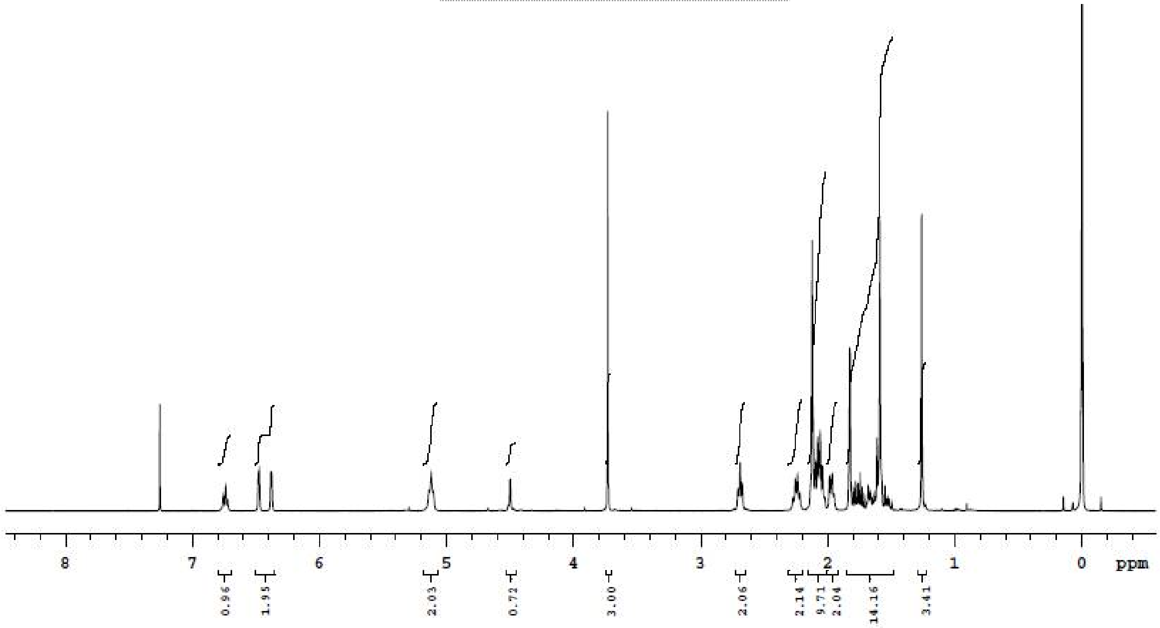

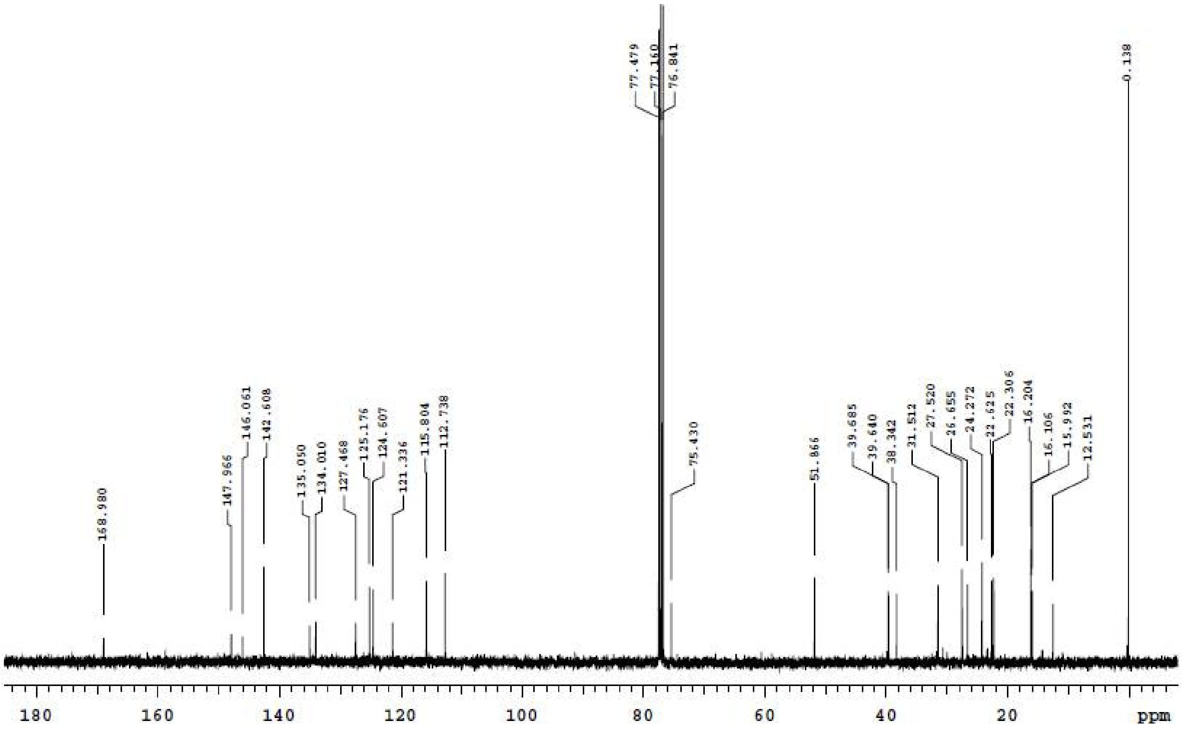

¹H and ¹³C NMR spectra of 12b

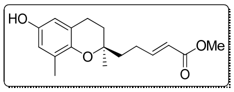

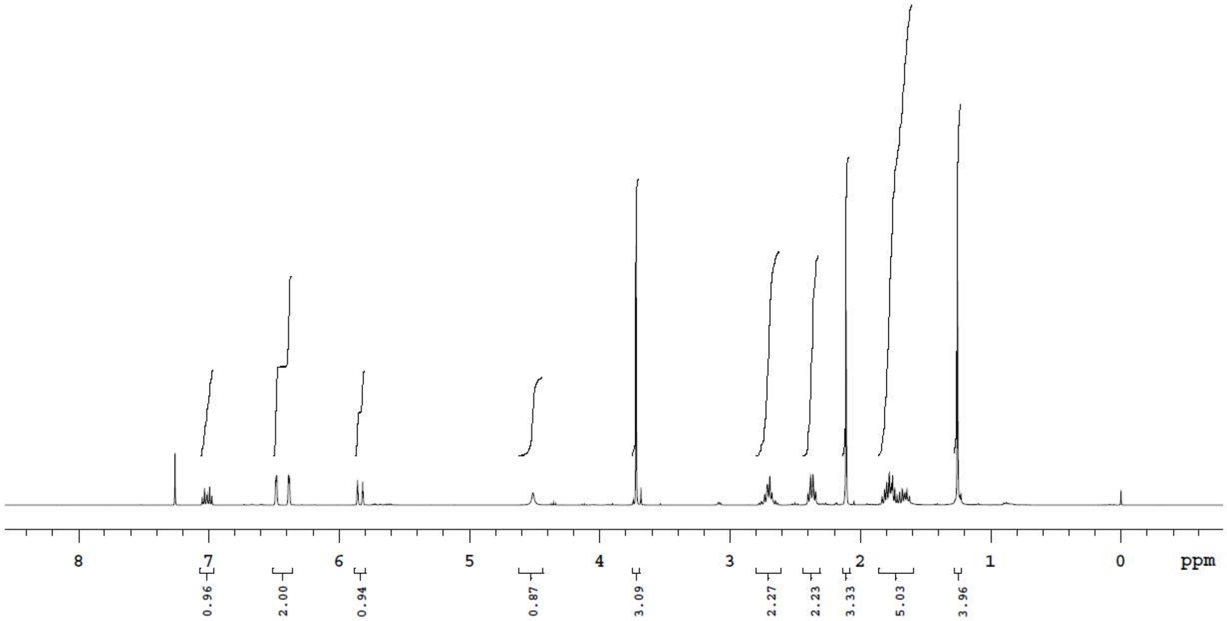

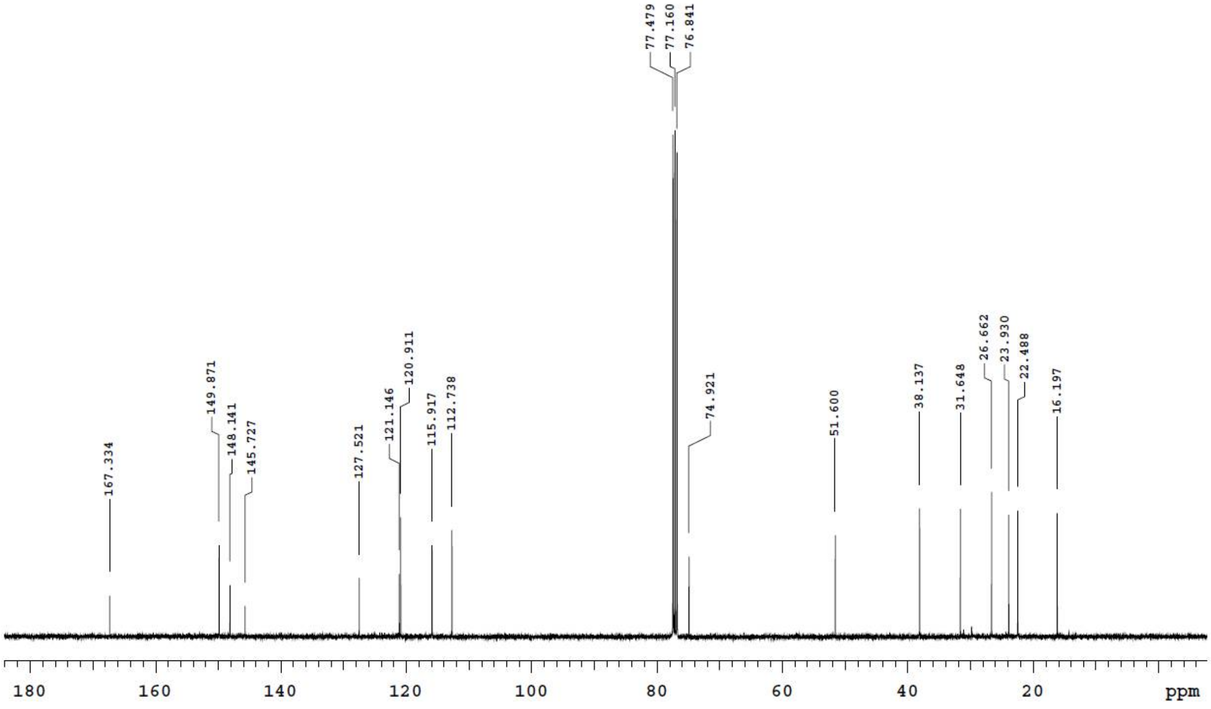

^{1}H and ^{13}C NMR spectra of 13b

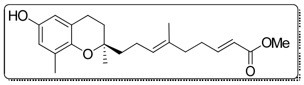

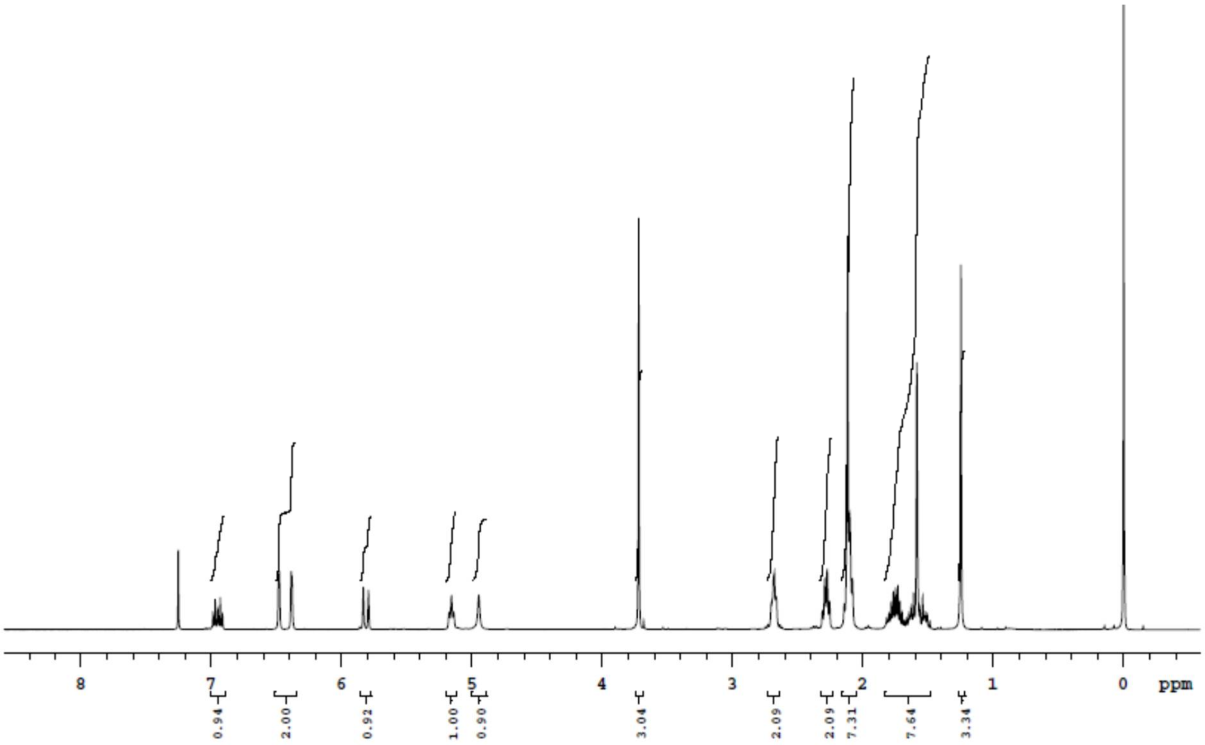

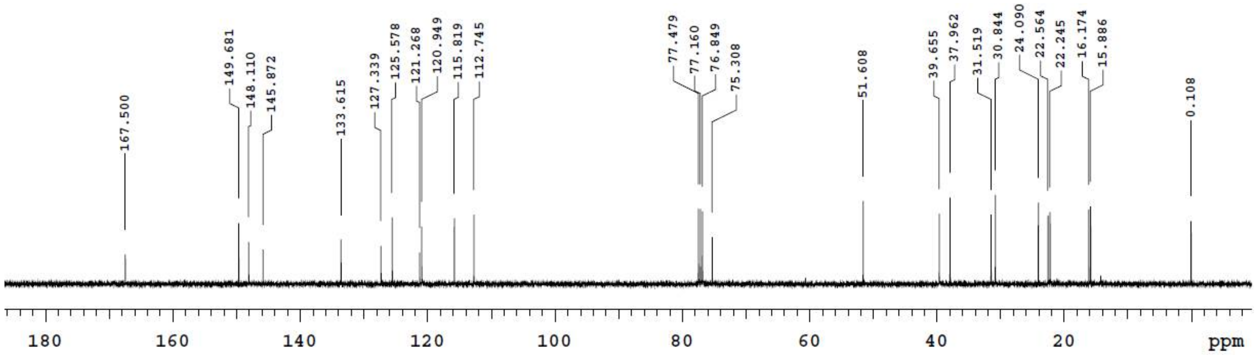

^{1}H and ^{13}C NMR spectra of 14b

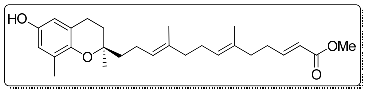

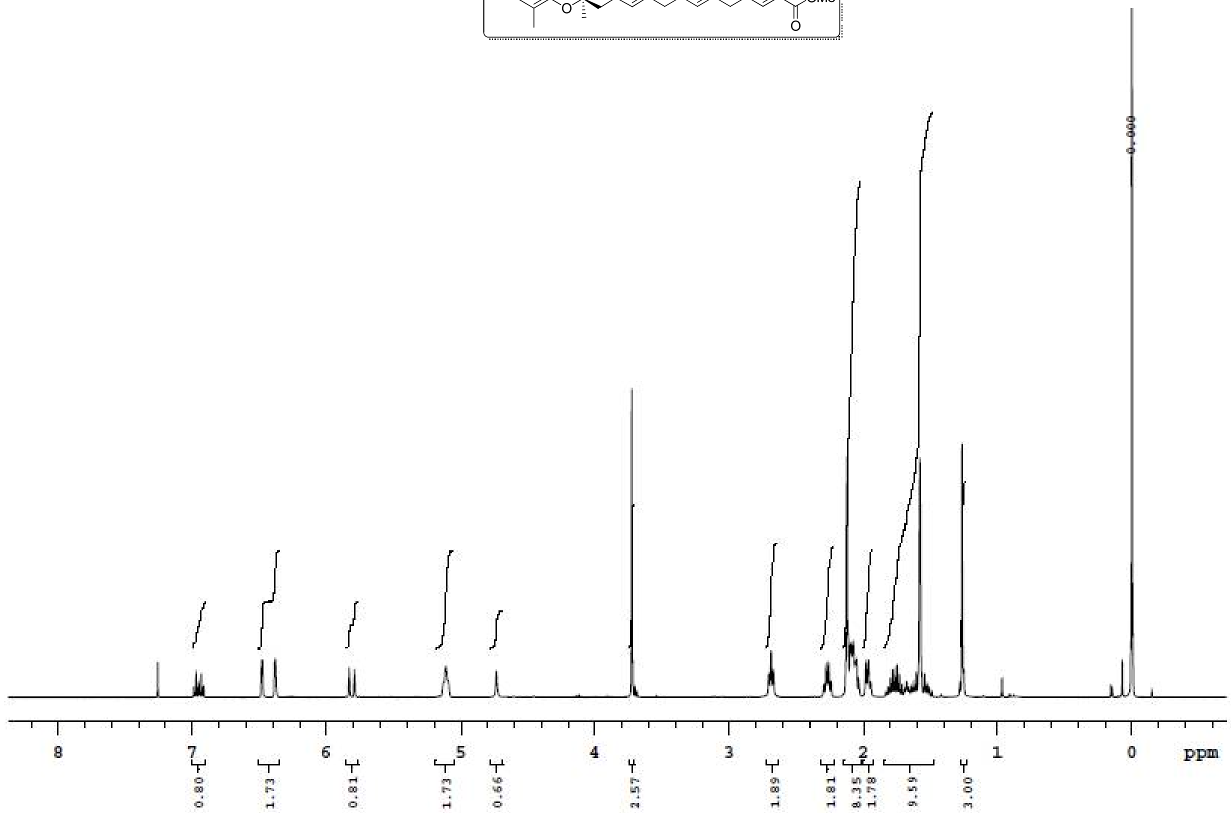

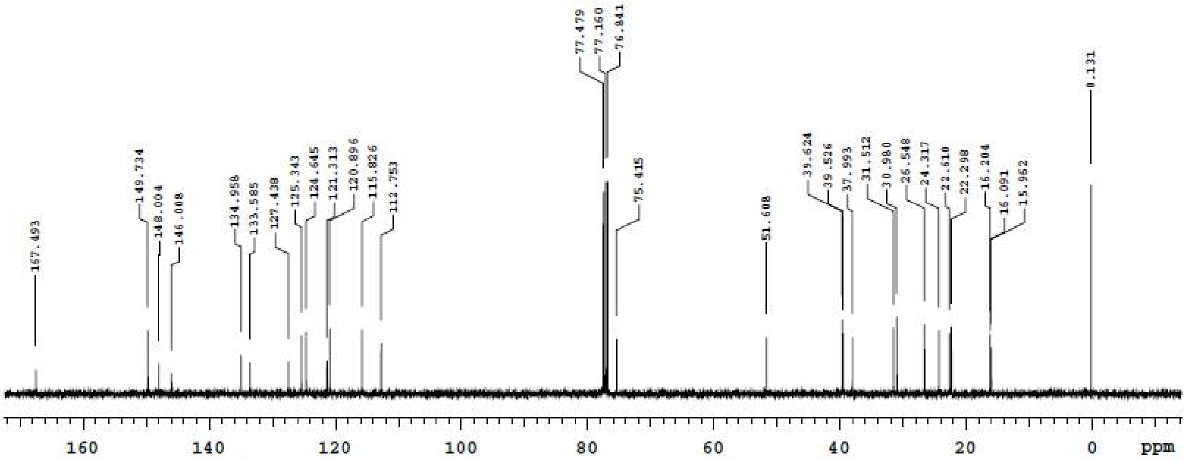

^{1}H and ^{13}C NMR spectra of 15

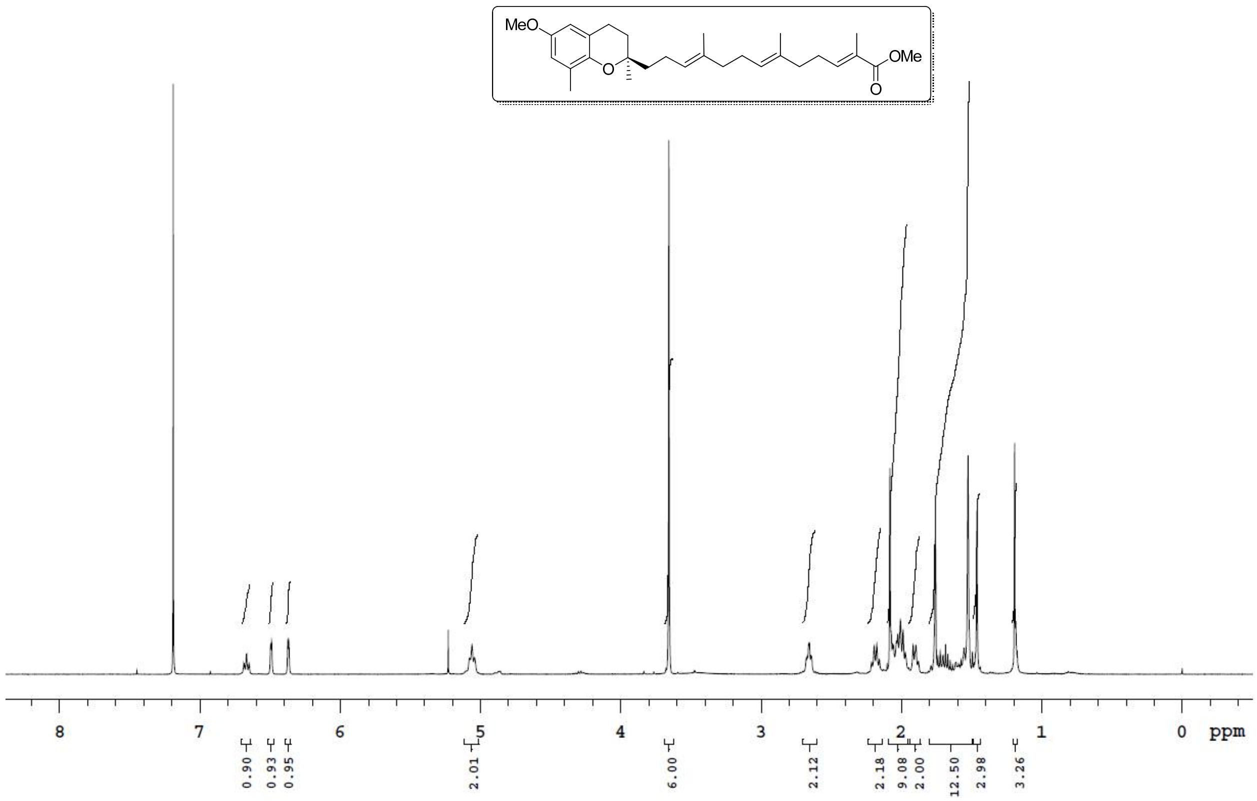

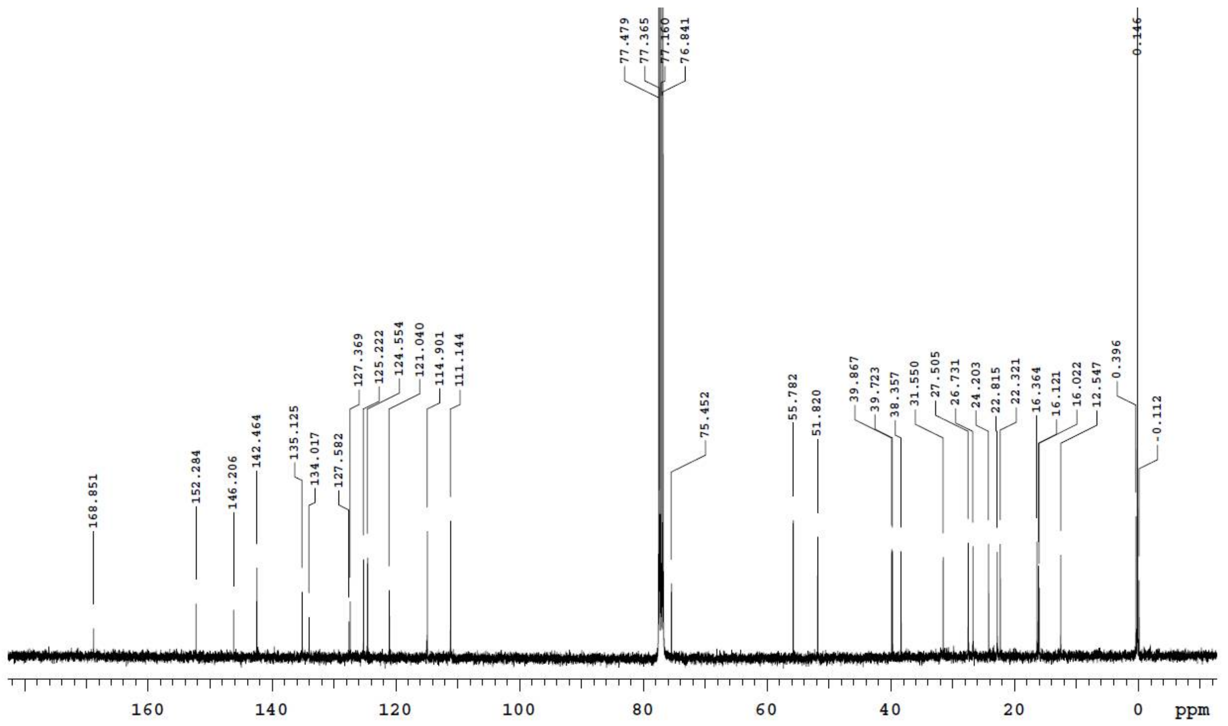

¹H and ¹³C NMR spectra of 16

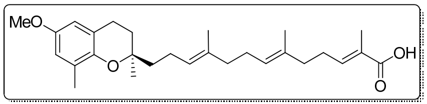

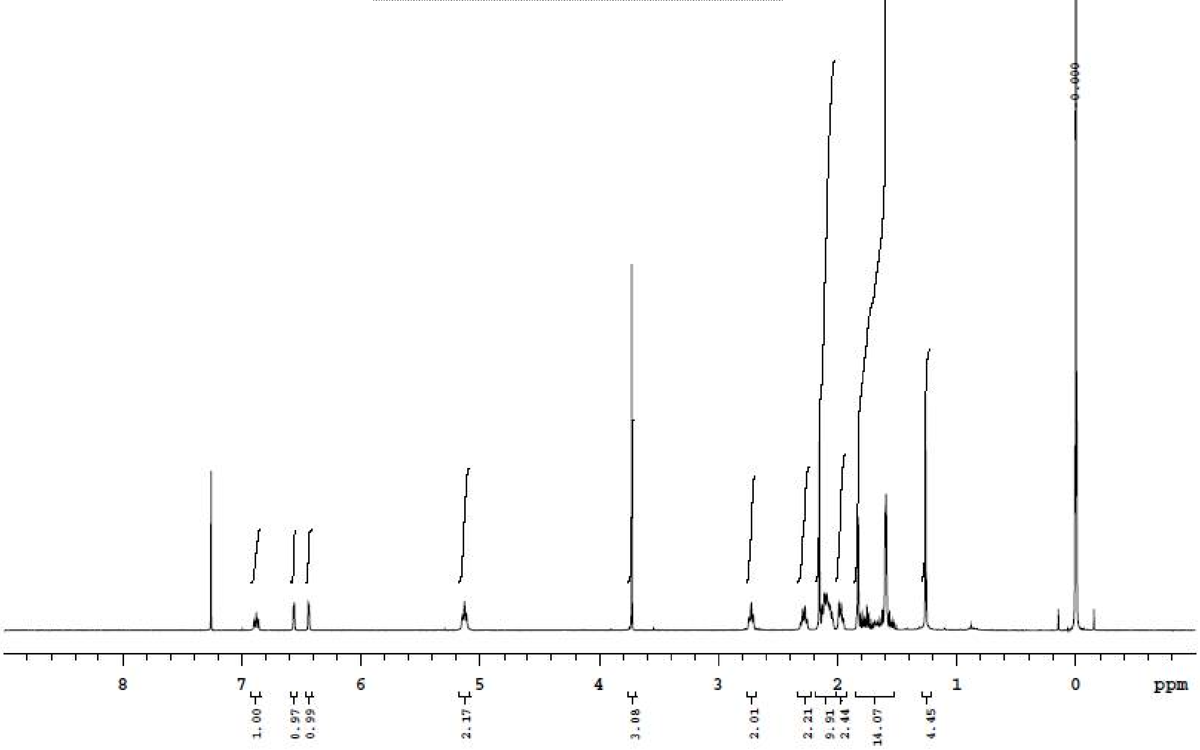

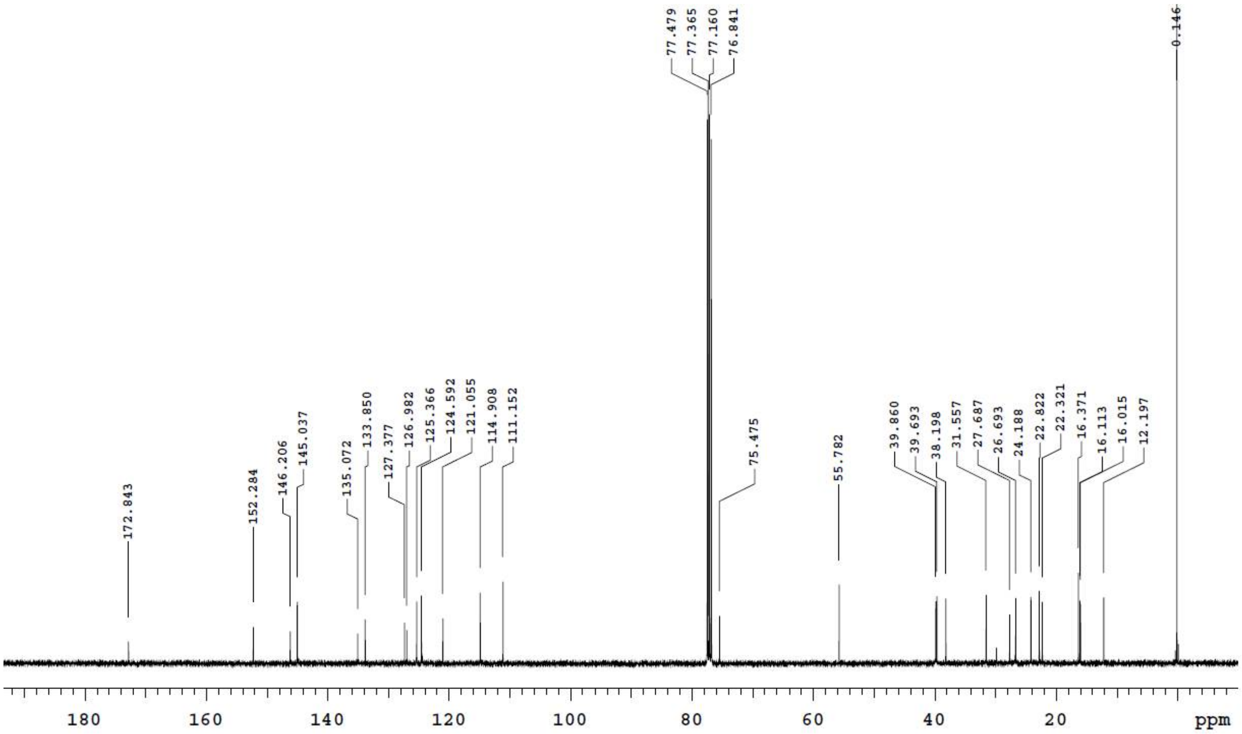

^{1}H and ^{13}C NMR spectra of 17

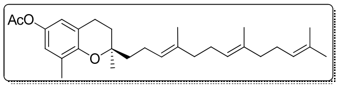

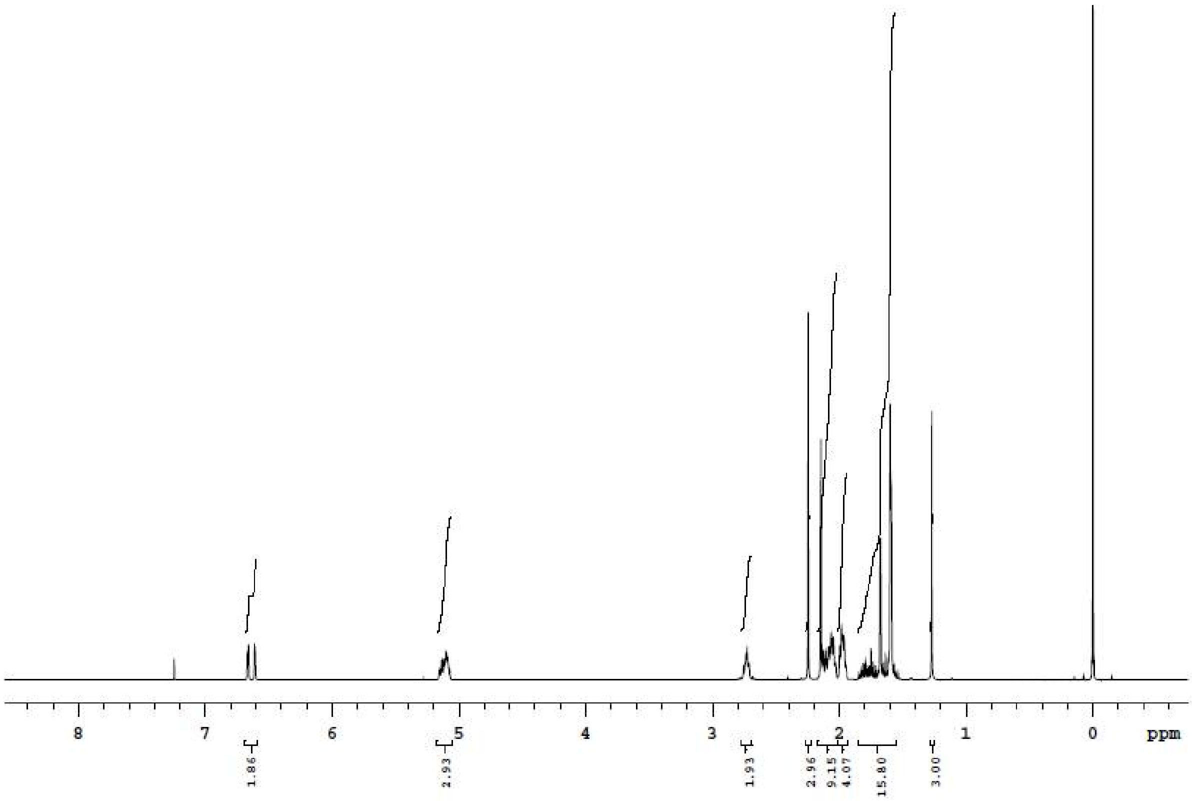

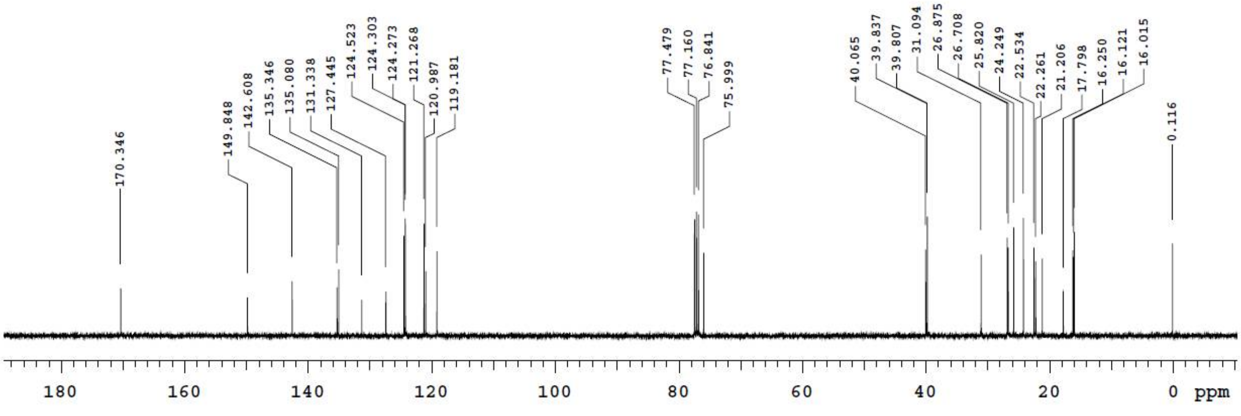

^{1}H and ^{13}C NMR spectra of 19

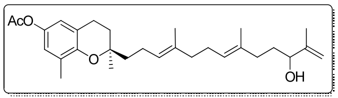

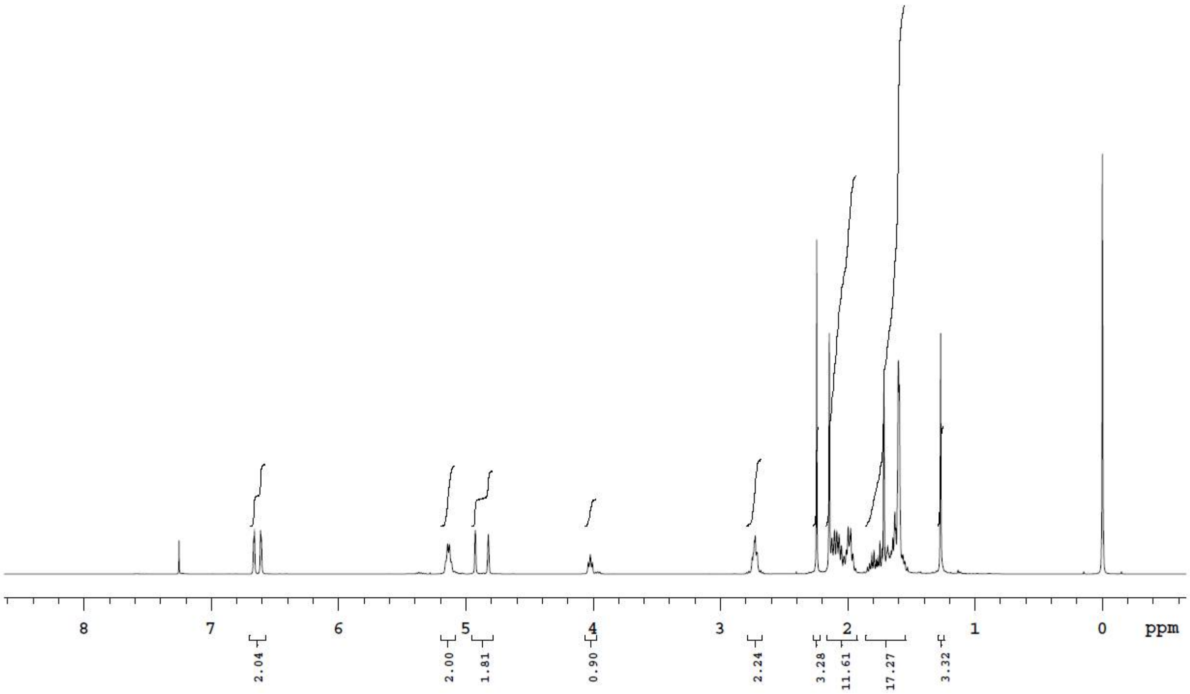

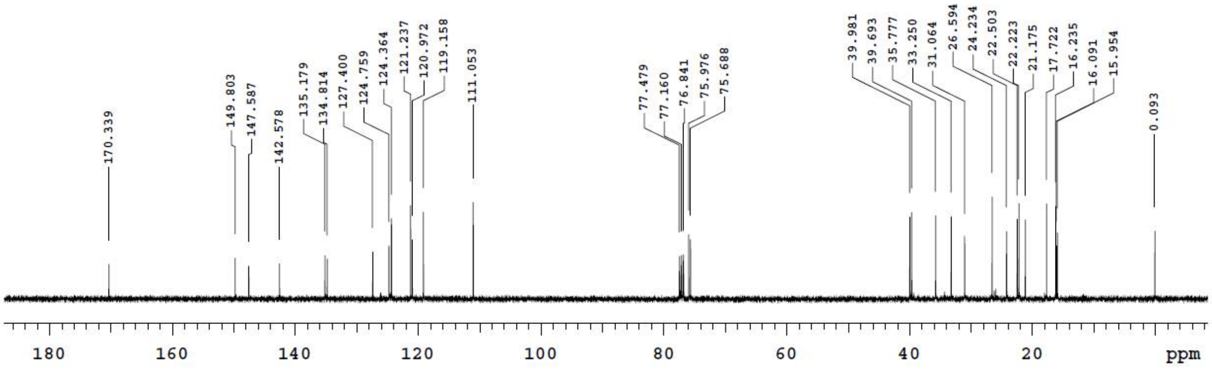

^{1}H and ^{13}C NMR spectra of 20

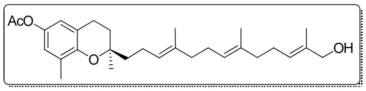

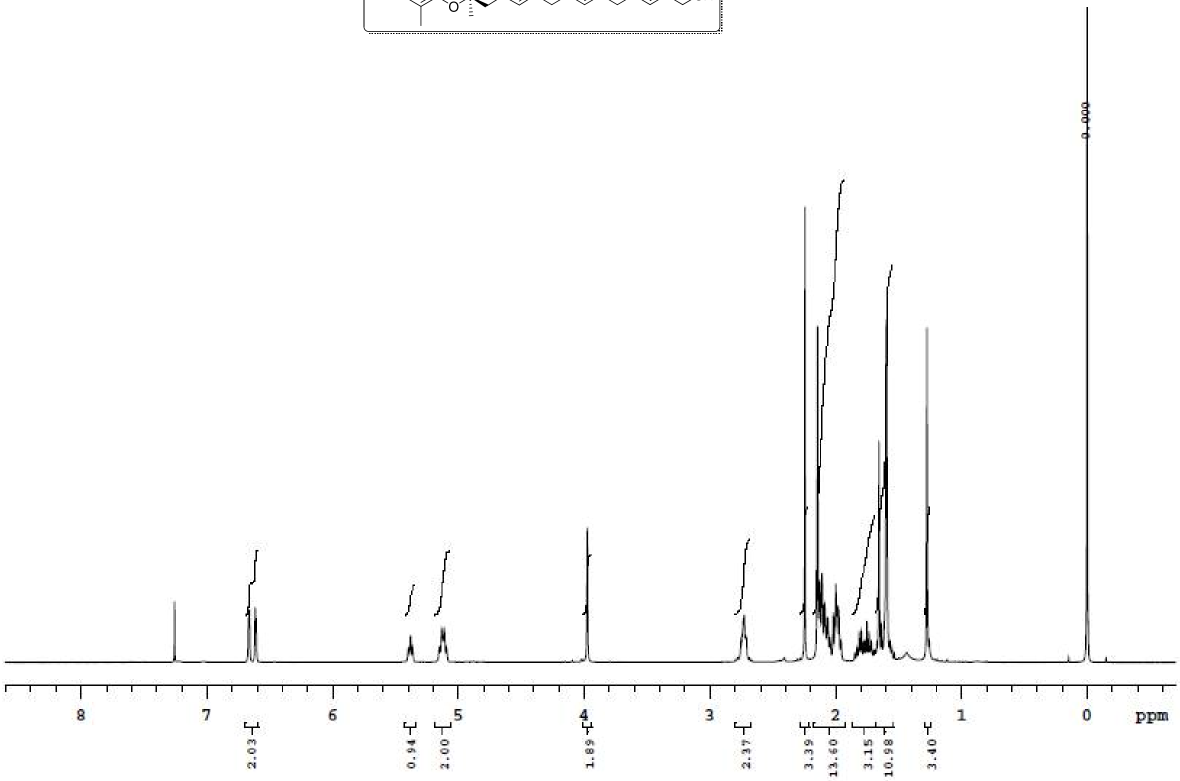

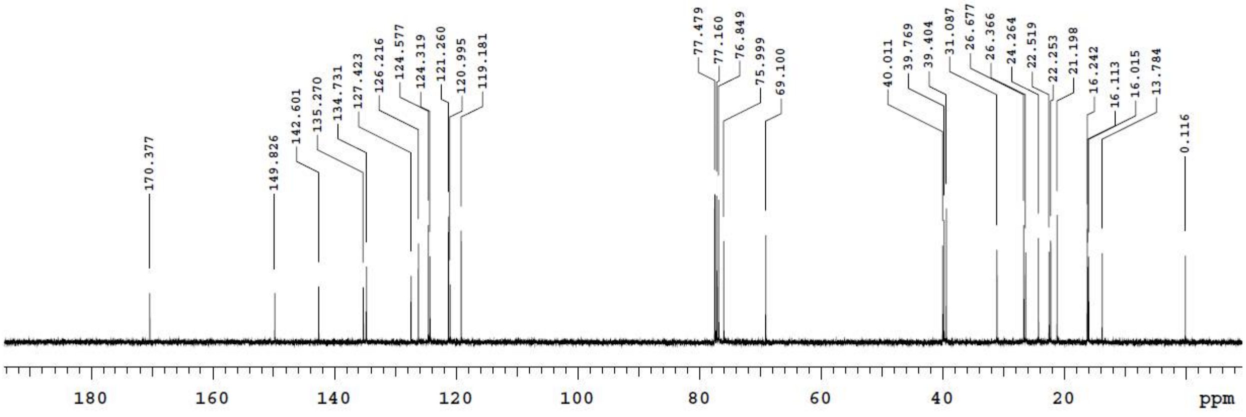

^{1}H and ^{13}C NMR spectrum of 21

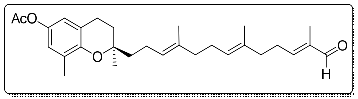

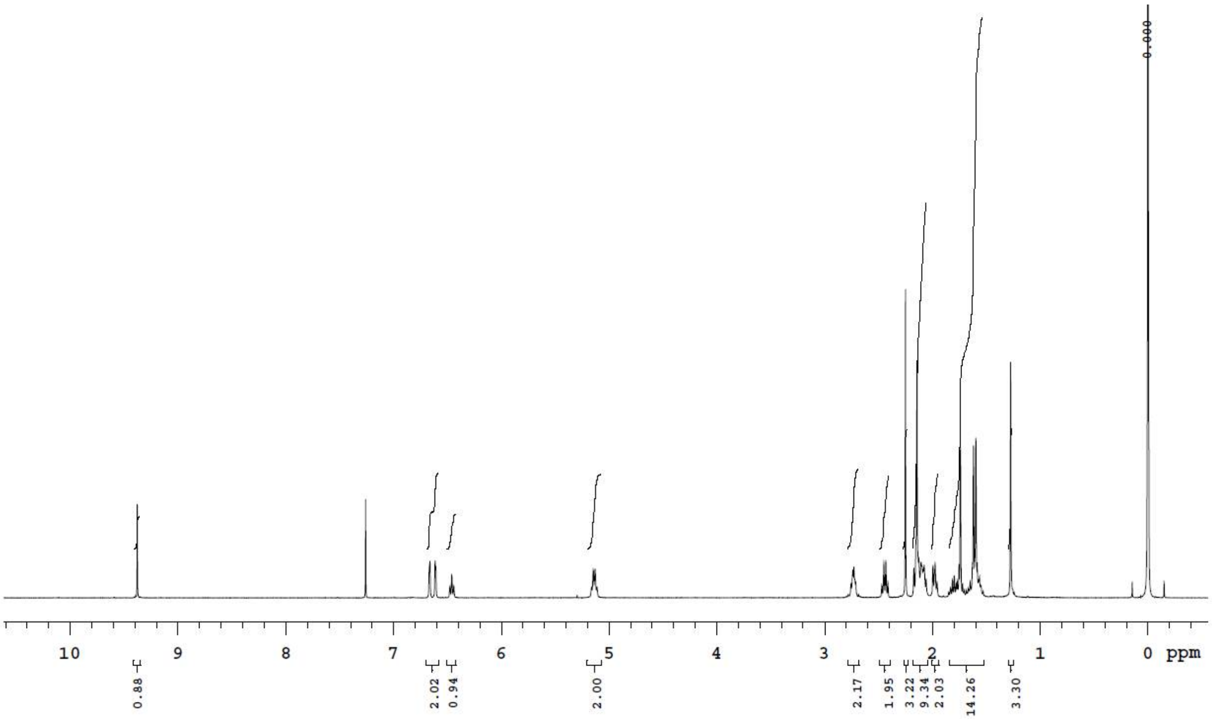

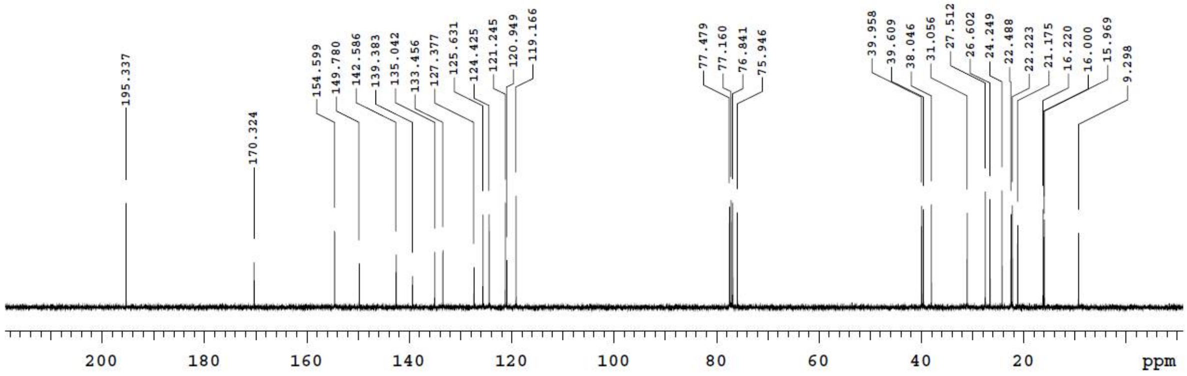

^{1}H and ^{13}C NMR spectrum of 22

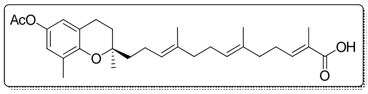

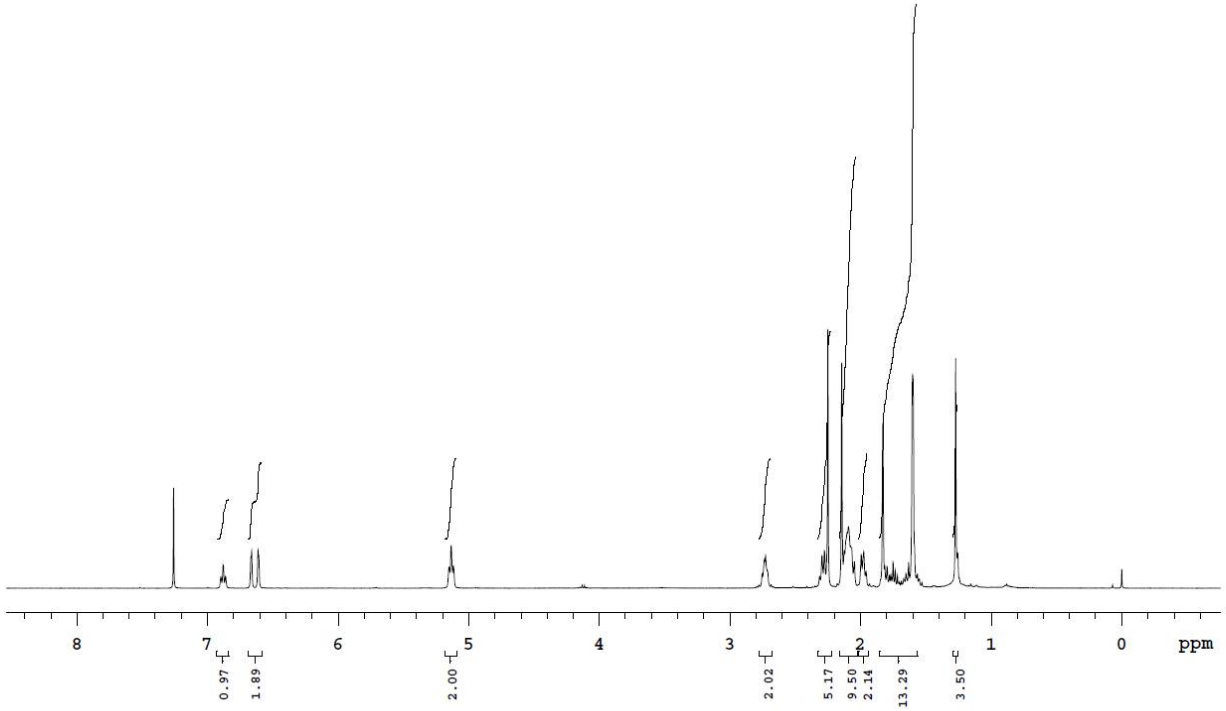

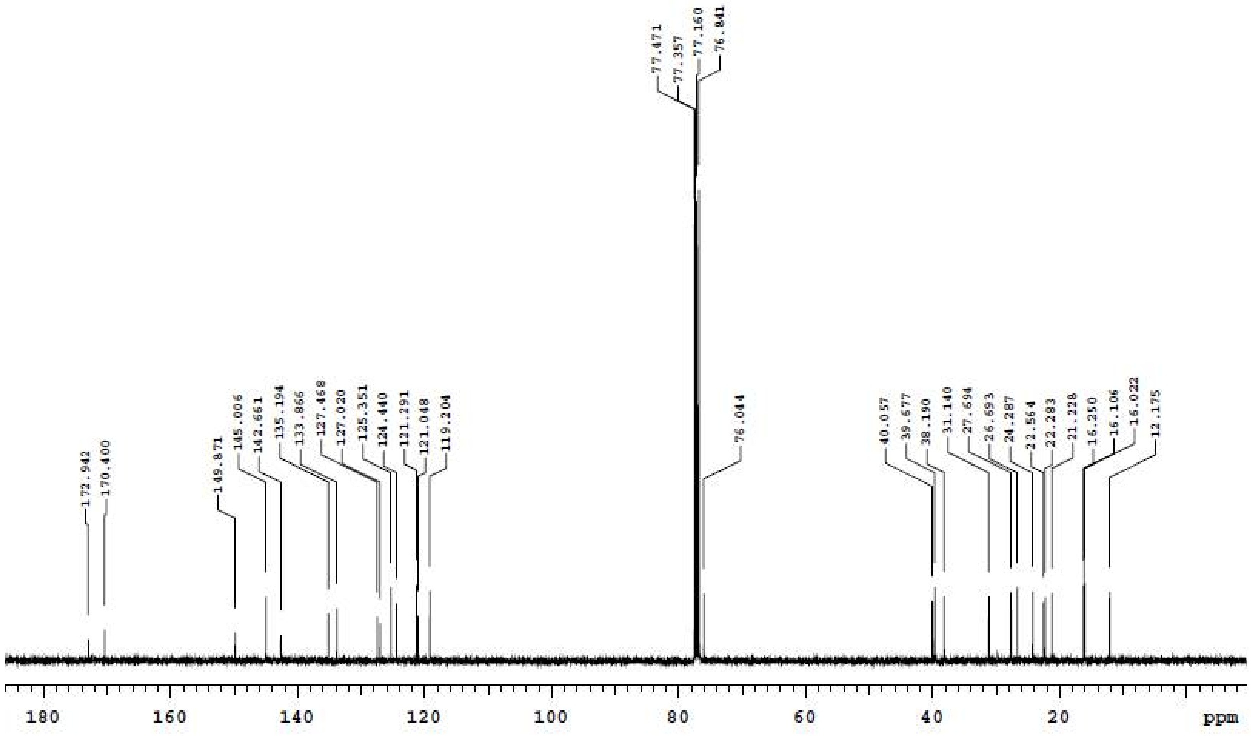

Supplement: Supplementary file 1 [file molecules-25-05847-s001.pdf]
